# Targeting cannabinoid receptor 1 for antagonism in pro-fibrotic alveolar macrophages mitigates pulmonary fibrosis

**DOI:** 10.1172/jci.insight.187967

**Published:** 2025-07-03

**Authors:** Abhishek Basu, Muhammad Arif, Kaelin M. Wolf, Madeline Behee, Natalie Johnson, Lenny Pommerolle, Ricardo H. Pineda, John Sembrat, Charles N. Zawatsky, Szabolcs Dvorácskó, Nathan J. Coffey, Joshua K. Park, Seray B. Karagoz, Grzegorz Godlewski, Tony Jourdan, Judith Harvey-White, Melanie Königshoff, Malliga R. Iyer, Resat Cinar

**Affiliations:** 1 Section on Fibrotic Disorders and; 2 Laboratory of Cardiovascular Physiology and Tissue Injury, National Institute on Alcohol Abuse and Alcoholism, NIH, Rockville, Maryland, USA.; 3 Center for Lung Aging and Regeneration, Division of Pulmonary, Allergy, Critical Care, and Sleep Medicine, University of Pittsburgh School of Medicine, Pittsburgh, Pennsylvania, USA.; 4 Geriatric Research Education and Clinical Center (GRECC) at the VA Pittsburgh Healthcare System, Pittsburgh, Pennsylvania, USA.; 5 Section on Medicinal Chemistry and; 6 Laboratory of Physiologic Studies, National Institute on Alcohol Abuse and Alcoholism, NIH, Rockville, Maryland, USA.; 7 UFR Sciences Vie Terre Environnement, Université de Bourgogne Europe, Dijon, France.; 8 INSERM Research Center U1231, Pathophysiology of Dyslipidemia team, Dijon, France.

**Keywords:** Immunology, Inflammation, Pulmonology, Drug therapy, Fibrosis, Macrophages

## Abstract

Pulmonary fibrosis (PF) is a life-threatening disease that requires effective and well-tolerated therapeutic modalities. Previously, the distinct pathogenic roles of cannabinoid receptor 1 (CB_1_R) and inducible nitric oxide synthase (iNOS) in the lungs and their joint therapeutic targeting were highlighted in PF. However, the cell-specific role of CB_1_R in PF has not been explored. Here, we demonstrate that CB_1_R in alveolar macrophages (AMs) mediates the release of anandamide into the alveoli, which promotes PF by inducing pro-fibrotic macrophages that are accessible to locally delivered antifibrotic therapy. A multitargeted therapy may improve therapeutic efficacy in PF. Pulmonary delivery of 0.5 mg/kg/d MRI-1867 (zevaquenabant), a peripherally acting hybrid CB_1_R/iNOS inhibitor, was as effective as systemic delivery of 10 mg/kg/d and also matched the efficacy of nintedanib in mitigating bleomycin-induced PF. A systems pharmacology approach revealed that zevaquenabant and nintedanib treatments reversed pathologic changes in both distinct and shared PF-related pathways, which are conserved in human and mouse. Moreover, zevaquenabant treatment also attenuated fibrosis and pro-fibrotic mediators in human precision-cut lung slices. These findings establish CB_1_R-expressing AMs as a therapeutic target and support local delivery of dual CB_1_R/iNOS inhibitor zevaquenabant by inhalation as an effective, well-tolerated, and safe strategy for PF.

## Abstract 2

Cannabinoid receptor 1 (CB1R) expressing alveolar macrophages promotes profibrotic activation of macrophages in pulmonary fibrosis. Pulmonary delivery of zevaquenabant (dual peripheral CB1R/iNOS antagonist) provided an effective and safe therapeutic modality with its clinical use potential.

## Graphical abstract

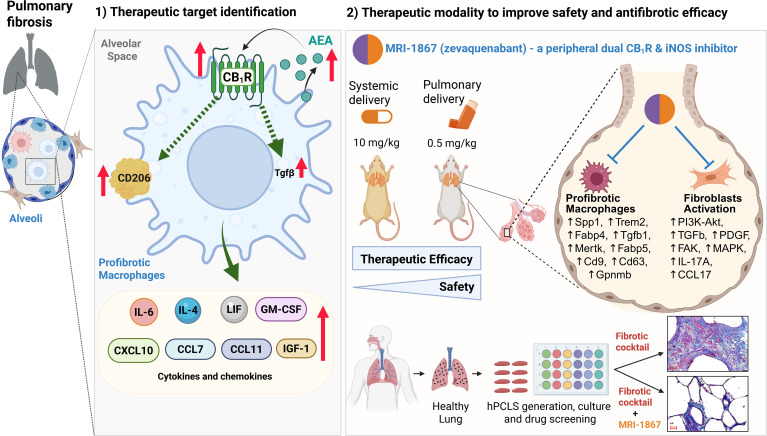

## Introduction

Pulmonary fibrosis (PF) is a life-threatening disease that requires effective therapeutic modalities. Pirfenidone and nintedanib are the only approved medications for idiopathic pulmonary fibrosis (IPF). These medications are modestly effective and have adverse effects that make them intolerable for some patients (1), leaving room for alternative therapies with improved efficacy, tolerability, and safety.

Endocannabinoids are bioactive lipid mediators that act on the same cannabinoid receptors as the psychoactive ingredient of cannabis. Overactivity of the endocannabinoid/cannabinoid receptor 1 (CB_1_R) system contributes to the development of multiple fibrosing interstitial lung diseases, such as IPF (2), Hermansky-Pudlak syndrome pulmonary fibrosis (HPSPF) (3, 4), and radiation-induced PF (5). Accordingly, CB_1_R antagonism has been identified as a therapeutic modality, as genetic deletion or pharmacological inhibition of CB_1_R prevents or attenuates PF development and progression in bleomycin-induced PF models (2, 3). CB_1_R antagonism offers proven therapeutic benefits in multiple metabolic diseases, such as obesity, diabetes, and dyslipidemia, and fibrotic disorders of the liver, heart, kidney, skin, and lung (6). Despite the proven clinical efficacy of the first-generation brain penetrant CB_1_R antagonist rimonabant in obesity and metabolic syndrome, the clinical use of CB_1_R antagonists has been abandoned because of psychiatric side effects of rimonabant that are mediated by CB_1_R in the brain (7). In the last 2 decades, multiple studies have uncovered the pathological functions of overactive CB_1_R in peripheral organs in obesity and fibrotic disorders (6, 8–11). In preclinical studies, peripherally restricted CB_1_R antagonists have provided therapeutic efficacy without causing CNS-mediated side effects, which has revitalized the translational potential of peripheral CB_1_R antagonism in metabolic disorders (6). Currently, the peripheral CB_1_R antagonist monlunabant (MRI-1891, INV-202) is reported to be advancing to the phase IIB clinical stage after successfully completing phase IB (NCT05282446) and IIA (NCT05891834) studies in obesity and metabolic syndrome (12). The phase II clinical study with monlunabant not only reinstated the clinical efficacy of peripheral CB_1_R antagonism but also highlighted the importance of optimizing CNS safety margin. Among the spectrum of fibrotic disorders, PF clinical trials have the longest duration, up to 2 to 3 years, which further underlines the importance of safety and tolerability. One effective approach to increase tolerability and safety in fibrotic lung diseases is to deliver the drug to the lungs via inhalation, which allows direct interaction with the intended target(s). Pulmonary drug delivery to the local fibrotic lung microenvironment may decrease systemic exposure. This results in an increased safety margin for the inhaled compound over its long-term use. Although CB_1_R is expressed at very low levels in the nonfibrotic lung, it is consistently and robustly upregulated across multiple cell types in the fibrotic lungs of both humans and mice, such as macrophages, pneumocytes, fibroblasts, pericytes, and endothelial cells (2). Furthermore, the level of the endogenous CB_1_R agonist anandamide (AEA) was significantly increased in bronchoalveolar lavage fluid (BALF) of patients with IPF and HPSPF compared with their respective healthy controls, and it was negatively correlated with pulmonary function test parameters (2, 3).

Like those of human PF, AEA levels in BALF were also increased in bleomycin-induced PF mouse models. The increase in the BALF was decreased by CB_1_R antagonism, which resulted in the attenuation of PF (2, 3). All of these findings point to the critical role of AEA and CB_1_R activation in the fibrotic lung microenvironment in PF. However, cell-specific roles of CB_1_R in fibrotic lungs have not yet been explored. Accordingly, we hypothesized that overactivity of the endocannabinoid/CB_1_R system in cells located in the alveoli, such as macrophages, contributes to the pro-fibrotic microenvironment. Therefore, selective targeting of CB_1_R locally in the fibrotic lung via pulmonary delivery of a peripherally acting CB_1_R antagonist could provide optimized antifibrotic efficacy while enhancing CNS safety.

Another approach for optimizing efficacy and safety is a multitarget therapeutic approach, which attenuates multiple fibrotic pathways to achieve improved clinical efficacy. Recently, orally bioavailable hybrid CB_1_R/inducible nitric oxide synthase (iNOS) antagonist zevaquenabant (MRI-1867) has been introduced as a dual-target, peripherally acting CB_1_R antagonist (6, 13, 14). Indeed, overactivity of CB_1_R and of iNOS both contribute to different forms of organ fibrosis through mutually exclusive pathways. Accordingly, use of zevaquenabant (13) provided superior antifibrotic efficacy compared with targeting either CB_1_R or iNOS antagonism alone in experimental models of liver (13), lung (2, 3), skin (15), and kidney (16) fibrosis. These studies have validated the benefits of the dual-targeting approach in fibrosis, including PF and supported the therapeutic potential of zevaquenabant, which has completed a phase I clinical trial (NCT04531150).

In the present study, we have focused on the cell-specific role of CB_1_R in alveolar macrophages (AMs) and its endogenous agonist AEA in the fibrotic lungs. The findings indicate that localized delivery of zevaquenabant by the inhalational route can reduce both drug dose and systemic drug exposure while maintaining antifibrotic efficacy in a mouse model of PF.

## Results

### AMs and alveolar type 2 epithelial cells generate and release AEA upon bleomycin-induced injury.

To date, neither the cell type responsible for releasing AEA into the alveolar space nor the cell-specific role of CB_1_R in PF has been explored. In fibrotic lungs, CB_1_R protein is mostly found in macrophages and alveolar type 2 epithelial (AT2) cells (2). Our previous finding of a significant inverse correlation between AEA in BALF and pulmonary function test parameters prompted us to identify the cellular source of AEA in the alveolar space. To explore this, we measured bleomycin-induced AEA generation and release into the culture media by AT2 cells and AMs, selected for their proximity to the alveolar space. Bleomycin (10 mU/mL) exposure of AT2 cells and AMs induced AEA release into the cell culture media (Supplemental Figure 1, A and C; supplemental material available online with this article; https://doi.org/10.1172/jci.insight.187967DS1). AMs responded more rapidly than AT2 cells to bleomycin, inducing AEA with a shorter lag time compared with AT2 cells (Supplemental Figure 1, A and C). Similarly, bleomycin-induced CB_1_R gene expression also showed a shorter lag time in AMs than in AT2 cells (Supplemental Figure 1, B and D).

### Cell-specific deletion of CB_1_R in myeloid cells but not in AT2 cells prevents the development of bleomycin-induced PF.

**Figure 1:**
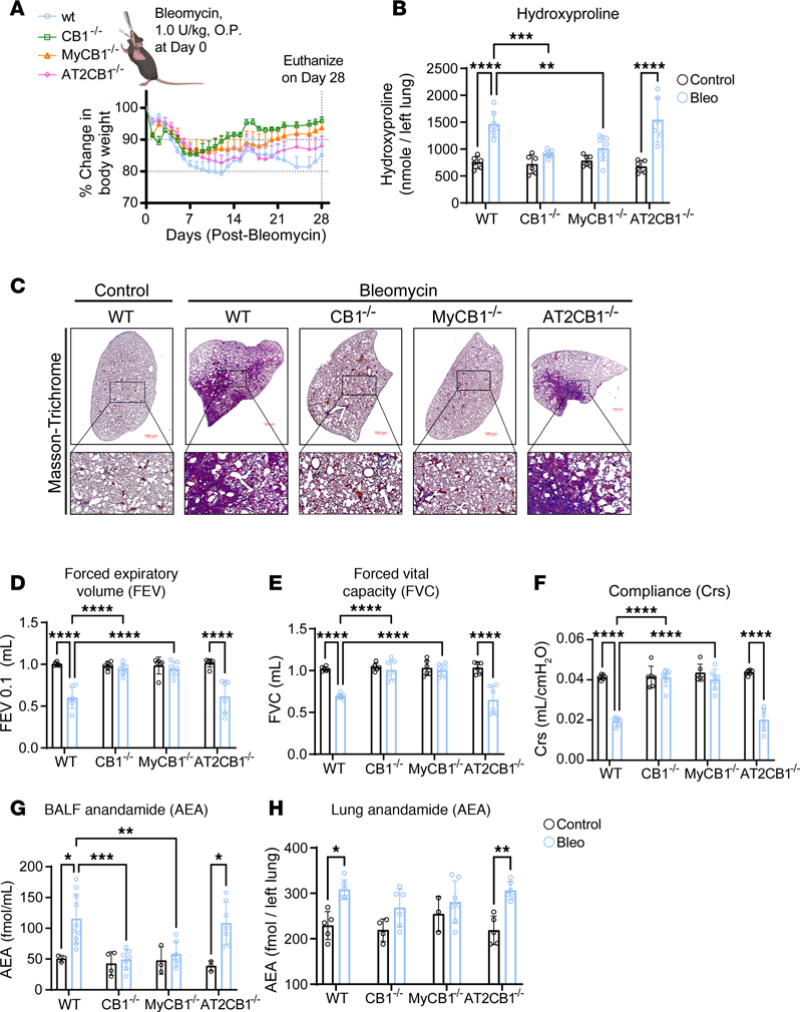
Deletion of CB_1_R in myeloid cells, but not in AT2 cells, prevents PF. (**A**) Change in percentage in body weight after bleomycin administration in different groups showing prevention of body weight loss in CB_1_R-KO and myeloid CB_1_R-KO mice but not in AT2 CB_1_R-KO mice in 28-day bleomycin-induced PF model (*n* = 7 per group). O.P., oropharyngeal. (**B**) Significant reduction of bleomycin-induced hydroxyproline content found in the CB_1_R-KO and myeloid CB_1_R-KO mice but not in AT2 CB_1_R-KO mice at 28 days postbleomycin (2-way ANOVA, ****P < 0.0001, ***P < 0.001, **P < 0.01, *n* = 6–7 per group). (**C**) Representative images of Masson’s trichrome–stained lung sections showing reduced collagen deposition and better architecture in CB_1_R-KO and myeloid CB_1_R-KO mice but not in AT2 CB_1_R-KO mice. (**D**–**F**) Retention of various pulmonary functions (FEV_0.1_, FVC, and Crs) at the normal level was found in CB_1_R-KO and myeloid CB_1_R-KO mice but not in AT2 CB_1_R-KO mice after being challenged with bleomycin (2-way ANOVA, ****P < 0.0001, ***P < 0.001, *n* = 6–7 per group). (**G** and **H**) Levels of AEA in BALF and lung were reduced in bleomycin-challenged CB_1_R-KO and myeloid CB_1_R-KO mice but not in AT2 CB_1_R-KO mice (2-way ANOVA, ***P < 0.001, **P < 0.01, *P < 0.05, *n* = 3–10 per group).

To further explore the contribution of CB_1_R overactivity to the development of PF, we generated myeloid and AT2 cell–specific CB_1_R-KO mice as described in the Methods (Supplemental Figure 2A), then evaluated them for bleomycin-induced PF at 28 days after bleomycin (Figure 1A). Importantly, deletion of CB_1_R in myeloid cells but not in AT2 cells significantly mitigated bleomycin-induced body weight reduction (Figure 1A), mortality (Supplemental Figure 3A), and PF development compared with their wild-type littermates, and the protective effect was nearly comparable to that in global CB_1_R-KO mice (Figure 1), as depicted biochemically by lung hydroxyproline content (Figure 1B) and histologically by Masson’s trichrome staining (Figure 1C). The bleomycin-induced worsening of pulmonary function parameters (forced expiratory volume [FEV], forced vital capacity [FVC], compliance, pressure-volume loops, stiffness index, and peripheral airway resistance) was also fully prevented in both myeloid CB_1_R-KO and global CB_1_R-KO mice but not in AT2 cell–specific CB_1_R-KO mice (Figure 1, D–F, and Supplemental Figure 3, B–D).

### Activation of CB_1_R in myeloid cells increases AEA levels in BALF in bleomycin-induced PF.

Since myeloid CB_1_R-KO mice were protected from bleomycin-induced PF (Figure 1, B and C), we investigated whether deletion of CB_1_R in myeloid cells prevents the elevation of AEA levels in BALF. We found that deletion of CB_1_R, either globally or in myeloid cells alone, prevented bleomycin-induced elevation of AEA in BALF and lung tissue (Figure 1, G and H), indicating the suppression of CB_1_R-mediated endocannabinoid signaling in the lungs.

### Global or myeloid deletion of CB_1_R attenuates bleomycin-induced PF.

We previously demonstrated that PF development reaches a peak at 14 days after bleomycin (17). The fibrotic lung transcriptome in mice at 14 days after bleomycin resembles the fibrotic changes in the human IPF lung transcriptome (17). As both inflammatory and fibrotic components are present at 14 days after bleomycin, we also explored the effects of CB_1_R deletion at 14 days after bleomycin. In agreement with the findings at 28 days after bleomycin in this model, deletion of CB_1_R in myeloid cells or globally prevented body weight decline (Supplemental Figure 4A), fibrosis development (Supplemental Figure 4, B and C), pulmonary function decline (Supplemental Figure 4, D–I), and mortality (Supplemental Figure 4J) at 14 days after bleomycin. These results indicate that CB_1_R activation in myeloid cells plays a crucial role in the development of PF.

### AMs expressing CB_1_R may play a role in the development of PF.

**Figure 2:**
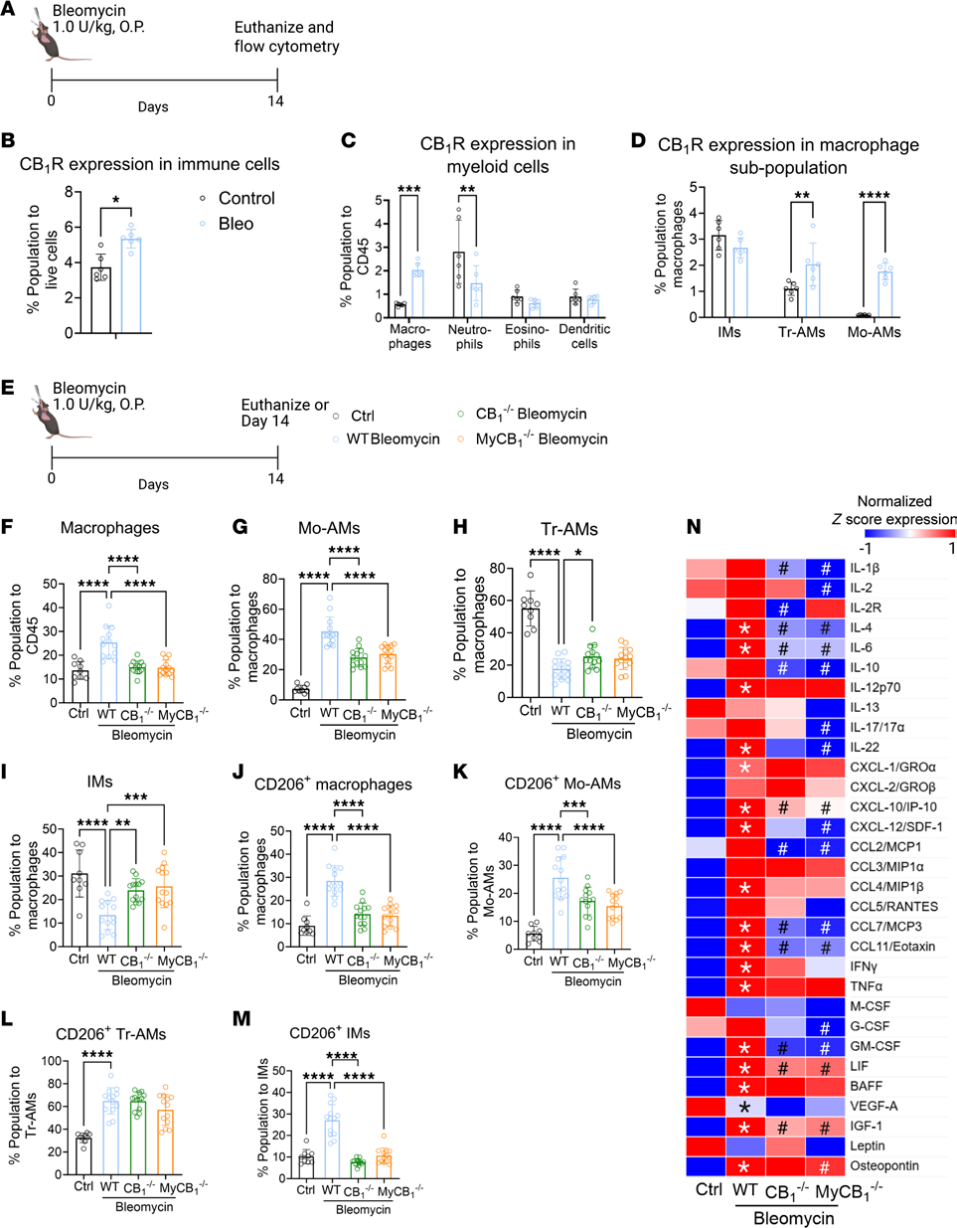
Deletion of CB_1_R in myeloid cells attenuates pro-fibrotic macrophages and microenvironment. (**A**) Bleomycin-induced PF model. (**B**) A significant increase in the CB_1_R protein expression in the lung immune cells was observed in the fibrotic mice (1-way ANOVA, *P < 0.05, *n* = 6 per group). (**C**) During lung fibrosis, CB_1_R expression increased only in the macrophages among all myeloid cells in the lungs (2-way ANOVA, ***P < 0.001, **P < 0.01, *n* = 6 per group). (**D**) Among different subsets of macrophages, CB_1_R expression only increased in tissue-resident alveolar macrophages (Tr-AMs) and monocyte-derived alveolar macrophages (Mo-AMs), indicating the significance of CB_1_R in the fibrotic alveolar microenvironment (2-way ANOVA, ****P < 0.0001, **P < 0.01, *n* = 6 per group). (**E**) CB_1_R-KO and myeloid CB_1_R-KO mice were challenged with bleomycin-induced PF model. (**F**) A reduction in the total macrophage population was found in both CB_1_R-KO and myeloid CB_1_R-KO mice (1-way ANOVA, ****P < 0.0001, *n* = 9–13 per group). (**G**–**I**) Phenotypic alterations in different subpopulations of macrophages: Mo-AMs, Tr-AMs, and IMs. Infiltration of Mo-AMs was found in the fibrotic lungs, which was reduced in both CB_1_R-KO and myeloid CB_1_R-KO mice (1-way ANOVA, ****P < 0.0001, ***P < 0.001, **P < 0.01, *P < 0.05, *n* = 9–13 per group). (**J**–**M**) The total CD206^+^ macrophages and subpopulations of CD206^+^ macrophages, Tr-AMs, Mo-AMs, and IMs, in different groups. CB_1_R deletion significantly attenuated the total CD206^+^ macrophages as well as CD206^+^ MO-AMs and IMs (1-way ANOVA, ****P < 0.0001, ***P < 0.001, *n* = 9–13 per group). (**N**) A multiplex Luminex assay was used to measure secreted cytokines in BALF. (1-way ANOVA, *P < 0.05 indicates significant difference compared with control. ^#^*P* < 0.05 indicates a significant difference compared with WT bleomycin group. *n* = 4–11 per group.)

Crossing LysM-Cre with CB_1_R-floxed mice to generate myeloid CB_1_R-KO mice (Supplemental Figure 2A) led to deletion of CB_1_R in multiple LysM-Cre–expressing myeloid cells. To identify bleomycin-induced alterations in the specific CB_1_R-expressing myeloid cell populations in PF, we conducted flow cytometry to detect CB_1_R overexpression in different myeloid subpopulations, including macrophages (tissue-resident AMs [Tr-AMs], interstitial macrophages [IMs], and monocyte-derived AMs [Mo-Ams]), neutrophils, eosinophils, and dendritic cells (DCs), using the described gating strategy (Supplemental Figure 5). Cells were isolated from mouse lungs at 14 days after bleomycin (Figure 2A), at which time both inflammatory and fibrotic components were present based on the lung transcriptome (17). We optimized the CB_1_R antibody staining for flow cytometry by using a stable CB_1_R-expressing HEK293 cell line as a positive control (Supplemental Figure 6, A–C). In fibrotic mice, bleomycin increased CB_1_R protein expression only in TR-AMs and Mo-AMs but not in other myeloid cell populations (Figure 2, B–D, and Supplemental Figure 6, D and E). Since there was a bleomycin-induced overexpression of CB_1_R only in AMs, we isolated primary AMs (pAMs) from healthy control mice (Supplemental Figure 1, E and F) and incubated them in vitro for 24 hours with bleomycin (1 mU/mL). This resulted in a significant increase in both the release of AEA to the cell culture media (Supplemental Figure 1E) and in CB_1_R gene expression in the pAMs (Supplemental Figure 1F) that replicated the findings in the AM cell line (Supplemental Figure 1, C and D). These data identify CB_1_R-expressing AMs as one of the myeloid cell types involved in pro-fibrogenic processes in PF.

### Deletion of CB_1_R in myeloid cells attenuates CD206-expressing macrophages in lungs in bleomycin-induced PF.

Bleomycin challenge increases total macrophages in fibrotic lungs (Supplemental Figure 6D), which consist of Tr-AMs, Mo-AMs, and IMs (Supplemental Figure 6E). This increase is attributed to the increased infiltration of Mo-AMs in the lungs (Supplemental Figure 6E) since the levels of Tr-AMs and IMs (Supplemental Figure 6E) were reduced rather than increased by bleomycin. Macrophages, including Mo-AMs, contribute to the progression of PF by acquiring pro-fibrotic phenotypes (18). We investigated the effect of global or myeloid deletion of CB_1_R on macrophage populations and individual phenotypes by flow cytometry using the described gating strategy (Supplemental Figure 7) at 14 days after bleomycin (Figure 2E). We found that deletion of CB_1_R in myeloid cells and globally reduced the total macrophage population (Figure 2F) and Mo-AMs (Figure 2G). Additionally, CB_1_R deletion partially reversed the bleomycin effect by reducing Tr-AMs and IMs (Figure 2, H and I). Furthermore, the increased infiltration of neutrophils induced by bleomycin was also attenuated by CB_1_R gene deletion (Supplemental Figure 8B). Macrophage activation and polarization contribute to PF development by inducing pro-inflammatory (M1, CD80^+^) and alternatively activated (M2, CD206^+^) macrophages (19). CD206^+^ macrophages are also regarded as antiinflammatory or pro-fibrotic macrophages during lung fibrosis (20). Recently, Pommerolle et al. reported that CD206^+^ macrophages are sensitive biomarkers for monitoring fibrosis severity and progression, with a strong correlation with computed tomography imaging (20). As expected, bleomycin induced both CD80^+^ and CD206^+^ macrophages in the lungs (Figure 2J and Supplemental Figure 8C). Deletion of CB_1_R either in myeloid cells or in whole body similarly attenuated levels of CD206^+^ Mo-AMs, TR-AMs, and IMs in lungs at 14 days after bleomycin (Figure 2, K–M). However, only CD80^+^ IMs but not Tr-AMs and Mo-AMs were significantly reduced by the conditional deletion of CB_1_R (Supplemental Figure 8, D–F). These results suggest that CB_1_R-expressing macrophages may regulate both infiltration and acquisition of the pro-fibrotic macrophage phenotype by Mo-AMs. We also explored the regulatory role of CB_1_R expressed by myeloid cells on the fibrogenic microenvironment by measuring secretory cytokines and chemokines in BALF. We found that pro-inflammatory and pro-fibrotic macrophage-specific cytokines and chemokines were significantly and similarly reduced in the myeloid CB_1_R-KO and global CB_1_R-KO mice compared with WT mice with PF (Figure 2N), among which the most important were IL-6, IL-4, CXCL10, CCL7, CCL11, GM-CSF, LIF, and IGF-1.

### CB_1_R antagonism attenuates bleomycin-induced increase in pro-fibrotic gene expression and CD206 protein expression in AMs.

**Figure 3:**
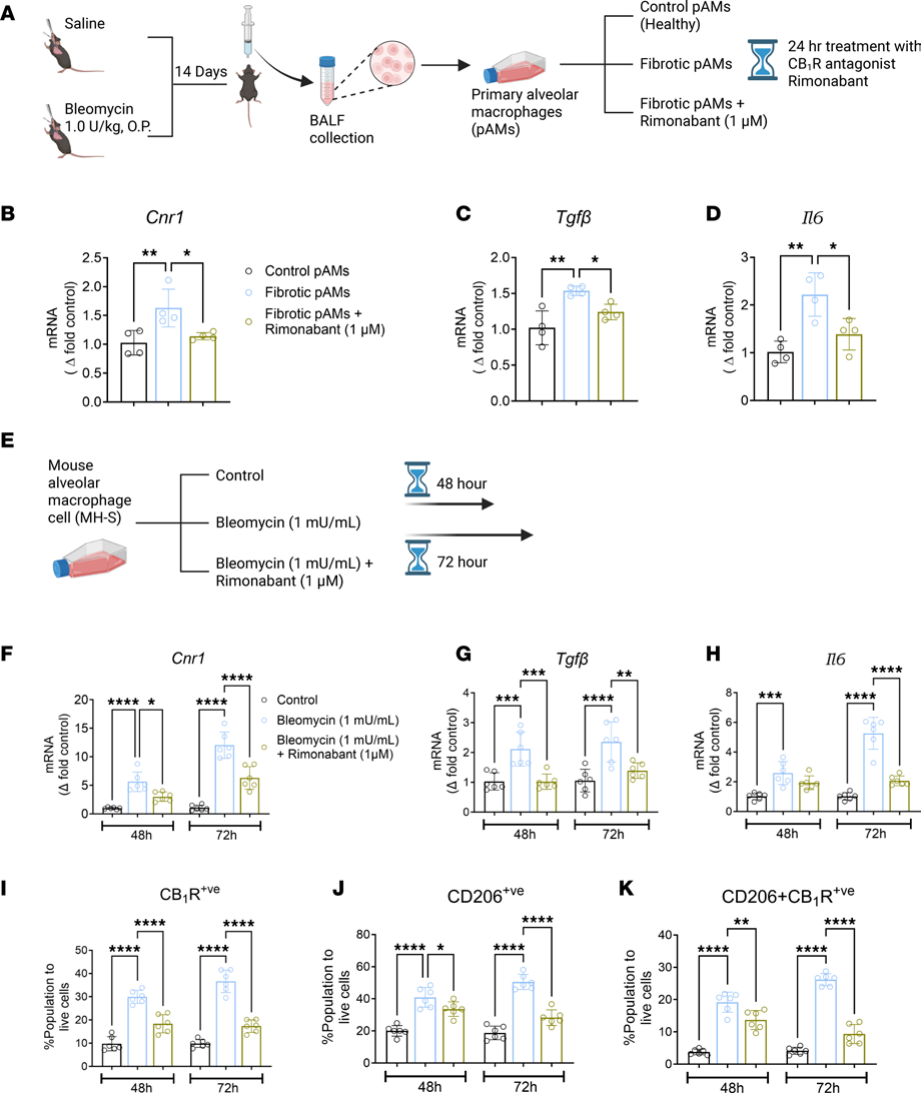
CB_1_R activation in AMs could regulate the activation of pro-fibrotic macrophages. (**A**–**D**) Primary alveolar macrophages (pAMs) were isolated from healthy mice (control) and fibrotic mice 14 days after exposure to saline and bleomycin, respectively. Gene expression of *Cnr1*, *Tgfβ*, and *Il-6* in pAMs ex vivo at 24 hours in the absence or presence of 1 μM of rimonabant (1-way ANOVA, **P < 0.01, *P < 0.05, *n* = 4 per group). (**E**–**H**) Gene expression of *Cnr1*, *Tgfβ*, and *Il-6* in MH-S cell line (mouse alveolar macrophage cell line) at 48 and 72 hours after exposure to bleomycin (1 mU/mL) in the absence or presence of 1 μM of rimonabant. Control group was treated with saline as a vehicle for bleomycin. (1-way ANOVA, ****P < 0.0001, ***P < 0.001, **P < 0.01, *P < 0.05, *n* = 6 per group.) (**I**–**K**) Cell surface protein expression of CB_1_R and CD206 and coexpression in MH-S cell line at 48 and 72 hours after exposure to bleomycin (1 mU/mL) in the absence or presence of 1 μM of rimonabant determined by flow cytometry analysis. Control group was treated with saline as a vehicle of bleomycin. (1-way ANOVA, ****P < 0.0001, ***P < 0.001, **P < 0.01, *P < 0.05, *n* = 6 per group.)

Since bleomycin triggered overactivation of CB_1_R by increasing AEA release and CB_1_R expression in AMs, next we focused on investigating the role of CB_1_R activation in AMs in promoting pro-fibrotic macrophage microenvironment in both pAMs isolated 14 days after bleomycin (Figure 3A) and mouse AM cell line (MH-S) treated with bleomycin (Figure 3E). Bleomycin-induced increased expression of CB_1_R in AMs in vivo (Figure 2, C and D) was also observed in isolated pAMs cultured ex vivo (Figure 3B). Importantly, ex vivo treatment of cells with the CB_1_R antagonist rimonabant (1 μM) attenuated bleomycin-induced *Cnr1*, *Tgfβ*, and *Il-6* expression in pAMs isolated at 14 days after bleomycin (Figure 3, A–D). Similarly, in vitro bleomycin exposure also increased gene expression of *Cnr1*, *Tgfβ*, and *Il-6* in mouse AM cell line (MH-S). These increases were significantly attenuated by rimonabant (Figure 3, E–H). Since CB_1_R-mediated pro-fibrotic gene expression was similar in pAMs and MH-S cell line, we further explored the cell surface protein expression of CB_1_R and CD206 in MH-S cells by flow cytometry. As expected, bleomycin exposure for 48 and 72 hours increased protein expression of CB_1_R (Figure 3I) and CD206 (Figure 3J), as well as the number of dual-positive AMs (Figure 3K). Importantly, rimonabant significantly diminished the increased protein expression of CB_1_R, CD206, and their dual positivity in AMs. These findings also suggest that CB_1_R-expressing AMs might be a therapeutic target for CB_1_R antagonism in the local fibrotic lung microenvironment.

### Spatial transcriptomics analysis from human fibrotic lung tissues reveals that CB_1_R gene expression is increased in pro-fibrotic macrophages.

**Figure 4:**
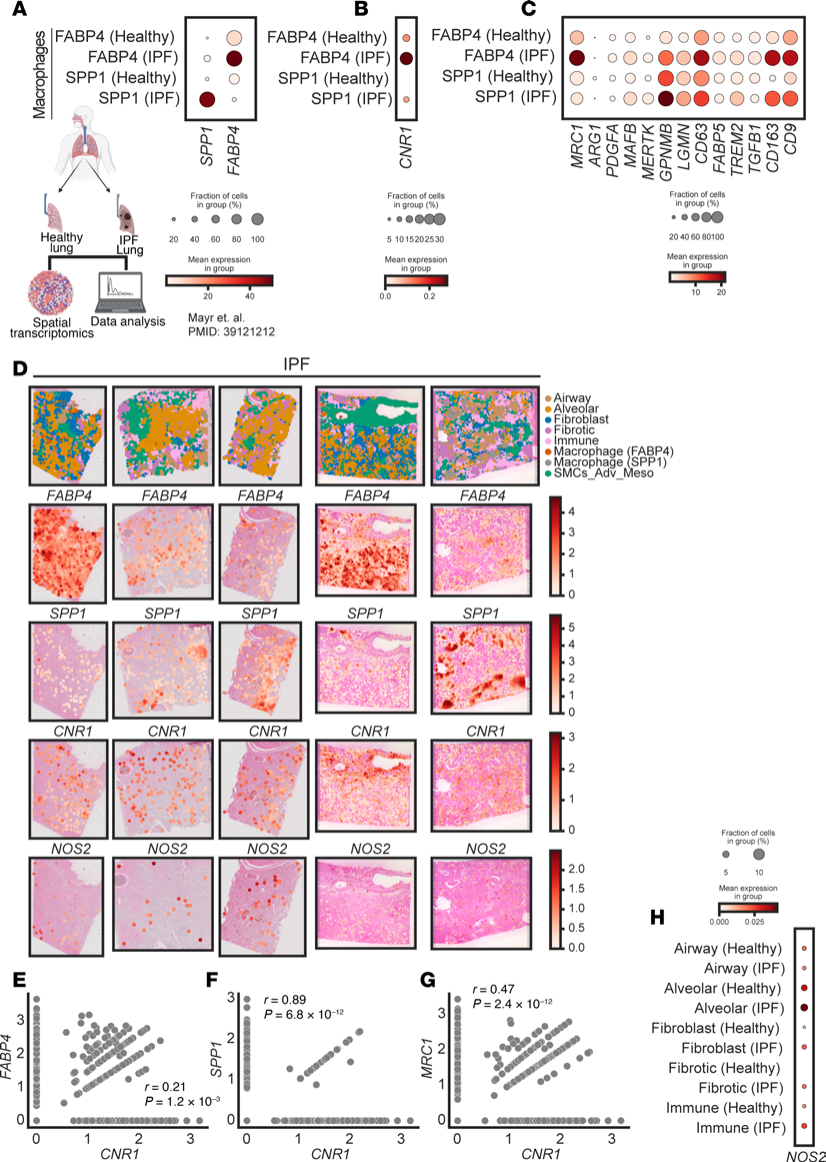
Spatial transcriptomics analysis of the human IPF lungs reveals the importance of targeting CB_1_R (*CNR1*) and iNOS (*NOS2*). (**A**) Comparative expression of pro-fibrotic macrophage population denoted by *FABP4*^+^ and *SPP1*^+^ macrophages between healthy human and IPF human lungs (25). (**B**) Expression of *CNR1* was increased in both *FABP4*^+^ and *SPP1*^+^ macrophages in the human IPF lungs. (**C**) Expression pattern of fibrogenic and/or scar-associated macrophage markers in both *FABP4*^+^ and *SPP1*^+^ macrophages in the human IPF lungs reflecting their association with *CNR1* expression. (**D**) Spatial plots visualize the niche annotation in human IPF lung samples. Spatial patterns of *FABP4*, *SPP1*, *CNR1*, and *NOS2* in human IPF lung samples. Airway: basal, ciliated; Alveolar: AT0, AT1, AT2; Fibroblast: alveolar, peribronchial, pericyte; Fibrotic: aberrant basaloid, myofibroblast, IL-1B^+^ macrophages; Immune: dendritic, B/plasma, T cell; SMCs_Adv_Meso: smooth muscle, adventitial fibroblast, mesothelial cells. (**E**–**G**) Correlation analysis between the expression of *CNR1* and key pro-fibrotic macrophage markers, *FABP4*, *SPP1*, and *MRC1*, respectively, in the IPF lungs. (**H**) Comparative expression of *NOS2* in different lung population niche between healthy human and IPF human lungs.

Recently, SPP1^+^ macrophages (21) and FABP4^+^ macrophages (21) were identified as pro-fibrotic macrophages. Furthermore, single-cell RNA-sequencing studies identified increased expression of multiple genes as markers of scar-associated macrophages or fibrogenic macrophages (21–24). Since CB_1_R may play a critical role in the regulation of pro-fibrotic macrophages (Figures 2 and 3), we analyzed the expression pattern of CB_1_R in a recently published spatial transcriptomics database of the lungs of healthy controls and patients with IPF (25) (Figure 4). We found that *CNR1* expression was increased in *FABP4^+^* and *SPP1^+^* pro-fibrotic macrophages (Figure 4, A and B), and this increase was corroborated by multiple markers of fibrotic macrophages, including *MAFB*, *MERTK*, *TREM2*, *CD163*, *CD9*, *CD63*, *GPNMB*, and *MRC1* (Figure 4C). *CNR1* expression was also positively correlated with coexpression of *SPP1*, *FABP4*, and *MRC1* in the fibrotic human lungs of patients with IPF (Figure 4, D–G), highlighting *CNR1* in fibrogenic macrophages as a promising therapeutic target.

### iNOS gene expression is increased in the fibrotic niche of lungs from patients with IPF.

In addition to the upregulation of CB_1_R, multiple studies demonstrated that overactivity of iNOS in the lungs contributes to PF, suggesting that iNOS inhibition can provide therapeutic benefit (26–29). For example, increased iNOS expression in the lungs has been associated with induced proliferation and activation of human pulmonary fibroblasts (3, 30, 31) and is positively correlated with disease severity in patients with IPF and systemic sclerosis (31, 32). We previously demonstrated that CB_1_R and iNOS are involved in PF via distinct pathogenic pathways (2, 3), and these observations suggest that CB_1_R and iNOS may be localized in different cell types in the fibrotic lung microenvironment. Here, the spatial transcriptomics analysis indicated that iNOS expression is increased in multiple cell types in the lungs of patients with IPF compared with healthy controls (Figure 4H). These cell types include fibroblasts (alveolar, peribronchial, and pericyte), immune cells (dendritic, B/plasma, and T cells), and fibrotic niche cells (aberrant basaloid, myofibroblast, and IL-1β^+^ macrophages) (Figure 4H).

### Pulmonary delivery of MRI-1867 results in good target engagement while reducing systemic exposure to the drug.

**Figure 5:**
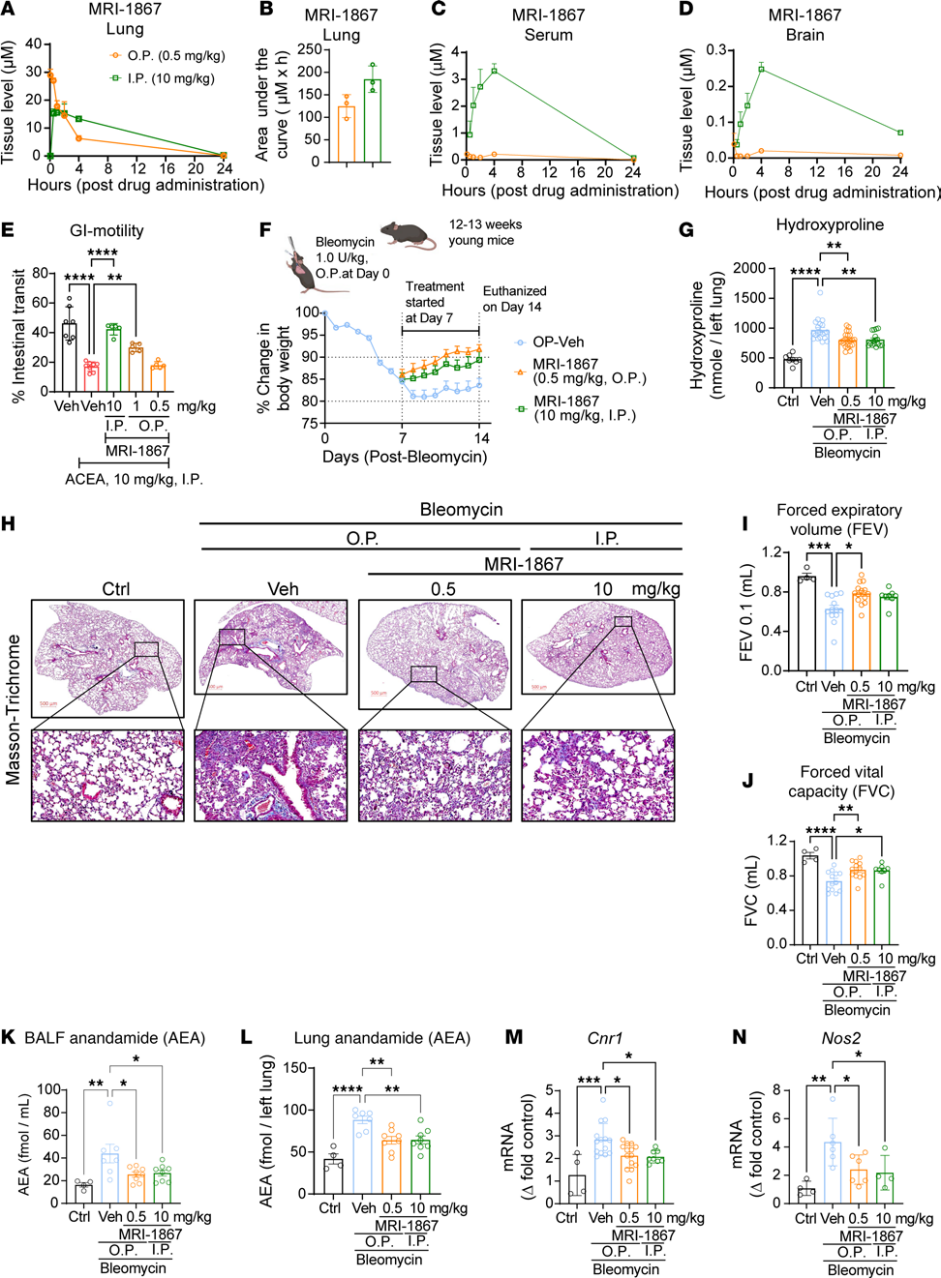
Pulmonary delivery of MRI-1867 at a reduced dose reveals antifibrotic efficacy. (**A** and **B**) Pharmacokinetic profile and area under the curve of MRI-1867 at 0.5 mg/kg b.w. O.P. and 10 mg/kg b.w. I.P. doses showing similar concentrations in the lungs over 24 hours (unpaired *t* test, *P* > 0.05, *n* = 3 per group). (**C** and **D**) Comparative exposure of MRI-1867 in the serum and brain between O.P. (0.5 mg/kg b.w.) and I.P. (10 mg/kg b.w.) doses, indicating reduced exposure via pulmonary delivery (*n* = 3 per group). (**E**) Upper gastrointestinal motility assay showing in vivo systemic peripheral CB_1_R antagonism potency of MRI-1867 (1-way ANOVA, ****P < 0.0001, **P < 0.01, *n* = 5–8 per group). (**F**) Improvement in body weight loss was found after treatment with MRI-1867 (*n* = 8–10 per group). (**G**) Attenuation of hydroxyproline content by MRI-1867 treatment showing antifibrotic efficacy (1-way ANOVA, ****P < 0.0001, **P < 0.01, *n* = 8–22 per group). (**H**) Masson’s trichrome staining of the mice lung showed a reduction in the collagen deposition and alveolar space constriction by the treatment with MRI-1867. (**I** and **J**) MRI-1867 via both O.P. and I.P. dosage showed comparable improvement in pulmonary functions as depicted by FEV and FVC (1-way ANOVA, ****P < 0.0001, ***P < 0.001, **P < 0.01, *P < 0.05, *n* = 4–14 per group). (**K** and **L**) MRI-1867 at both dosages reduced anandamide levels in the BALF and lungs (1-way ANOVA, ****P < 0.0001, **P < 0.01, *P < 0.05, *n* = 4–7 per group). (**M** and **N**) MRI-1867 at 0.5 mg/kg b.w. O.P. dose reduced the gene expression of *Cnr1* and *Nos2* at a similar degree as compared with the I.P. dose of 10 mg/kg b.w. (1-way ANOVA, ***P < 0.001, **P < 0.01, *P < 0.05, *n* = 4–14 per group.) GI, gastrointestinal.

Considering the distinct targets of CB_1_R and iNOS in PF, their simultaneous engagement would be an effective therapeutic strategy (2, 3). Indeed, oral administration of MRI-1867 to mice with experimental PF provided antifibrotic efficacy that exceeded the efficacy of targeting either CB_1_R or iNOS alone (2). Since both CB_1_R and iNOS are increased in the fibrotic alveolar microenvironment (Figure 4, B and H), their joint targeting by locally delivered MRI-1867 could be expected to retain antifibrotic therapeutic efficacy while improving CNS safety. Previously, MRI-1867 achieved complete peripheral CB_1_R antagonism in vivo through systemic administration (oral or intraperitoneal) at a 10 mg/kg dose (13). It also provided therapeutic efficacy in the bleomycin-induced PF model at the same dose with a lung exposure of ~10 μM, which completely attenuated both bleomycin-induced iNOS activity and CB_1_R activation (2, 3). To establish the pharmacokinetic/pharmacodynamic relationship and target exposure in the lungs, we aimed to achieve similar lung concentrations of ~10 μM with pulmonary delivery of MRI-1867 using the O.P. route of administration. We found that O.P. delivery of MRI-1867 at a dose of 0.5 mg/kg/d achieved the same therapeutic lung concentration of ~10 μM and area under the curve as that achieved by an intraperitoneal (I.P.) dose of 10 mg/kg/d, which yielded a C_max_ in healthy mice (Figure 5, A and B) that was sufficient for full target engagement of both CB_1_R and iNOS in the lungs (2). This was achieved despite much lower drug concentrations in serum (0.26 μM) and brain (0.032 μM), compared with the corresponding values after systemic administration (3.31 and 0.248 μM in serum and brain, respectively, Figure 5, C and D). Accordingly, 0.5 mg/kg O.P. administration of MRI-1867 did not result in systemic CB_1_R antagonism as verified using the upper gastrointestinal motility assay, unlike the systemic administration of MRI-1867 at 10 mg/kg (Figure 5E). However, the O.P. dose of 1 mg/kg MRI-1867 caused partial systemic CB_1_R antagonism (Figure 5E). Therefore, a dose of 0.5 mg/kg MRI-1867 was selected as an ideal O.P. dose for drug delivery restricted to the lungs. Additionally, exposure of different lung lobes to MRI-1867 after a single O.P. dose was similar in healthy mice (Supplemental Figure 9A), suggesting a homogeneous distribution of MRI-1867 after the O.P. delivery.

### MRI-1867 treatments at 0.5 mg/kg/d O.P. or 10 mg/kg/d I.P. provide comparable antifibrotic efficacy in bleomycin-induced PF.

Next, we compared the therapeutic efficacy of MRI-1867 administered via 2 routes. Following a single O.P. dose of bleomycin (1 U/kg), we started daily treatment with MRI-1867 at 7 days after bleomycin either by the O.P. route at 0.5 mg/kg/d or the I.P. route at 10 mg/kg/d. Daily treatment continued until day 13 after bleomycin, and mice were euthanized on day 14. In the PF model (Figure 5F), mice treated with MRI-1867 via either O.P. or I.P. route showed similar reductions in collagen deposition as quantified by hydroxyproline content (Figure 5G) and illustrated by histology (Figure 5H). Improvement in pulmonary functions including FEV, FVC, compliance, and pressure-volume loops was also similar after the 2 routes of treatment (Figure 5, I and J, and Supplemental Figure 9, D and E). Lung exposure to MRI-1867 was similar (>5 μM) after the 2 routes of administration (Supplemental Figure 9B) and resulted in similar attenuation of the bleomycin-induced increases in *Cnr1* and *Nos2* expression (Figure 5, M and N). MRI-1867 also prevented the decline in body weight (Figure 5F) and reduced mortality (Supplemental Figure 9C).

### Bleomycin-induced increase in AEA in BALF and lung tissue is similarly attenuated by oropharyngeal and systemic MRI-1867 treatments.

Previously, we reported increased AEA levels in BALF of IPF and HPSPF patients with increased expression of CB_1_R, as compared with the BALF from respective control individuals (2, 3). This trend of increased AEA and CB_1_R overexpression is replicated in the bleomycin-induced PF mouse model (2, 3). Accordingly, genetic deletion or pharmacological inhibition of CB_1_R attenuated the bleomycin-induced increase in AEA levels, demonstrating AEA’s utility as a target engagement marker in the fibrotic lung microenvironment (2). Here, we found that bleomycin-induced AEA levels in BALF and lung tissue in mice were significantly and similarly attenuated by MRI-1867 treatments via the 2 routes of administration (Figure 5, K and L).

### Modulation of immune cell populations and fibrogenic macrophages in the lungs by systemic and oropharyngeal MRI-1867 treatments in bleomycin-induced PF.

**Figure 6:**
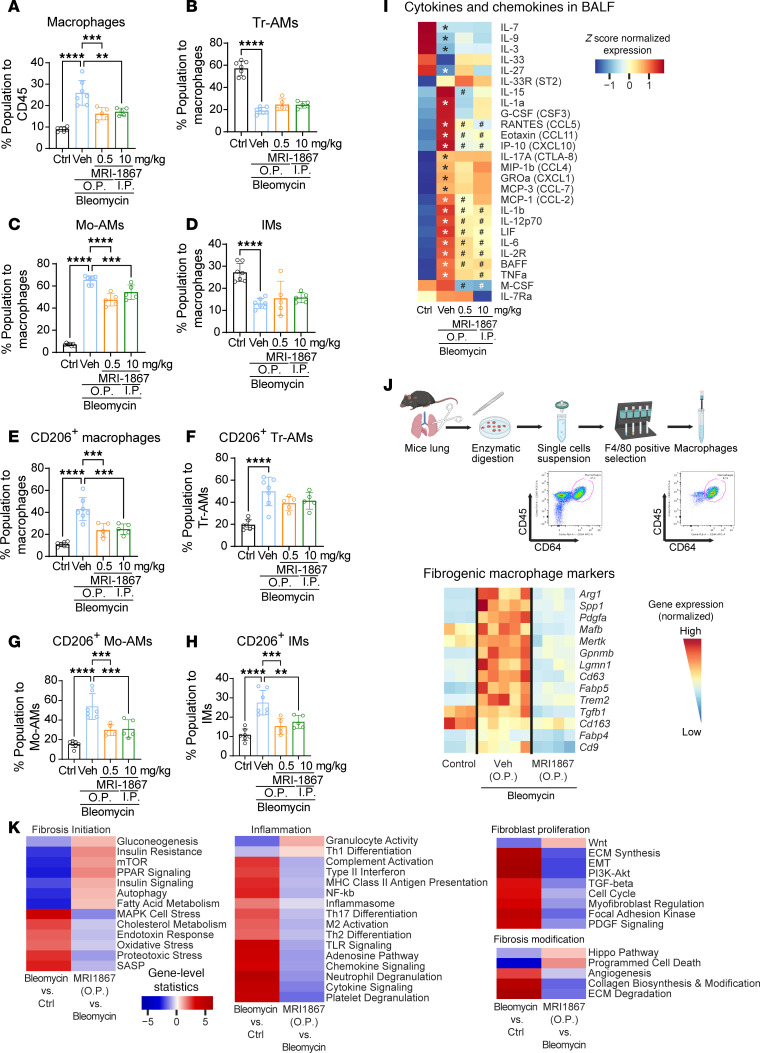
Modulation of the fibrotic microenvironment by MRI-1867. (**A**) Immunophenotyping by flow cytometry revealed a reduction in the total macrophage population by both dosages of MRI-1867. (**B**–**D**) Phenotypic alterations in different subpopulations of macrophages: tissue-resident alveolar macrophages (Tr-AMs), monocyte-derived alveolar macrophages (Mo-AMs), and interstitial macrophages (IMs). Infiltration of Mo-AMs was found in the fibrotic lungs and MRI-1867 treatment reduced this population (1-way ANOVA, ****P < 0.0001, ***P < 0.001, **P < 0.01, *n* = 5–7 per group). (**E**) MRI-1867 predominantly targets the CD206^+^ macrophages by reducing the population increase due to fibrosis (1-way ANOVA, ****P < 0.0001, ***P < 0.001, *n* = 5–7 per group). (**F**–**H**) The subpopulations of CD206^+^ macrophages, Tr-AMs, Mo-AMs, and IMs, in different groups. MRI-1867 significantly attenuated the CD206^+^ MO-AMs and IMs (1-way ANOVA, ****P < 0.0001, ***P < 0.001, **P < 0.01, *n* = 5–7 per group). (**I**) MRI-1867 ameliorates the fibrotic microenvironment by altering cytokines and chemokines in the BALF from mice. Heatmap showing modifications in the stimulatory, pro-fibrotic, and chemotactic cytokines by MRI-1867 (independent *t* test, *P < 0.05 vs. Ctrl, ^#^*P* < 0.05 vs. bleomycin-O.P. Veh, *n* = 4–10 per group). (**J**) Attenuation of fibrogenic macrophage signature by pulmonary delivery of MRI-1867 in the lungs. Heatmap is generated from the *z*-score–normalized expression (mRNA) of the macrophages isolated from the lungs of different groups (independent *t* test, *P* < 0.05, *n* = 3–5 per group). (**K**) Inhalational MRI-1867 treatment via the O.P route at 0.5 mg/kg reversed the majority of bleomycin-induced fibroproliferative pathways in the fibrotic lungs. MRI-1867 treatment regulates the pathways involved in different phases of fibrosis, thereby exerting its antifibrotic efficacy (independent *t* test, *P* < 0.05, *n* = 3 per group).

Previously, we reported that systemic CB_1_R antagonism attenuated the activation of AMs recovered from the BAL (2) and lungs (Figure 2) in bleomycin-induced PF. Here, we performed immunophenotyping by flow cytometry of lung tissue to assess the modulation of myeloid cell populations in fibrotic lungs using the gating strategy depicted in Supplemental Figure 7. MRI-1867 treatment significantly attenuated the levels of total macrophages and Mo-AMs in the lungs (Figure 6, A–D). MRI-1867 treatment significantly attenuated bleomycin-induced CD206^+^ pro-fibrotic macrophages in whole-lung macrophages, Mo-AMs, and IMs (Figure 6, E, G, and H). Additionally, MRI-1867 slightly reduced CD80^+^ macrophages in Mo-AMs and IMs, though this was not statistically significant (Supplemental Figure 10, A–E). Phenotypic characterization of lung macrophages revealed that treatment with MRI-1867 significantly reduced the pathogenic Mo-AMs (Figure 6C) and the population of fibrogenic macrophages (CD206^+^) (Figure 6, E–H).

### Both oropharyngeal and systemic MRI-1867 attenuate secretory cytokines and chemokines in BALF in bleomycin-induced PF.

The fibroproliferative milieu of the lung microenvironment is essential for the infiltration of Mo-AMs, the polarization of AMs, and the activation of fibroblasts, which all contribute to promoting fibrosis. Therefore, we explored the status of cytokines and chemokines in BALF using Luminex assay. Multiple stimulatory (BAFF, IL-2R, and IL-12p70), pro-fibrotic (IL-17A, IL-1a, IL-1b, TNFa, IL-6, and LIF), and chemotactic (RANTES, IP-10, Eotaxin, MCP-1, MIP-1b, GROa, and MCP-3) cytokines and chemokines in BALF were increased by bleomycin (Figure 6I). MRI-1867 treatment via either the O.P. or I.P. route significantly attenuated BAFF, IL-2R, IL-12p70, IL-1b, TNFa, IL-6, LIF, RANTES, IP-10, Eotaxin, MCP-1, and IL-15 (Figure 6I). Additionally, the significant downregulation of M-CSF by MRI-1867 suggests inhibition of M2 polarization.

### O.P. delivery of MRI-1867 mitigates fibrogenic macrophages in the fibrotic lungs of aged mice.

The role of macrophages in promoting fibrosis has been recognized for many years. In this study, analysis of lung immune cells revealed that the antifibrotic effect of MRI-1867 was mainly mediated by a reduction in fibrogenic macrophages and their cytokine signaling. Due to the diversity of macrophages, the downregulation of CD206^+^ macrophages did not capture the complete picture of the effectiveness of MRI-1867. Recently, various studies have identified an increased expression of multiple genes, including *Cd9*, *Trem2*, *Spp1*, *Gpnmb*, *Fabp5*, *Cd63*, *Fabp4*, *Arg1*, *Mertk*, *Lgmn1*, *Cd163*, *Tgfb1*, *Pdgfa*, and *Mafb*, as markers of scar-associated macrophages or fibrogenic macrophages (21–24). Therefore, we further investigated the effectiveness of MRI-1867 on scar-associated macrophages or fibrogenic macrophages by examining the expression of these multiple markers in lung macrophages (Figure 6J). First, we isolated macrophages from the right lower lobe of the lungs using magnetic sorting, and then we performed quantitative PCR analyses. Due to the induction of fibrosis, the expression of fibrogenic macrophage markers was significantly increased (Figure 6J) and was also significantly reduced by delivering MRI-1867 directly into the lungs. These data indicate that the substantial therapeutic effect of MRI-1867 mainly originates from its interaction with fibrogenic macrophages in the alveolar fibrotic microenvironment.

### Pulmonary targeting of CB_1_R and iNOS with MRI-1867 significantly attenuates multiple fibroproliferative pathways in the fibrotic lung.

MRI-1867 treatment via the O.P. route resulted in the same level of therapeutic efficacy (Figure 5, F–L), macrophage infiltration and polarization (Figure 6, A and E), and soluble fibroproliferative markers (Figure 6I) as systemic administration. Multiple biological pathways contribute to the pathology at different stages of PF. To assess the effect of O.P. MRI-1867 treatment on regulating the fibrogenic pathway, we performed targeted transcriptomics using an nCounter fibrosis panel (NanoString).

Targeted transcriptomics analyses of the lungs revealed that MRI-1867 treatment significantly attenuated the differentially expressed genes (DEGs) involved in fibrosis initiation and modification pathways, fibroblast proliferation, as well as inflammatory pathways in bleomycin-induced PF (Figure 6K). Among 14 pathways involved in fibrosis initiation, 13 pathways were attenuated by O.P. MRI-1867 treatment, such as the increases in MAPK cell stress, cholesterol metabolism, endotoxin response, oxidative stress, proteotoxic stress, and senescence-associated secretory phenotype and the decreases in autophagy, insulin resistance, gluconeogenesis, mTOR, PPAR signaling, insulin signaling, and fatty acid metabolism. Among 19 inflammatory pathways, 16 pathways were attenuated by MRI-1867 treatment, such as the increases in adenosine pathway, chemokine signaling, complement activation, cytokine signaling, M2 activation, neutrophil degranulation, NF-κB, platelet degranulation, Th17 differentiation, TLR signaling, type II interferon, Th2 differentiation, MHC class II antigen presentation, and inflammasome and the decreases in granulocyte activity and Th1 differentiation. Among 12 fibroblast proliferation pathways, 9 pathways were attenuated by MRI-1867 treatment, such as the increases in cell cycle, ECM synthesis, epithelial-mesenchymal transition, focal adhesion kinase, myofibroblast regulation, PDGF signaling, PI3K-AKT, and TGF-β signaling and the decrease in the Wnt pathway. Among 6 fibrosis modification pathways, 5 pathways were attenuated by MRI-1867 treatment, such as the increases in collagen biosynthesis and modification, ECM degradation, and angiogenesis, as well as the decreases in apoptosis and the hippo pathway. These results demonstrate that O.P. MRI-1867 treatment reversed fibrosis-induced alterations in nearly all fibrogenic pathways (Figure 6K).

### Dual inhibition of CB_1_R and iNOS by MRI-1867 provides improved antifibrotic efficacy compared with targeting CB_1_R or iNOS alone in aged mice.

**Figure 7:**
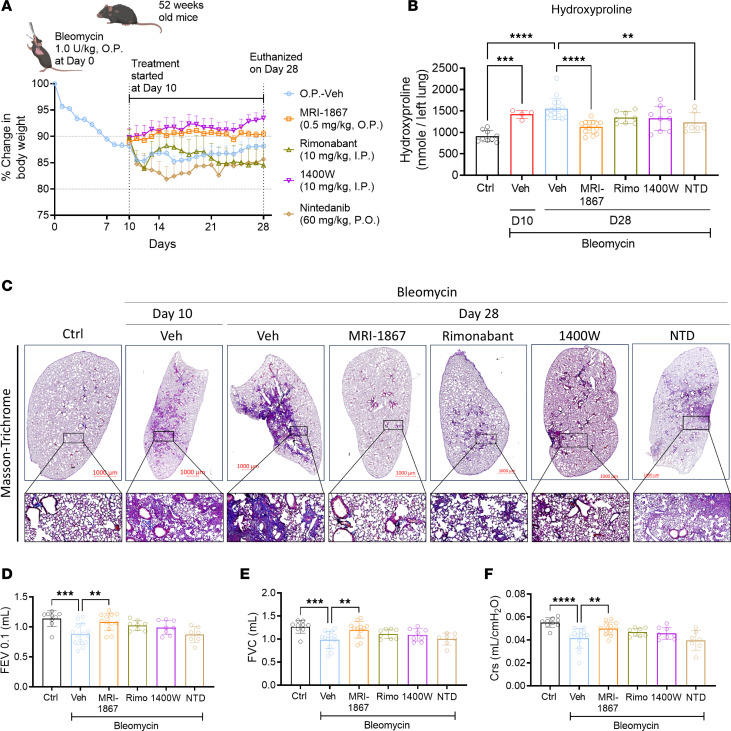
Dual inhibition of CB_1_R and iNOS by MRI-1867 provides superior antifibrotic efficacy in aged mice. (**A**) Improvement in body weight loss was found after treatment with MRI-1867 via the O.P. route in 52-week-old (aged) mice. All the treatments were started on day 10, then continued up to day 28, and mice were euthanized on day 28 after bleomycin (1 U/kg b.w., O.P., *n* = 8–16 per group). (**B**) Bleomycin-induced elevated hydroxyproline levels were only significantly reduced by dual targeting of CB_1_R and iNOS with MRI-1867 and nintedanib in the 28-day bleomycin-induced PF model in 52-week-old mice (1-way ANOVA, ****P < 0.0001, **P < 0.01, *n* = 8–13 per group). (**C**) Masson’s trichrome staining of the mouse lungs showed a reduction in the collagen deposition and alveolar space constriction by the treatment with MRI-1867, rimonabant, 1400W, and nintedanib but better architecture found in MRI-1867 treatment compared with rimonabant, 1400W, and nintedanib. (**D**–**F**) Pulmonary delivery of MRI-1867 showed significant improvement in pulmonary functions as depicted by FEV_0.1_, FVC, and Crs (1-way ANOVA, ****P < 0.0001, ***P < 0.001, **P < 0.01, *n* = 8–14 per group). Nintedanib could not improve the pulmonary functions in these mice.

Previously, we demonstrated that fibrosis-related alterations in the lung transcriptome and metabolome peaked at 14 days after bleomycin and then remained unchanged at 21 and 28 days after bleomycin (17). Although we showed the therapeutic efficacy of MRI-1867 between days 7 and 14 after bleomycin, which may represent the early onset of the disease (Figure 5, F–L), an ideal pharmacological intervention should be effective between days 10 and 28 postbleomycin to assess antifibrotic efficacy at disease onset. Furthermore, PF is a disease that occurs primarily in the aged population. Therefore, we performed another efficacy study in aged (1-year-old) mice by starting treatment between days 10 and 28 after bleomycin (Figure 7A). In this study, we also included maximally effective doses of the prototypic CB_1_R antagonist rimonabant (10 mg/kg, I.P.) and iNOS inhibitor 1400W (10 mg/kg, I.P.) (2). Systemic administration of rimonabant or 1400W alone provided moderate antifibrotic efficacy (Figure 7, B and C), prevented mortality (Supplemental Figure 11A), as well as delayed the decline in pulmonary function parameters such as FEV, FVC, compliance (Figure 7, D–F), and pressure-volume loops (Supplemental Figure 11B). Importantly, daily O.P. administration of MRI-1867 (0.5 mg/kg) between days 10 and 28 after bleomycin surpassed the antifibrotic efficacy of targeting CB_1_R or iNOS alone (Figure 7), which aligns with our previous findings (2, 3). Furthermore, the lung transcriptomics analysis revealed that O.P. MRI-1867 treatment reversed 3,030 fibrosis-related DEGs in the lungs (Supplemental Figure 12A). Among those genes, 287 genes were selectively regulated by iNOS inhibition, since they were significantly reversed by either MRI-1867 or 1400W treatment (Supplemental Figure 12A), whereas 473 genes were selectively regulated by CB_1_R antagonism, since they were significantly reversed by either MRI-1867 or rimonabant (Supplemental Figure 12A). Approximately, 1,360 genes were jointly regulated by MRI-1867, rimonabant, and 1400W, indicating shared pathways (Supplemental Figure 12A). Overall, as expected, dual inhibition of CB_1_R and iNOS by MRI-1867 resulted in the modulation of more fibrosis-related DEGs and pathways than inhibiting CB_1_R or iNOS alone (Supplemental Figure 12, A and B).

### Both nintedanib and MRI-1867 significantly attenuate bleomycin-induced PF via reversing alterations in shared and distinct PF-dependent pathways.

**Figure 8:**
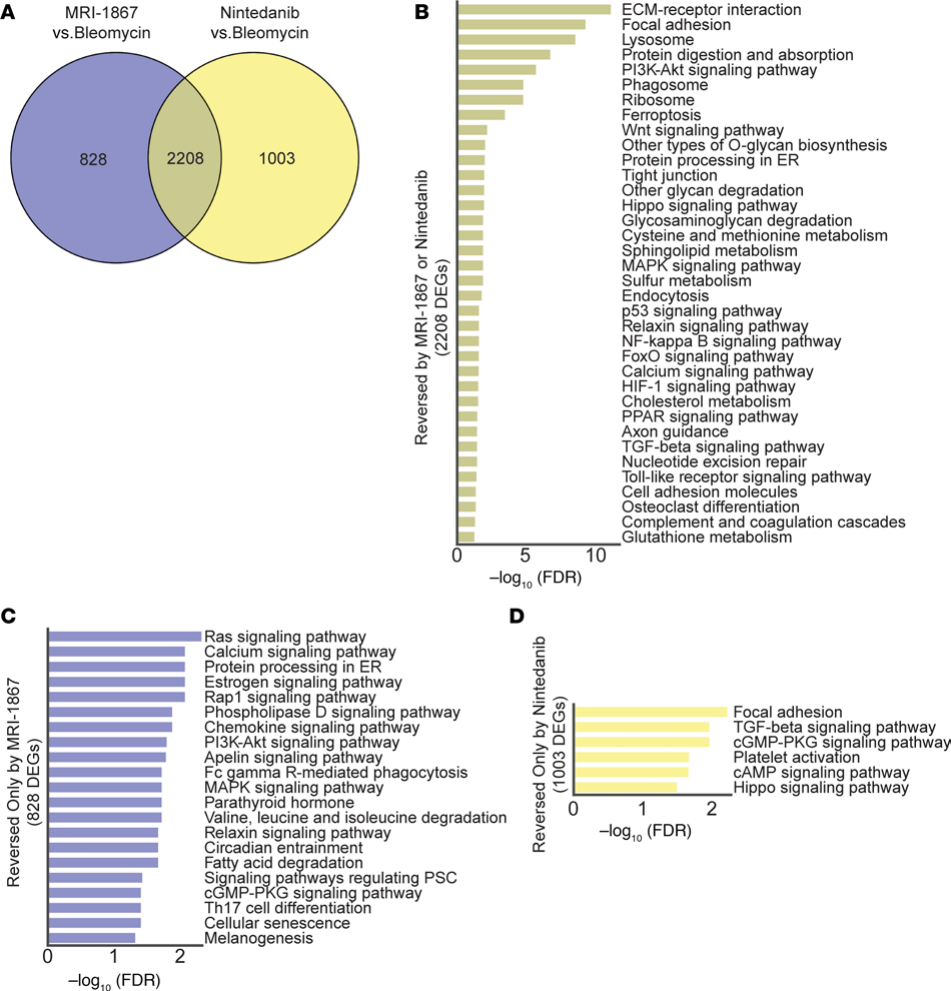
Transcriptomics analysis reveals the shared and unique effects of MRI-1867 and nintedanib treatments on attenuating multiple fibrosis-related genes and pathways in bleomycin-induced PF. (**A**) Venn diagram demonstrates numbers of DEGs that were reversed by either MRI-1867 (0.5 mg/kg, O.P.) or nintedanib (60 mg/kg, P.O.) treatment compared with the vehicle-treated group in bleomycin-induced PF in mice. (**B**) Significantly reversed fibrosis-related pathways (FDR < 0.05) based on 2,208 DEGs by either MRI-1867 or nintedanib treatment. (**C**) Significantly reversed fibrosis-related pathways (FDR < 0.05) based on 828 DEGs by only MRI-1867 but not nintedanib treatment. (**D**) Significantly reversed fibrosis-related pathways (FDR < 0.05) based on 1,003 DEGs by only nintedanib but not MRI-1867 treatment.

Nintedanib is a clinically approved antifibrotic medication for IPF and progressive PF. Therefore, we compared the therapeutic efficacy of MRI-1867 (0.5 mg/kg, administered O.P.) and nintedanib (60 mg/kg, administered via oral gavage) in bleomycin-induced PF, with treatment starting between days 10 and 28 after bleomycin in 52-week-old mice (Figure 7). Both MRI-1867 and nintedanib treatments attenuated PF as quantified by hydroxyproline (Figure 7B) and histology (Figure 7C). However, despite their similar efficacy attenuating fibrosis, only MRI-1867 treatment restored PF-induced decline in pulmonary function parameters such as FEV (Figure 7D), FVC (Figure 7E), compliance (Figure 7F), and pressure-volume loop (Supplemental Figure 11B). Unlike the CB_1_R antagonists MRI-1867 and rimonabant, nintedanib treatment did not abolish fibrosis-induced elevation of endocannabinoids in lungs (Supplemental Figure 11, C and D). These findings suggest that distinct antifibrotic mechanisms may exist between MRI-1867 and nintedanib. In mouse lung transcriptome, both MRI-1867 and nintedanib treatments significantly attenuated the same 2,208 DEGs (Figure 8A and Supplemental Data 1), and related 36 pathways, including ECM-receptor interaction, focal adhesion, protein digestion and absorption, and PI3K-AKT signaling (Figure 8B). Additionally, MRI-1867 treatment uniquely reversed PF-dependent alterations in 828 DEGs and related 21 pathways, including Ras signaling, calcium signaling, and protein processing in ER (Figure 8, A and C). Nintedanib treatment uniquely reversed PF-dependent alterations in 1,003 DEGs and related 6 pathways, including focal adhesion, TGF-β signaling, and cGMP-PKG signaling (Figure 8, A and D).

### Reversal by MRI-1867 and nintedanib of PF-dependent alterations in lung transcriptome that are shared in mouse and human.

**Figure 9:**
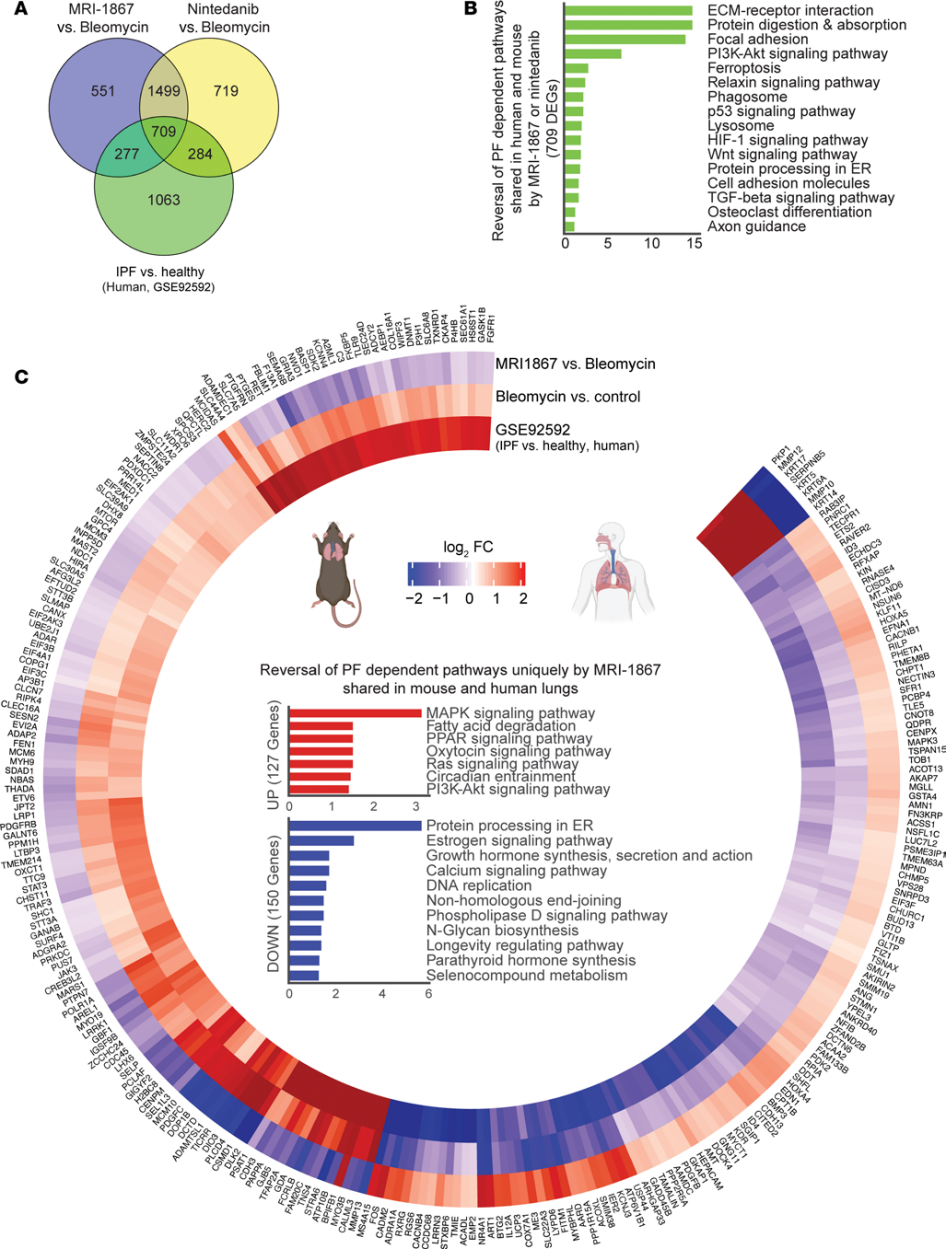
Translationally relevant genes and pathways reversed by either MRI-1867 or nintedanib treatments. (**A**) Venn diagram demonstrates numbers of fibrosis-related DEGs in lung transcriptome that were reversed by either MRI-1867 (0.5 mg/kg, O.P.) or nintedanib (60 mg/kg, P.O.) treatment compared with the vehicle-treated group in bleomycin-induced PF in mice and those shared in IPF patients’ lung transcriptome (NCBI GEO GSE92592, 39 samples) (65). (**B**) Significantly reversed fibrosis-related pathways shared in human and mouse lung transcriptome (FDR < 0.05) based on 709 DEGs by either MRI-1867 or nintedanib treatment. (**C**) Circos plot exhibiting the uniquely reversed effects of bleomycin by pulmonary delivery of MRI-1867 treatment but not by nintedanib treatment compared with human late-stage IPF patients’ lung transcriptomics data (GSE92592, 39 samples) (65) and the reversed PF-dependent biological pathways. (DESeq2, FDR < 0.05.)

Previously, we reported the translational relevance of the bleomycin-induced PF mouse model to the human disease by demonstrating the similarity of the fibrotic lung transcriptomes (17). Here, we found that O.P. treatment with MRI-1867 reversed the fibrosis-related alterations in 986 genes, which are shared between human and mouse PF (Figure 9A and Supplemental Data 1). Among these, 709 DEGs were also reversed by nintedanib, which are involved in pathways including ECM-receptor interaction, protein digestion and absorption, focal adhesion, and PI3K-AKT signaling (Figure 9B). Additionally, 277 DEGs were reversed only by MRI-1867, and these are involved in MAPK signaling, fatty acid degradation, PPAR signaling, protein processing in the ER, and estrogen signaling (Figure 9C and Supplemental Data 1). On the other hand, nintedanib treatment uniquely reversed PF-dependent alterations in 284 DEGs and the related pathways, including cGMP-PKG signaling, cAMP signaling, and platelet activation (Supplemental Figure 13 and Supplemental Data 1).

### MRI-1867 reduces fibrosis in human precision-cut lung slices by attenuating the fibroproliferative microenvironment and fibroblast activation.

**Figure 10:**
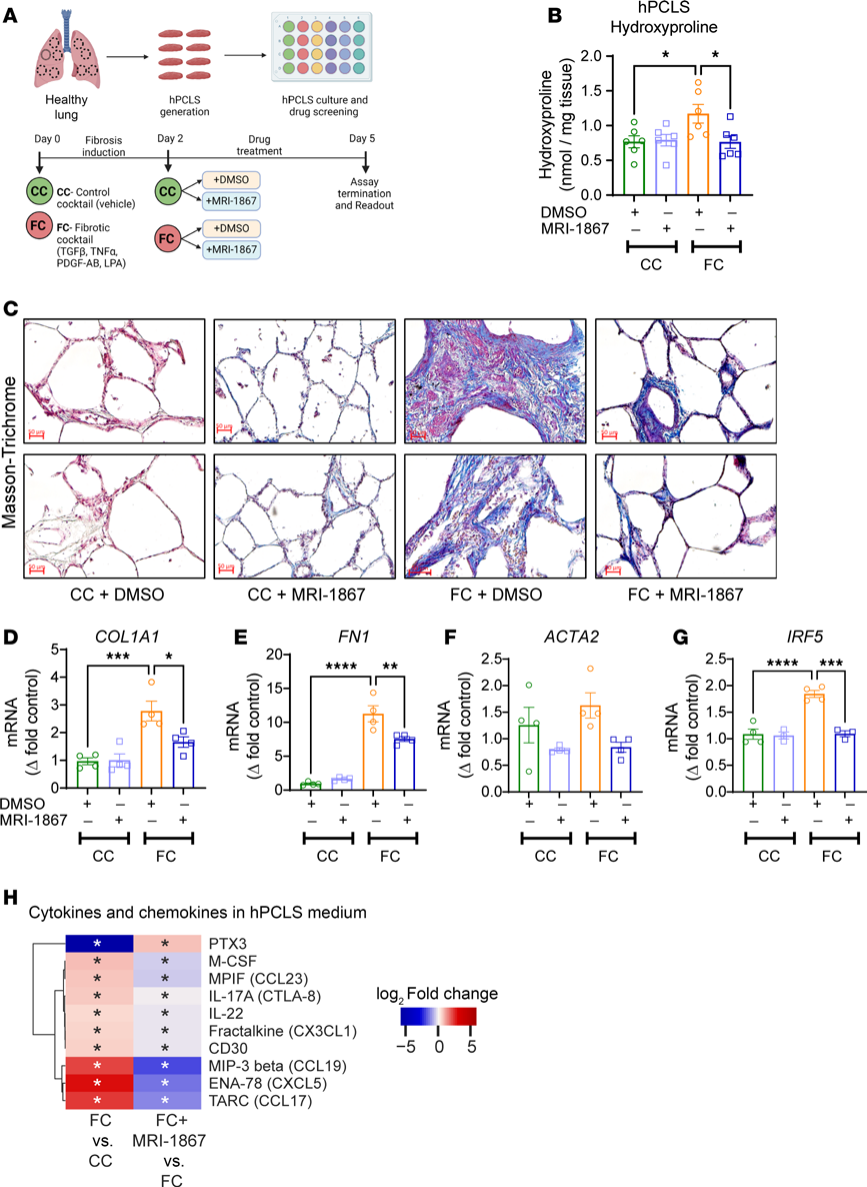
MRI-1867 attenuates fibrosis in hPCLS. (**A**) Experimental plan with hPCLS, an ex vivo model exhibiting the characteristics of IPF. Schematic of treatment with a control cocktail or fibrotic cocktail containing TGF-β. Vehicle (DMSO) and MRI-1867 (10 μM) were treated 2 days after the fibrosis induction. TGF-β, transforming growth factor-β; TNF-α, tumor necrosis factor-α; PDGF-AB, platelet-derived growth factor-AB; LPA, lysophosphatidic acid. (**B**) Reduction of collagen deposition by MRI-1867 as measured by hydroxyproline content in hPCLS (1-way ANOVA, *P < 0.05, *n* = 6 per group). (**C**) Masson’s trichrome staining shows characteristics of alveolar septal thickening and collagen fiber formation, which was markedly reduced by MRI-1867 treatment. (**D**–**G**) MRI-1867 treatment significantly reduced the gene expression of *COL1A1*, *FN1*, *ACTA2*, and *IRF5* (1-way ANOVA, ****P < 0.0001, ***P < 0.001, **P < 0.01, *P < 0.05, *n* = 4 per group). (**H**) Regulation of pro-fibrotic cytokines by MRI-1867 in fibrotic hPCLS culture supernatant (independent *t* test, FDR < 0.1. *n* = 6 per group).

To further investigate the therapeutic potential of MRI-1867 treatment in human IPF, we used human precision-cut lung slices (hPCLS) (Figure 10A). Fibrosis was induced in hPCLS by incubation with a pro-fibrotic cocktail (33) (containing TGF-β, TNF-α, PDGF-AB, and LPA) for 5 days (Figure 10A). MRI-1867 (10 μM) treatment between days 2 and 5 after fibrosis induction attenuated fibrosis in the hPCLS as quantified biochemically by hydroxyproline (Figure 10B) and depicted histologically by Masson’s trichrome staining (Figure 10C). MRI-1867 also attenuated gene expression of fibrosis and fibroblast activation markers, such as *COL1A1*, *FN1*, and *ACTA2* (Figure 10, D–F). The fibrogenic process in the hPCLS was accompanied by either increases or decreases in the release of multiple fibroproliferative chemokines and cytokines as measured from the culture medium at the end of the experiment by Luminex assay (Figure 10H). MRI-1867 treatment significantly attenuated the release of M-CSF, CCL23, IL17A, IL-22, CX3CL1, CD30, CCL19, CXCL5, and CCL17 and increased the release of PTX3 in the hPCLS (Figure 10H). IRF5 is a crucial downstream mediator of CB_1_R signaling and acts as a critical transcription factor for many pathogenic genes involved in the bleomycin-induced PF model (17). Here we found an upregulation of *IRF5* (Figure 10G), and a trend toward an increase in AEA and 2-arachidonoyl glycerol (2-AG) (Supplemental Figure 14, A and B), in fibrotic cocktail–treated hPCLS. MRI-1867 significantly downregulated the *IRF5* expression (Figure 10G), denoting the importance of CB_1_R inhibition in the fibrogenic process in human lungs. Moreover, similar levels of MRI-1867 and its metabolites M506 and M339 (34) were also observed in the hPCLS in both control and fibrotic conditions (Supplemental Figure 14, C–E), suggesting that biotransformation/metabolism of MRI-1867 by the lungs is unaffected by fibrosis.

## Discussion

Identifying effective antifibrotic therapies is needed to improve outcomes in PF. In this study, we identified CB_1_R-expressing AMs as a therapeutic target in PF by demonstrating the role of CB_1_R in pro-fibrotic macrophage activation and the induction of a fibrotic microenvironment. Furthermore, we demonstrated the prognostic utility of the endocannabinoid AEA in BALF during PF, which is increased by activation of CB_1_R in AMs. Additionally, we proposed pulmonary delivery of a third-generation CB_1_R antagonist (a dual peripheral CB_1_R/iNOS antagonist) as an effective and safe therapeutic modality in PF using the bleomycin-induced PF model. Subsequently, we demonstrated that dual targeting of CB_1_R and iNOS by MRI-1867 (zevaquenabant) via the O.P. route (0.5 mg/kg) provided similar antifibrotic efficacy as systemic administration of a 20-fold higher dose (10 mg/kg). Thus, the direct pulmonary delivery of the drug enables a reduction of its effective therapeutic dose, which could further ensure its systemic and CNS safety.

This study also demonstrated that MRI-1867 and nintedanib achieve significant antifibrotic efficacy via attenuating both distinct and shared fibroproliferative pathways and differentially altered genes in PF, which are conserved in human IPF lungs. Furthermore, MRI-1867 treatment also attenuated fibrosis and fibrotic mediators in human PCLS (Figure 10). This makes MRI-1867 an emerging candidate for prospective clinical trials in PF.

AEA detected in BALF from patients with IPF and HPSPF was previously shown to be a progressive biomarker of the development of PF (2, 3). Here, we found that deletion of CB_1_R in myeloid cells attenuated not only the AEA increase but also fibrosis, indicating the pathogenic role of CB_1_R activation in AMs. Increased AEA-mediated CB_1_R activation was also reported to contribute to other inflammatory and fibrotic disorders, such as skin fibrosis (35), kidney injury and fibrosis (36), liver cirrhosis (37), and diabetic cardiomyopathy (38). Notably, in this study CB_1_R antagonism by MRI-1867 attenuated the fibrosis-induced rise in AEA levels in BALF and lungs in mice. This further highlights the potential value of AEA as a biomarker of the fibrotic lung microenvironment (4, 39).

In addition to AEA, the other major endocannabinoid, 2-AG, was also reported to increase in various inflammatory and fibrotic conditions in skin (15), kidney (40), liver (13), and heart (41, 42), along with the overactivity of CB_1_R. Although we did not observe increased levels of 2-AG in the BALF of patients with IPF or HPSPF (2, 3), 2-AG levels were increased in the lungs and BALF of mice with bleomycin-induced PF (Supplemental Figure 3, E and F). Interestingly, deletion of CB_1_R in myeloid cells or AT2 cells had no effect on reversing the increase in 2-AG levels, but global deletion of CB_1_R completely reversed the increased abundance of 2-AG. In addition, global deletion of CB_1_R resulted in a substantially higher weight recovery than was observed in myeloid CB_1_R-KO mice. Overall, these observations suggest that a CB_1_R-expressing cell type other than AT2 cells or myeloid cells may contribute to the 2-AG increase in the fibrotic lungs and in other forms of tissue fibrosis. Indeed, this aligns with a recent finding that TGF-β treatment significantly increased CB_1_R gene expression and 2-AG but not AEA release in the lung fibrosis of patients with HPSPF (3). Also, treatment with CB_1_R antagonists attenuated the activation of fibroblasts isolated from fibrotic HPSPF lungs removed at transplantation (3). These findings suggest that the endocannabinoids AEA and 2-AG might play similar pro-fibrotic roles but in different forms of lung and tissue fibrosis. One of the limitations of this study is that we have not yet explored the roles that CB_1_R expressed in other lung cells such as fibroblasts or other myeloid cells may play in lung fibrosis, which warrant further studies.

Correction of the fibroproliferative microenvironment in fibrotic lungs by therapeutic interventions is an important factor in slowing or halting the progression of the disease. Bleomycin increases multiple fibroproliferative chemokines and cytokines, such as CCL2, CCL5, CXCL10, CCL11, BAFF, IL-12p70, IL-2R, IL-1b, TNFa, IL-6, and LIF, as we have documented in mouse BALF and as has been observed in BALF from patients with IPF (43–50). In the mouse model of PF, MRI-1867 treatment attenuated the bleomycin-induced increase in these fibroproliferative chemokines and cytokines in BALF. Moreover, MRI-1867 treatment also attenuated fibrosis and fibrosis-promoting cytokines and chemokines in human PCLS. Previous experimental and clinical studies have identified that increased levels of IL17A, M-CSF, CCL17, CCL19, CX3CL1, and CXCL5 contribute to PF development by promoting myofibroblast activation either directly or via recruiting pro-fibrotic immune cells (43, 51–57). Genetic deletion or pharmacologic inhibition/neutralization of these cytokines and chemokines, such as IL-17, M-CSF, CCL17, and CX3CL1, attenuated PF in experimental models of PF (43, 58–60). Since MRI-1867 treatment attenuated the release of these fibrosis-promoting soluble mediators in human PCLS, these findings represent a translational link to the potential therapeutic benefit of MRI-1867 in prospective clinical trials in human PF and uncover the potential mechanism of action of MRI-1867.

Previously, it was shown that Mo-AMs contribute to the development of PF (18). Deletion of CB_1_R in myeloid cells or O.P. administration of MRI-1867 not only reduced the infiltration of Mo-AMs but also attenuated pro-fibrotic macrophage activation in the lungs. This aligns with our previous observation that deletion of CB_1_R reduced the number of CD206-expressing alternatively activated macrophages (2), suggesting that CB_1_R mediates the emergence of pro-fibrotic macrophages. Previously, macrophages expressing *SPP1*, *TREM2*, and *FABP4* were identified as pro-fibrotic macrophages in single-cell RNA-sequencing studies of lung macrophages from patients with PF and in experimental models of fibrosis (61). SPP1 protein expression has also been identified as a progressive marker for disease severity and decline in pulmonary function in PF (62, 63). Additionally, increased SPP1 protein levels in BALF were found to be a predictive marker for post–COVID-19 interstitial lung disease (64). We previously demonstrated that the increase in *Spp1* gene expression is associated with pulmonary function decline in bleomycin-induced PF (17). Accordingly, the deletion of CB_1_R attenuated the fibrosis-induced expression of *Spp1* in lung transcriptomes (17). Here, we showed that oropharyngeal MRI-1867 treatment also attenuated *Spp1* expression along with other fibrotic macrophage markers, such as *Trem2*, *Fabp4*, and *Mertk*, in macrophages. A recent study showed that IL-6 and M-CSF are important activators of SPP1, disease severity, and pulmonary function decline in PF (63). O.P. administration of MRI-1867 attenuated bleomycin-induced IL-6 and M-CSF in BALF, suggesting that attenuation of pro-fibrotic macrophages is one of the targets through which MRI-1867 normalizes the fibroproliferative microenvironment. Either genetic deletion of CB_1_R in myeloid cells in mice or selective CB_1_R inhibition in AMs ex vivo attenuated the pro-fibrotic macrophage phenotype and IL-6 levels. This suggests that IL-6 and other pro-fibrotic cytokines and chemokines, such as LIF, CXCL11, and CXCL10, might be regulated by CB_1_R in macrophages.

A multitargeted therapeutic approach using either polypharmacy or polypharmacology may have a better chance of attenuating the majority of the pathogenic fibroproliferative changes. In this study, we demonstrated an example of polypharmacology using O.P. MRI-1867 treatment significantly attenuated more fibrosis-induced DEGs than did inhibition of CB_1_R or iNOS alone (Supplemental Figure 12). Altered expression of DEGs is also detectable in human IPF patients’ lung transcriptome. Consistently, dual-target inhibition of CB_1_R and iNOS provided superior efficacy compared with the single-target inhibition of CB_1_R or iNOS, as was also evident from the lung transcriptomics analysis that documented both CB_1_R- and iNOS-dependent effects of MRI-1867. Thus, oropharyngeal MRI-1867 treatment attenuated most of the pathologically altered biological pathways.

Another approach to finding therapeutic modalities is polypharmacy with combination treatment involving existing therapeutic molecules. With this in mind, we have analyzed the pro-fibrotic pathways and genes targeted by the standard-of-care therapies in PF, such as nintedanib. A comparative efficacy study presented here with MRI-1867 and nintedanib identified both shared and distinct mechanisms of action between MRI-1867 and nintedanib. Although the current study did not test the in vivo antifibrotic efficacy of a combination therapy using MRI-1867 and nintedanib, the results of lung transcriptome analyses suggest a potential additive therapeutic benefit of such a combination, which remains to be explored by future studies.

Overall, the present findings represent an important step toward the clinical development of MRI-1867 as an antifibrotic agent. Based on the present findings, an inhalation formulation of MRI-1867 is awaiting clinical validation as an emerging therapeutic modality for PF.

## Methods

### Sex as a biological variable.

IPF is seen predominantly in men. Although it could affect women, we examined only male mice to minimize use of animals in the experimental model.

### Material and reagents.

All the reagents used in this manuscript were recorded in the Supplemental Table 1. This study did not generate new unique reagents.

### Statistics.

Statistical analysis of pharmacokinetics data, pulmonary function tests, levels of hydroxyproline and anandamide, phenotypic analysis from flow cytometry, and Luminex were performed using GraphPad Prism V 9.0.0 (GraphPad Software). First, the normality test was performed to determine the distribution pattern. Then, either 1-way ANOVA or 2-way ANOVA followed by Tukey’s multiple comparisons test was performed. The nCounter (NanoString) gene expression and pathway data were analyzed using independent 2-tailed *t* test analysis from SciPy v1.7.3 package in Python 3.7. Expression values of the nCounter, Luminex, and transcripts per million of late-stage IPF and healthy human lung were *z*-score–normalized using the *z-score* function from the same package. RNA-sequencing data were analyzed using the method described previously (17), and the statistical analysis results from the nCounter (NanoString) were overlaid to the nodes to observe the effect of the treatment. *P* < 0.05 was considered statistically significant.

### Study approval.

All animal procedures were conducted in accordance with the rules and regulations of the Institutional Animal Care and Use Committee of the NIH National Institutes of Alcohol Abuse and Alcoholism under the protocols of LPS-GK1. Human lung tissue for precision-cut lung slice assay was procured by the Thoracic Tissue Repository from the University of Pittsburgh Medical Center and the Center for Organ Recovery and Education under the protocol of IRB PRO14010265 approved by the University of Pittsburgh IRB.

### Data availability.

Values for all data points in graphs are included in the Supporting Data Values file and code repository. The mouse transcriptomics data are available to download freely from GEO using the accession number GSE273132 (https://www.ncbi.nlm.nih.gov/geo/query/acc.cgi?acc=GSE273132). The codes used for data curation, analysis, and visualization are available at https://github.com/muharif/OP_MRI (commit ID 958871e). Additional details are provided in the Supplemental Methods.

## Author contributions

Conceptualization was done by RC; data curation was done by AB, MA, KMW, and RC; formal analysis was done by AB, MA, KMW, and RC; investigation was done by AB, MA, KMW, MB, CNZ, NJ, LP, JHW, MRI, TJ, SD, SBK, NJC, JKP, RHP, MK, and RC; methodology was developed by MA, AB, KMW, LP, MB, GG, RHP, JS, MK, and RC; project administration was done by RC; software was developed by MA, AB, and RC; supervision was done by RC; visualization was done by MA, AB, and RC; writing of the original draft was done by RC, AB, and MA; review and editing were done by AB, MA, KMW, MB, CNZ, NJ, LP, JHW, RHP, GG, SD, SBK, NJC, JKP, TJ, MK, MRI, RC; and funding was acquired by RC.

## Supplementary Material

Supplemental data

Supplemental data set 1

Supporting data values

## Version 1 07/03/2025

In-Press Preview

## Version 2 08/08/2025

Electronic publication
