# Supplementary material for: Targeting cannabinoid receptor 1 for antagonism in pro-fibrotic alveolar macrophages mitigates pulmonary fibrosis
Source: JCI Insight. 2025 Jul 3;10(15):e187967. doi: 10.1172/jci.insight.187967 (PMC12333952; doi:10.1172/jci.insight.187967)
Supplement: Supplemental data [file jciinsight-10-187967-s033.pdf]

Targeting cannabinoid receptor 1 for antagonism in profibrotic alveolar macrophages mitigates pulmonary fibrosis

Abhishek Basu[1#], Muhammad Arif[1,2#], Kaelin M. Wolf[1#], Madeline Behee[1], Natalie Johnson[1], Lenny Pommerolle[1], Ricardo H. Pineda[3,4], John Sembrat[3,4], Charles N. Zawatsky[1], Szabolcs Dvorácskó[1,5], Nathan J Coffey[6], Joshua K Park[6], Seray B. Karagoz[1], Grzegorz Godlewski[6], Tony Jourdan[7,8], Judith Harvey-White[1], Melanie Königshoff[3,4], Malliga R. Iyer[5], and Resat Cinar[1]*

[1] Section on Fibrotic Disorders, National Institute on Alcohol Abuse and Alcoholism, National Institutes of Health, Rockville, MD, 20852, USA.

[2] Laboratory of Cardiovascular Physiology and Tissue Injury, National Institute on Alcohol Abuse and Alcoholism, National Institutes of Health, Rockville, MD, 20852, USA.

[3] Center for Lung Aging and Regeneration, Division of Pulmonary, Allergy, Critical Care, and Sleep Medicine, University of Pittsburgh School of Medicine, Pittsburgh, PA, USA.

[4] Geriatric Research Education and Clinical Center (GRECC) at the VA Pittsburgh Healthcare System, Pittsburgh, PA, USA.

[5] Section on Medicinal Chemistry, National Institute on Alcohol Abuse and Alcoholism, National Institutes of Health, Rockville, MD, 20852, USA.

[6] Laboratory of Physiologic Studies, National Institute on Alcohol Abuse and Alcoholism, National Institutes of Health, Rockville, MD, 20852, USA.

[7] UFR Sciences Vie Terre Environnement, Université de Bourgogne Europe, 21000 Dijon, France.

[8] INSERM Research Center U1231, PAthophysiology of DYSlipidemia team, 21000 Dijon, France.

# Authors equally contributed

*Correspondence: Resat Cinar, BPharm, PhD, MBA

5625 Fishers Lane 2S-28, Rockville, MD 20852, USA

Phone: +1 (301) 443-4098

e-mail: resat.cinar@nih.gov

Keywords: Anandamide, CB_1R, iNOS, Pulmonary Fibrosis, Bleomycin, Pulmonary Function, Human precision-cut lung slices, Macrophages, SPP1, CD206, profibrotic macrophages

Supplemental Materials and Methods

Cell line

Mouse alveolar macrophage cell line, MH-S was cultured in ATCC RPMI-1640 medium with 0.05 mM mercaptoethanol, 90% along with fetal bovine serum, 10% at 37°C in a 5% CO_2 incubator. MH-S cells were exposed to 1 mU/mL of bleomycin for 72 h. The cells and the culture supernatant were collected at 48 h, and 72 h. Rat alveolar type 2 cell line, RLE-6TN was cultured in Ham's F12 medium with 2 mM L-glutamine supplemented with 0.01 mg/mL bovine pituitary extract, 0.005 mg/mL insulin, 2.5 ng/mL insulin-like growth factor, 0.00125 mg/mL transferrin, and 2.5 ng/mL EGF, 90% along with fetal bovine serum, 10% at 37°C in a 5% CO_2 incubator. Rat alveolar macrophage cell line, NR8383 [AgC11x3A, NR8383.1] was cultured in Ham's F12K medium with 2 mM L-glutamine adjusted to contain 1.5 g/L sodium bicarbonate, 85%; heat-inactivated fetal bovine serum, 15% at 37°C in a 5% CO_2 incubator. Both these cell lines were exposed to 10 mU/mL of bleomycin for 72 h. The cells and the culture supernatant were collected at 24 h, 48 h, and 72 h.

Mouse primary alveolar macrophage culture

Mouse primary alveolar macrophages (pAMs) were isolated and cultured according to the protocol as described (1). Briefly, the BALF was collected from both the healthy and fibrotic mice (14 days post-bleomycin 1.0 U/kg b.w., O.P.), and 1 x 10^6 BAL cells were plated in 6-well plate with culture medium containing RPMI 1640 (no glutamine), 1x GlutaMAX, 1x Pyruvate, 1x Penicillin/Streptomycin, 20 ng/ml recombinant GM-CSF, and 10% FBS at 37°C in a 5% CO_2 incubator. After 6 hours of attachment to the plate, the

supernatant containing other types of cells is discarded. pAMs were isolated from healthy mice served as Control pAMs. pAMs isolated from fibrotic mice were divided into two groups – Fibrotic pAMs (vehicle treatment) and Fibrotic pAMs + Rimonabant, 1 μM (CB_1R antagonism) to study the effect of CB_1R antagonism in these fibrotic alveolar macrophages *ex vivo*. We also investigated the effect of bleomycin (1mU/ml) with 24h exposure in healthy control pAMs *ex vivo*.

Mice

Twelve- to 13-week-old and 52-week-old male C57BL/6J and LysM-Cre+ mice were obtained from The Jackson Laboratory (Bar Harbor, ME, USA). $CB1^{-/-}$ (CB_1R KO) mice, $MyCB1^{-/-}$ (LysM Cre-CB_1R Flox/Flox) mice, and $AT2CB1^{-/-}$ (Sftpc Cre ERT-CB_1R Flox/Flox) mice were generated on a C57Bl/6J genetic background as described in Supplemental Figure 2A (2, 3). All the mice were housed under a 12-hr light/dark cycle and fed a standard diet, *ad libitum* (Teklad NIH-31; Envigo, Huntingdon, UK).

Bleomycin-induced pulmonary fibrosis

We generated a bleomycin-induced pulmonary fibrosis model by delivering a single dose of bleomycin (1U/kg b.w.) via oropharyngeal aspiration as previously described (4). Briefly, mice were anesthetized with intraperitoneal (I.P.) injection of Ketamine/Xylazine (80 + 4mg/mL, respectively, 1 mL/kg) and placed on a surgery board in the supine position at an angle degree of 45°, with the head immobilized by an elastic band across the upper incisors. Using sterile forceps, the mouth is nudged open, and the tongue is gently pulled out toward the mandible and lower incisors. This maneuver allowed visualization of the

vocal cords under adequate lighting. Bleomycin was delivered to anesthetized mice through the oropharynx using a sterile 100 µL pipette during inspiration at a volume of 100 µL/50g body weight (b.w.). Sterile saline was used as a vehicle and applied to the control groups. The animals are then allowed to recover from the anesthesia. We have used both post-bleomycin 14 days (peak fibrosis) and 28 days (established fibrosis) timepoints for the current study. During the entire study, the body weight of each mouse every day and morbidity and mortality were recorded. Despite using LysM Cre(-/-)CB_1R flox/flox mice as a WT littermate controls for myeloid CB_1R KO (LysM-Cre (+/-)CB_1Rflox/flox) mice (Supplemental Figure 2A), we also assessed the effect of LysM-Cre expression alone in bleomycin induced PF comparing C57BL/6J and LysM-Cre (-/+) mice that bleomycin induced of similarly in both stain of mice (Supplemental Figure 2, B-G).

Administration of Drugs

In the young mice (12 weeks) fibrosis model, MRI-1867 was administered to the bleomycin-treated mice from Day 7 to Day 13, every day via O.P. and I.P. routes at the dose of 0.5 mg/kg and 10 mg/kg b.w., respectively. For O.P. delivery the mice were anesthetized using isoflurane and delivered as mentioned above for bleomycin at a volume of 50 µL/50 g b.w. The dosage of MRI-1867 was formulated with 5% DMSO + 5% Tween 80 + 90% sterile saline for I.P. injection. The dosage of MRI-1867 was formulated with 0.1 % DMSO + 1% Tween 80 + 89.9% sterile saline was administered via O.P. route to the bleomycin-treated mice and the same formulation without MRI-1867 served as O.P. Vehicle (non-treated) group in this study. In the old mice (52 weeks) fibrosis model, all the treatment was started on Day 10 and continued up to Day 28. MRI-1867 at 0.5 mg/kg

b.w., O.P. was administered as discussed above. The disease control group, O.P. Vehicle was administered similarly. Rimonabant (CB_1R antagonist) and 1400W (iNOS inhibitor) were administered via the I.P. route at 10 mg/kg b.w. Nintedanib (NTD) was also administered at 60 mg/kg b.w., per oral from Day 10 to Day 28.

Pharmacokinetic study of MRI-1867

The bioavailability of MRI-1867 was determined in the lungs, serum, and brain after 5 min, 30 min, 1 h, 2 h, 4 h, and 24 h of administration by both O.P. and I.P. routes at the dose of 0.5 mg/kg and 10 mg/kg b.w., respectively. To study the chronic accumulation, MRI-1867 was administered (both O.P. and I.P.) each day for up to 7 days (total therapeutic window in this study) and then quantified in the above-stated organs in both normal and fibrotic mice. MRI-1867 levels were measured by LC-MS/MS as described previously (5). The amounts of MRI-1867 in the samples were determined against standard curves using $^{13}C_6$-MRI-1867 as the internal standard (6).

Upper gastrointestinal motility assay

Generally, the peripheral in vivo CB_1R antagonism was tested using a functional assay to quantify CB_1R-mediated changes in upper gastrointestinal motility as previously described (7). To assess in vivo CB_1R antagonism by MRI-1867 via different routes and different doses, MRI-1867 was administered at a dose of 0.5 and 1.0 mg/kg b.w. O.P. and 10 mg/kg b.w., I.P., 30 min after an intraperitoneal dose of the CB_1R agonist arachidonyl-2′-chloroethylamide hydrate (ACEA, 10 mg/kg, I.P.) to male 10- to 12-week-old mice 1 h prior to an oral bolus of 10% charcoal suspension in 5% gum arabic. Thirty

minutes later, the mice were euthanized, and the distance traveled by the head of the marker between the pylorus and the cecum was measured and expressed as a percentage of the total length of the small intestine.

Pulmonary function tests

Respiratory system mechanics measurements were performed using the FlexiVent FX system (SCIREQ Inc., Montreal, Canada) as previously described (8). FlexiVent FX system is equipped with an FX1 module and negative pressure forced expiration extension for mice. FlexiWare v7.2 software was used to operate the system. Forced oscillation techniques and forced expiration measurements were conducted as described previously (9, 10). Pulmonary function measurements were performed at the end of the study as a terminal procedure. Mice were anesthetized by intraperitoneal (I.P.) injection of Ketamine/Xylazine, then an 18-gauge metal cannula was inserted into the trachea by small incision. Pancuronium was then administered by I.P. injection (0.8 mg/kg b.w.) to induce paralysis before connecting mice to FlexiVent and starting ventilation. Forced expiratory volume per 0.1 seconds ($FEV_{0.1}$), forced vital capacity, compliance (Crs), tissue stiffness (H), and peripheral airway resistance (G) parameters were measured. Mouse tissue was collected after performing lung function tests.

Masson's Trichrome staining

The right upper lobe was used for histology. During lung harvesting right upper lobe was excised and fixed by using neutral buffered formalin and embedded with paraffin. Paraffin-embedded tissues were sectioned (5 μm) onto glass slides by Microtome. Histological

staining was performed using Masson's Trichrome Kit (Epredia™ Richard-Allan Scientific™) with a slight optimization of the supplier's microwave staining protocol. 5 µm tissue sections were stained with the following time adjustments: Distilled water rinse adjusted to 5 min after Bouin's Fluid; Weigert's Iron Hematoxylin stain adjusted to 3 min; Biebrich Scarlet-Acid Fuchsin solution adjusted to 1 min; and Aniline Blue Solution adjusted to 10 min. All other steps were performed as instructed. Histological images were taken by Axio Imager M2 (Zeiss) using ZEN 3.1 (blue edition) software.

Hydroxyproline measurements by LC-MS/MS

Pulmonary fibrosis was quantified by measuring hydroxyproline (Hyp) content of left lung using LC-MS/MS as described previously (11). Briefly, left lung tissue was homogenized in 600 µL of ice-cold 0.1 N perchloric acid (PCA). Two hundred µL of the lung homogenate is used for endocannabinoid measurements described in below method. One mL 12 N HCl was added to the remaining 400 µL lung homogenate and the homogenate was hydrolyzed at 100°C for 4 h. Hydrolyzed samples were vortexed and centrifuged at 10,000 g for 10 min, and 5 µL hydrolysate was diluted 200-fold by the addition of 990 µL of 0.1 N PCA and 5 µL of L-Proline-$^{13}C_5$,^{15}N as internal standard. Liquid chromatography-tandem mass spectrometry (LC-MS/MS) analyses were conducted on an Agilent 6470 triple quadrupole mass spectrometer (Agilent Technologies) coupled to an Agilent 1200 LC system. 4-Hydroxyproline was separated using an Intrada Amino Acid column, 50 × 3 mm, 3 µm (Imtakt) at 40°C. Mobile phases consisted of acetonitrile/tetrahydrofuran/25 mM ammonium formate/formic acid = 9:75:16:0.3 (v/v/v/v) (phase A) and acetonitrile/100 mM ammonium formate = 20:80 (v/v) (phase B). Gradient elution (600 µL/min) was

initiated and held at 0% B for 3 min, followed by a linear increase to 17% B by 6.5 min. This was followed by a step increase to 100% B, which was held until 10 min after the gradient had begun, and then by a linear decrease to 0% B by 11 min, which was held until 13 min after the gradient had begun. The mass spectrometer was set for electrospray ionization operated in positive ion mode. The source parameters were as follows: capillary voltage, 4,000 V; gas temperature, 330°C; and drying gas, 8 L/min. Nitrogen was used as the nebulizing gas. Collision-induced dissociation (CID) was conducted using nitrogen. Hydroxyproline level was analyzed by multiple reaction monitoring. L-Proline-$^{13}C_5$,^{15}N (Sigma, cat#608114) was used as the internal standard. The molecular ion and fragments for hydroxyproline were measured as follows: m/z 132.1→86 and 132.1→68 (CID energy: 8 V and 20 V, respectively). Lung levels of hydroxyproline were determined against a standard curve, using *trans*-4-hydroxy-L-proline as standard (Sigma-Aldrich). Values are expressed as nmol/mg wet tissue.

Endocannabinoids measurement from lungs and bronchoalveolar lavage fluid by LC-MS/MS

The left lung homogenate (200 μL) described in the hydroxyproline measurement section was used and transferred in 0.5 mL of ice-cold methanol/Tris buffer (50 mM, pH 8.0), 1:1, containing 7 ng of [$^2H_4$] arachidonoyl ethanolamide ([$^2H_4$] AEA) and 50 ng of [$^2H_5$] arachidonoyl glycerol ([$^2H_5$] 2AG) as internal standard. Homogenates were extracted two times with 2 ml of CHCl3:MeOH (2:1, vol/vol). Lower chloroform phase was collected and transferred to another glass tube. Then combined chloroform phases were dried under nitrogen flow. The samples were reconstituted in 50 μL of ice-cold methanol after

precipitating proteins with ice-cold acetone prior to loading autosampler for mass spectrometry measurements. Three hundred μL of BALF was incubated at -20°C for 10 min with 900 μl ice-cold acetone and 400 μL Tris buffer (50 mM, pH 8.0) to precipitate proteins. After spinning at 3000g at 4°C for 10 min, the supernatant was transferred to a glass tube to evaporate the acetone phase under nitrogen flow. Then 0.5 mL of ice-cold methanol/Tris buffer (50 mM, pH 8.0), 1:1, containing internal standard was added to each tube. Then, it was extracted two times with 2 ml of CHCl3:MeOH (2:1, vol/vol). Lower chloroform phase was collected and transferred to another glass tube. Then combined chloroform phases were dried under nitrogen flow. The dried samples were reconstituted in 50 μL of ice-cold methanol prior to loading autosampler for LC-MS/MS mass spectrometry measurements. LC-MS/MS analyses were conducted on an Agilent 6470 triple quadrupole mass spectrometer (Agilent Technologies) coupled to an Agilent 1200 LC system. Liquid chromatographic separation was obtained using 2 μl injections of samples onto a InfinityLab Poroshell 120 EC-C18 column (3.0mm×100 mm, 2.7 Micron) from the Agilent Technologies. The autosampler temperature was set at 4°C and the column was maintained at 34°C during the analysis. Gradient elution mobile phases consisted of 0.1% formic acid in H_2O (phase A) and 0.1% formic acid in MeOH (phase B). Gradient elution (350 μL/min) was initiated at 10% B, followed by a linear increase to 50% B at 0.5 min, followed by a linear increase to 85% B at 3 min and maintained until 14 min, then increased linearly to 100% B at 18 min and maintained until 20 min. The mass spectrometer was set for electrospray ionization operated in positive ion mode. The source parameters were as follows: capillary voltage, 3,500 V; gas temperature, 300°C; drying gas, 5 L/min; nitrogen was used as the nebulizing gas. CID was performed using

nitrogen. Levels of each compound were analyzed by multiple reaction monitoring. The molecular ion and fragment for each compound were measured as follows: m/z 348.3→62.1 for AEA, m/z 379.3→287.2 for 2AG, m/z 384.3→91.1 for [$^2H_5$] 2AG, m/z 352.3→66.1 for [$^2H_4$] AEA, The Analytes were quantified using MassHunter Workstation LC/QQQ Acquisition and MassHunter Workstation Quantitative Analysis software (Agilent Technologies). Levels of AEA and 2AG in the samples were measured against standard curves.

Isolation of macrophages from mice lungs and qPCR

The right lower lobes of the aged bleomycin-induced 28-day fibrotic mice were removed, minced (tissue pieces at 1–2 mm^2), and transferred for mild enzymatic digestion for 30 min at 37°C in an enzymatic mix containing dispase (50 caseinolytic U/mL), collagenase type I (2 mg/mL), elastase (1 mg/mL), and DNase (30 µg/mL) (12). Single cells were harvested by straining the digested tissue suspension through a 70-micron mesh. After centrifugation at 300 g for 5 min, single cells were taken up in 1 mL of PBS, counted, and proceeded for dead cell removal by Dead Cell Removal Kit (Miltenyi Biotec) according to the manufacturer’s protocol. The viable cells after magnetic separation were incubated with anti-F4/80 microbeads for 15 min and positive selection by the magnetic MS columns, the macrophages were eluted. The macrophage isolation was also validated by flow cytometry (Figure 5J). The RNA from these macrophages was isolated by RNeasy Plus Mini Kit (Qiagen) and proceeded for qPCR as mentioned earlier.

RNA extraction

The right middle lobe of the lung tissues were immediately placed in RNAlater solution, then held on ice for 4–5 h, and then stored at -80°C until the RNA extraction procedure. RNA extraction was performed using RNeasy Mini Kits from Qiagen (Valencia, CA). RNA concentrations were measured with a NanoDrop One (ThermoFisher). One microgram of total RNA was reverse transcribed to cDNA using Bio-Rad iScript cDNA synthesis kit (Hercules, CA) according to the manufacturer's instructions.

Real-time PCR analyses

Expression of the target gene was quantified with gene-specific primers and SYBRGreen master mix using a QuantStudio 3 Real-Time PCR instrument from ThermoFisher Scientific. The housekeeping gene Actin, beta was used as the loading control for the human. The gene expression values were calculated based on the $\Delta\Delta$Ct method. Primer sequences are given in Supplemental Table 1 under oligonucleotides section.

CB_1R antibody optimization

To optimize the CB_1R staining by flow cytometry, we have used the CB_1R expressing HEK293 cell line developed from the laboratory of Ken Mackie, Indiana University. The cells were cultured in DMEM (with 4.5g/L glucose, L-glutamine, and pyruvate), 10% FBS, 1 mg/mL G418 & 1% PenStrep at 37°C in a 5% CO2 incubator. First, to investigate the CB_1R expression and functionality in this cell line we have performed CB_1R binding assay and GTPγS binding assay as previously described (7, 13), respectively (Supplemental Figure 6, A and B). The binding affinity of rimonabant (CB_1R antagonist) was determined in radioligand displacement assays using 1 nM $[^3H]$CP55940 as the agonist radioligand

in CB_1R expressing HEK293 cell membranes (Supplemental Figure 6B). GTPγS-functional assay was performed using [^{35}S]-GTPγS- and CB_1R expressing HEK293 cell membranes in the presence of the agonist CP55,940 (1 μM) to determine functional CB_1R in HEK293 cells. Then, we used Anti-CB_1R (extracellular)-FITC polyclonal antibody directed against the extracellular N-terminus of the CB_1R for CB_1R staining by flow cytometry. For gating the CB_1R-positive population, we have compared it with rabbit IgG isotype control-FITC at a similar concentration (Supplemental Figure 6C).

Immunophenotyping by flow cytometry

Immunophenotyping for macrophages by flow cytometry was performed as previously described with slight modifications (14, 15). Briefly, the right lower lobe of the lung tissue was immediately placed in MACS tissue storage solution (Miltenyi Biotech) and processed for digestion using Lung Dissociation Kit, mouse, and gentleMACS™ dissociator (Miltenyi Biotech) according to the manufacturer's instructions. Following dissociation, single-cell suspension was obtained from each sample by filtering through a 70 μm filter (MACS SmartStrainer, Miltenyi Biotech). Cells were first stained with LIVE/DEAD™ Fixable Near-IR Dead Cell Stain Kit (Invitrogen) in PBS for 30 min. After washing, the cells were incubated with Fc block (Anti-CD16/CD32) for 10 min followed by staining with a mixture of fluorochrome-conjugated antibodies in Brilliant Stain Buffer with two different panels of antibodies (Supplemental Table 2 and Supplemental Table 3). The tubes were acquired on CytoFLEX flow cytometer (Beckman Coulter) using CytExpert software. Data analyses were performed using FlowJo software (v10, BD Biosciences). Cell populations were identified using a sequential gating strategy (Supplemental Figure

5 and 7) and fluorescence minus one (FMO) control was used to fix the gate (15). For CB_1R gating, IgG isotype control-FITC was used to fix the gate. For MH-S cell line, the cells were removed from the T25 flask using scrapper and then stained with LIVE/DEAD™ Fixable Near-IR Dead Cell Stain Kit followed by CB_1R-FITC and CD206-PE as mentioned above.

Multiplex cytokine analysis by Luminex

Cytokines, chemokines, and other secretory factors from the BALF of each mouse were quantified using Luminex Mouse Discovery Assay (25-Plex) LXSAMSM-25 (R & D Systems), Immune Monitoring 48-Plex Mouse ProcartaPlex™ Panel (ThermoFisher Scientific) and ProcartaPlex™ Human Immune Response Panel, 80plex (ThermoFisher Scientific) according to the manufacturer's instructions. Magnetic Beads were added to a 96-well plate and washed. Fifty microliters of BALF samples for the mouse kit and 50 µL of culture supernatant from hPCLS culture for the human kit were added to the plate containing the mixed antibody-conjugated beads and incubated at room temperature for 1 h followed by overnight incubation at 4°C on an orbital shaker at 600 rpm. Following the overnight incubation, plates were washed and then the biotinylated detection antibody was added for 60 min at room temperature with shaking. The plate was washed, and streptavidin-PE was added. After incubation for 30 min at room temperature followed by washing, the reading buffer was added to the wells. Each sample was measured in duplicate. Plates were read using a Luminex 200 instrument (Luminex corporation) with a lower bound of 50 beads per sample per analyte. The results were analyzed using xPONENT 4.2 software (Luminex corporation) using a 4-PL fitting.

Gene expression analysis using nCounter Fibrosis Panel

Gene expression was quantified using the nCounter® Fibrosis Panel (nanoString). Three samples from each group were processed. RNA was diluted to 100 ng and a thermocycler pre-heated to 65°C. Reporter and capture probes were thawed to room temperature. A master mix containing 5 µL of hybridization buffer and 3 µL of the reporter probes was added to each well of a 12-well strip. Five microliters of the sample were added to each well, followed by 2 µL of the capture probes. The strips were capped and mixed by inverting and placed in a thermocycler at 65°C overnight for 20 h. Following hybridization, samples were transferred in the 12 well strips to the nCounter automatic prep station, where excess probes were removed through a two-step magnetic bead-based purification. The purified complexes were eluted and immobilized on a cartridge for counting using the nCounter Digital Analyzer (nanoString). Count data were exported as reporter code count (RCC) files. RCC files were imported into the nanoString nSolver software for normalization using positive and negative probes. For pathway analysis, the reference data file annotated with different functions for 770 genes in the fibrosis panel was obtained using the Advanced Analysis package in the nSolver Analysis Software.

RNA Sequencing

RNA Quality control, library preparations, sequencing reactions, and initial bioinformatic analysis were conducted at GENEWIZ, LLC (South Plainfield, NJ, USA). RNA samples were quantified using Qubit 2.0 Fluorometer (Life Technologies, Carlsbad, CA, USA) and RNA integrity was checked using Agilent TapeStation 4200 (Agilent Technologies, Palo

Alto, CA, USA). RNA-sequencing libraries were prepared using the NEBNext Ultra II RNA Library Prep Kit for Illumina using manufacturer's instructions (NEB, Ipswich, MA, USA). Briefly, mRNAs were first enriched with Oligo(dT) beads. Enriched mRNAs were fragmented for 15 min at 94°C. First strand and second strand cDNAs were subsequently synthesized. cDNA fragments were end repaired and adenylated at 3'ends, and universal adapters were ligated to cDNA fragments, followed by index addition and library enrichment by limited-cycle PCR. The sequencing libraries were validated on the Agilent TapeStation (Agilent Technologies, Palo Alto, CA, USA), and quantified by using Qubit 2.0 Fluorometer (Invitrogen, Carlsbad, CA) as well as by quantitative PCR (KAPA Biosystems, Wilmington, MA, USA). The sequencing libraries were clustered on flowcells. After clustering, the flowcells were loaded on to the Illumina HiSeq instrument (4000 or equivalent) according to manufacturer's instructions. The samples were sequenced using a 2 × 150 bp Paired End configuration. Image analysis and base calling were conducted by the HiSeq Control Software. Raw sequence data (.bcl files) generated from Illumina HiSeq was converted into fastq files and de-multiplexed using Illumina's bcl2fastq 2.17 software. One mismatch was allowed for index sequence identification.

Spatial and Single Cell RNA-sequencing data reanalysis

We retrieved healthy and IPF lung single cell and spatial RNA-sequencing data from previously published data (16). The data was downloaded from the original data repository (https://zenodo.org/records/10012934). We used SCANPY 1.9.8 on Python 3.11 for the data analysis. The gene expression was shown using the dot plot functions in SCANPY based on the "Niche_NMF" columns in the observation variable.

Human Precision-Cut Lung Slice Source Tissue

Precision-cut lung slices (hPCLS) were obtained from explanted lung lobes of de-identified male and female donors aged 40–70 years. Lung lobes were flushed with sterile saline supplemented with 1% PenStrep and 2.5 µg/mL of Amphotericin B via the main bronchus and blood vessels to remove excess blood. Posteriorly, lobes were inflated with a solution of 2.5% low-melting point agarose in DMEM/Nutrient Mixture F12 supplemented with 1% of PenStrep, 2.5 µg/mL of Amphotericin B, and 1% of heat-inactivated fetal bovine serum (supplemented DMEM/F12) maintained at 37-40°C at a constant pressure and speed (~1 mL/sec). Once the lobe was filled with the molten agarose solution, the tissue was maintained on ice for 30–45 mins until the agarose was completely jellified. Thereafter, the tissue was cut sagittally into 2.5 cm thick slices and examined for possible abnormalities and filling consistency. Tissue cylindrical cores of 10 mm in diameter were excised with a coring tool (Alabama Research and Development, Munford, AL) from the lung parenchyma. Posteriorly, 300 µm thick hPCLS are sliced from the tissue cores using a Compresstome vibroslicer (Precisionary Instruments LLC.) and collected in supplemented DMEM/F12 maintained on ice. Afterward, hPCLS are distributed on 24-well tissue culture plates and maintained under standard tissue culture conditions (37°C, 95% O2, high humidity).

Human precision-cut lung slices (hPCLS) culture and MRI-1867 treatment

hPCLS (1 cm in diameter and 300 µM in thickness) were generated as previously described (17, 18) and cultured for 120 h. The fibrosis cocktail (FC) was prepared by the

addition as described earlier and its vehicle (diluent) in parallel for the control cocktail (CC) in a medium supplemented with 0.1% FBS and 1% PenStrep. FC consisted of 5 ng/mL recombinant transforming growth factor-β (TGF-β), 5 μM platelet-derived growth factor-AB (PDGF-AB), 10 ng/mL tumor necrosis factor-α (TNF-α), and 5 μM lysophosphatidic acid (LPA) and was replenished at 48 h. After 48 h, MRI-1867 (10 μM) was added to the CC and FC media for another 72 h to evaluate the antifibrotic efficacy. After 120 h, the supernatants from all the hPCLS are stored for further analysis (Luminex and Mass-Spec). All hPCLS were distributed for downstream analysis such as histology (following fixation), RNA isolation (qPCR), and hydroxyproline measurement.

References

1. Busch CJ, Favret J, Geirsdottir L, Molawi K, and Sieweke MH. Isolation and Long-term Cultivation of Mouse Alveolar Macrophages. *Bio Protoc.* 2019;9(14).
2. Zimmer A, Zimmer AM, Hohmann AG, Herkenham M, and Bonner TI. Increased mortality, hypoactivity, and hypoalgesia in cannabinoid CB1 receptor knockout mice. *Proc Natl Acad Sci U S A.* 1999;96(10):5780-5.
3. Gonzalez-Mariscal I, Montoro RA, Doyle ME, Liu QR, Rouse M, O'Connell JF, et al. Absence of cannabinoid 1 receptor in beta cells protects against high-fat/high-sugar diet-induced beta cell dysfunction and inflammation in murine islets. *Diabetologia.* 2018;61(6):1470-83.
4. Cinar R, Gochuico BR, Iyer MR, Jourdan T, Yokoyama T, Park JK, et al. Cannabinoid CB1 receptor overactivity contributes to the pathogenesis of idiopathic pulmonary fibrosis. *JCI Insight.* 2017;2(8).
5. Cinar R, Iyer MR, Liu Z, Cao Z, Jourdan T, Erdelyi K, et al. Hybrid inhibitor of peripheral cannabinoid-1 receptors and inducible nitric oxide synthase mitigates liver fibrosis. *JCI Insight.* 2016;1(11).
6. Iyer MR, Cinar R, Coffey NJ, and Kunos G. Synthesis of (13) C(6) -labeled, dual-target inhibitor of cannabinoid-1 receptor (CB(1) R) and inducible nitric oxide synthase (iNOS). *J Labelled Comp Radiopharm.* 2018.
7. Iyer MR, Cinar R, Liu J, Godlewski G, Szanda G, Puhl H, et al. Structural Basis of Species-Dependent Differential Affinity of 6-Alkoxy-5-Aryl-3-Pyridinecarboxamide Cannabinoid-1 Receptor Antagonists. *Mol Pharmacol.* 2015;88(2):238-44.
8. Park JK, Coffey NJ, Bodine SP, Zawatsky CN, Jay L, Gahl WA, et al. Bleomycin Induces Drug Efflux in Lungs. A Pitfall for Pharmacological Studies of Pulmonary Fibrosis. *Am J Respir Cell Mol Biol.* 2020;62(2):178-90.
9. Devos FC, Maaske A, Robichaud A, Pollaris L, Seys S, Lopez CA, et al. Forced expiration measurements in mouse models of obstructive and restrictive lung diseases. *Respir Res.* 2017;18(1):123.
10. McGovern TK, Robichaud A, Fereydoonzad L, Schuessler TF, and Martin JG. Evaluation of respiratory system mechanics in mice using the forced oscillation technique. *J Vis Exp.* 2013(75):e50172.
11. Zawatsky CN, Park JK, Abdalla J, Kunos G, Iyer MR, and Cinar R. Peripheral Hybrid CB1R and iNOS Antagonist MRI-1867 Displays Anti-Fibrotic Efficacy in Bleomycin-Induced Skin Fibrosis. *Front Endocrinol (Lausanne).* 2021;12:744857.
12. Strunz M, Simon LM, Ansari M, Kathiriya JJ, Angelidis I, Mayr CH, et al. Alveolar regeneration through a Krt8+ transitional stem cell state that persists in human lung fibrosis. *Nat Commun.* 2020;11(1):3559.
13. Cinar R, and Szucs M. CB1 receptor-independent actions of SR141716 on G-protein signaling: coapplication with the mu-opioid agonist Tyr-D-Ala-Gly-(NMe)Phe-Gly-ol unmasks novel, pertussis toxin-insensitive opioid signaling in mu-opioid receptor-Chinese hamster ovary cells. *J Pharmacol Exp Ther.* 2009;330(2):567-74.
14. Pommerolle L, Beltramo G, Biziorek L, Truchi M, Dias AMM, Dondaine L, et al. CD206(+) macrophages are relevant non-invasive imaging biomarkers and therapeutic targets in experimental lung fibrosis. *Thorax.* 2024.

15. Misharin AV, Morales-Nebreda L, Mutlu GM, Budinger GR, and Perlman H. Flow cytometric analysis of macrophages and dendritic cell subsets in the mouse lung. *Am J Respir Cell Mol Biol.* 2013;49(4):503-10.
16. Mayr CH, Santacruz D, Jarosch S, Bleck M, Dalton J, McNabola A, et al. Spatial transcriptomic characterization of pathologic niches in IPF. *Sci Adv.* 2024;10(32):eadl5473.
17. Alsafadi HN, Staab-Weijnitz CA, Lehmann M, Lindner M, Peschel B, Konigshoff M, et al. An ex vivo model to induce early fibrosis-like changes in human precision-cut lung slices. *Am J Physiol Lung Cell Mol Physiol.* 2017;312(6):L896-L902.
18. Uhl FE, Vierkotten S, Wagner DE, Burgstaller G, Costa R, Koch I, et al. Preclinical validation and imaging of Wnt-induced repair in human 3D lung tissue cultures. *The European respiratory journal.* 2015;46(4):1150-66.

Supplemental Table 1: Key resources table

REAGENT or RESOURCE	SOURCE	IDENTIFIER
**Antibodies**		
PerCP anti-mouse CD45 Antibody	Biolegend	Cat# 103130, Clone: 30-F11
Alexa Fluor® 488 Rat Anti-CD11b	BD Biosciences	Cat# 557672, Clone: M1/70
BV605 Hamster Anti-Mouse CD11c	BD Biosciences	Cat# 563057, Clone: HL3
PE/Fire™ 810 anti-mouse Ly-6G Antibody	Biolegend	Cat# 127673, Clone: 1A8
APC anti-mouse CD64 (FcγRI) Antibody	Biolegend	Cat# 139306, Clone: X54-5/7.1
BV650 Rat Anti-Mouse Siglec-F	BD Biosciences	Cat# 740557, Clone: E50-2440
BV421 Hamster Anti-Mouse CD80	BD Biosciences	Cat# 562611, Clone: 16-10A1
PE anti-mouse CD206 (MMR) Antibody	Biolegend	Cat# 141706, Clone: C068C2
BV786 Rat Anti-Mouse I-A/I-E	BD Biosciences	Cat# 742894, Clone: M5/114
R718 Rat Anti-Mouse CD11b	BD Biosciences	Cat# 567469, Clone: M1/70
BV510 Hamster Anti-Mouse CD11c	BD Biosciences	Cat# 562949, Clone: HL3
BV421 Rat Anti-Mouse CD24	BD Biosciences	Cat# 562563, Clone: M1/69
Brilliant Violet 650™ anti-mouse CD80 Antibody	Biolegend	Cat# 104731, Clone: 16-10A1
Anti-Cannabinoid Receptor 1 (extracellular)-FITC Antibody	alomone labs	Cat# ACR-001-F
Rabbit IgG Isotype Control-FITC	alomone labs	Cat# RIC-001-F
**Chemicals, Peptides, and Recombinant Proteins**		
Bleomycin for Injection	USPPfizer Hospital US	Cat# 61703-0323-22
0.9% Sodium Chloride Injection	USPPfizer Hospital US	Cat# 00409-4888-50
Zetamine (Ketamine)	VetOne	Cat# 501072
AnaSed Injection (Xylazine)	Akorn	Cat# NDC 59399-110-20
Isoflurane	VetOne	Cat# 501017
Dimethyl sulfoxide	Millipore Sigma	Cat# D2650
Tween 80	Millipore Sigma	Cat# P4780
Formalin solution, neutral buffered, 10%	Millipore Sigma	Cat# HT501128-4L
Paraffin wax	Millipore Sigma	Cat# 76242-1KG
Xylenes	Millipore Sigma	Cat# XX0060
Alcohol	Millipore Sigma	Cat# AX0442
Water, Nuclease-free, Molecular Biology Grade, Ultrapure	ThermoFisher Scientific	Cat# J71786.XCR
Perchloric acid	Millipore Sigma	Cat# 244252
Hydrochloric acid	Millipore Sigma	Cat# 320331
L-Proline-$^{13}C_5$,^{15}N	Millipore Sigma	Cat# 608114
Formic acid	Millipore Sigma	Cat# 27001
Acetonitrile	Millipore Sigma	Cat# 34998
Isopropanol	Millipore Sigma	Cat# 278475
trans-4-Hydroxy-L-proline	Millipore Sigma	Cat# H54409
Chloroform	Millipore Sigma	Cat# 366927
Tetrahydrofuran	Millipore Sigma	Cat# 34865

REAGENT or RESOURCE	SOURCE	IDENTIFIER
Ammonium formate	Millipore Sigma	Cat# 70221
RNA*later*™ Stabilization Solution	ThermoFisher Scientific	Cat# AM7020
SYBR™ Green PCR Master Mix	ThermoFisher Scientific	Cat# 4309155
MACS Tissue Storage Solution	Miltenyi Biotech	Cat# 130-100-008
PBS, pH 7.4	ThermoFisher Scientific	Cat# 10010023
BD Pharmingen™ Stain Buffer (BSA)	BD Biosciences	Cat# 554657
BD Horizon™ Brilliant Stain Buffer	BD Biosciences	Cat# 563794
BD Cytofix™ Fixation Buffer	BD Biosciences	Cat# 554655
F-12K Medium (Kaighn's Modification of Ham's F-12 Medium)	ATCC	Cat# 30-2004
Trypsin-EDTA Solution, 1X	ATCC	Cat# 30-2101
RPMI-1640	ATCC	Cat# 30-2001
2-Mercaptoethanol	ThermoFisher Scientific	Cat# 21985023
Fetal Bovine Serum (FBS)	ATCC	Cat# 30-2020
RPMI 1640 Medium, no glutamine	ThermoFisher Scientific	Cat# 31870025
GlutaMAX Supplement	ThermoFisher Scientific	Cat# 35050038
Sodium Pyruvate	ThermoFisher Scientific	Cat# 11360070
Mouse GM-CSF Recombinant Protein	ThermoFisher Scientific	Cat# 315-03-100UG
Dulbecco's Phosphate Buffered Saline (D-PBS), 1X	ATCC	Cat# 30-2200
Ham's F-12 Nutrient Mix	ThermoFisher Scientific	Cat# 11765054
DMEM/F12, powder with HEPES	ThermoFisher Scientific	Cat# 12-400-024
Low Melt Agarose	Goldbio	Cat# A-204-100
Fetal Bovine Serum, qualified, heat-inactivated, United States	ThermoFisher Scientific	Cat# 16140071
L-Glutamine (200 mM)	ThermoFisher Scientific	Cat# A2916801
Bovine Pituitary Extract (BPE)	ThermoFisher Scientific	Cat# 13028014
Human IGF-I Recombinant Protein, PeproTech®	ThermoFisher Scientific	Cat# 100-11-1MG
Transferrin, Bovine, lyophilized	ThermoFisher Scientific	Cat# 11108016
Rat EGF Recombinant Protein, PeproTech®	ThermoFisher Scientific	Cat# 400-25-1MG
Dimethylsulfoxide (DMSO)	ATCC	Cat# 4-X
DMEM, high glucose, pyruvate	ThermoFisher Scientific	Cat# 11995073
Geneticin™ Selective Antibiotic (G418 Sulfate) (50 mg/mL)	ThermoFisher Scientific	Cat# 10131035
Penicillin-Streptomycin (5,000 U/mL)	ThermoFisher Scientific	Cat# 15070063
Arachidonyl-2′-chloroethylamide hydrate (ACEA)	Millipore Sigma	Cat# A9719
Charcoal powder	Millipore Sigma	Cat# 1.02204
Gum arabic from acacia tree	Millipore Sigma	Cat# 51198
Corning® Dispase	Millipore Sigma	Cat# CLS354235
Collagenase Type I, Cls I	Millipore Sigma	Cat# C1-22-1G
Elastase from porcine pancreas	Millipore Sigma	Cat# E1250-100MG
DNase I	Millipore Sigma	Cat# 10104159001
MACS BSA Stock Solution	Miltenyi Biotech	Cat# 130-091-376
autoMACS® Rinsing Solution	Miltenyi Biotech	Cat# 130-091-222
Recombinant Human TGF-beta 1 Protein	R&D Systems	Cat# 240-B-002/CF

REAGENT or RESOURCE	SOURCE	IDENTIFIER
Recombinant Human TNF-alpha Protein, CF	R&D Systems	Cat# 10291-TA-100
Human PDGF-AB Recombinant Protein, CF	R&D Systems	Cat# 222-AB-010
1-Oleoyl-2-hydroxy-*sn*-glycero-3-PA (sodium salt)	Cayman Chemical	Cat# 62215
GTPγS, [^{35}S]- 1250Ci/mmol, 12.5mCi/ml, 250 µCi	revvity	Cat# NEG030H250UC
CP 55940, [Side Chain-2,3,4-^{3}H(N)]-, 250µCi (9.25MBq)	revvity	Cat# NET1051250UC
Rimonabant	Cayman Chemical	Cat# 9000484
(−)-CP 55,940	Cayman Chemical	Cat# 90084
1400W (hydrochloride)	Cayman Chemical	Cat# 81520
2-Arachidonoyl Glycerol-d_5	Cayman Chemical	Cat# 362162
**Critical Commercial Assays**		
Epredia™ Richard-Allan Scientific™ Masson Trichrome Kit	Fisher Scientific	Cat# 22-110-648
RNeasy Plus Universal Mini Kit	QIAGEN	Cat# 73404
iScript™ cDNA Synthesis Kit, 500 x 20 µl rxns	BIO-RAD	Cat# 1708891BUN
Lung Dissociation Kit, mouse	Miltenyi Biotech	Cat# 130-095-927
LIVE/DEAD™ Fixable Near-IR Dead Cell Stain Kit, for 633 or 635 nm excitation	Invitrogen	Cat# L10119
Luminex Mouse Discovery Assay (25-Plex)	R & D Systems	Cat# LXSAMSM-25
Immune Monitoring 48-Plex Mouse ProcartaPlex™ Panel	ThermoFisher Scientific	Cat# EPX480-20834-901
nCounter Mouse Fibrosis V2 Panel	nanoString	Cat# XT-CSO-MFIB2-12
RNeasy Plus Mini Kit (250)	QIAGEN	Cat# 74136
ProcartaPlex™ Human Immune Response Panel, 80plex	ThermoFisher Scientific	Cat# EPX800-10080-901
Dead Cell Removal Kit	Miltenyi Biotech	Cat# 130-090-101
Anti-F4/80 MicroBeads UltraPure, mouse	Miltenyi Biotech	Cat# 130-110-443
Deposited Data		
Mouse Transcriptomics Data	GEO Database	GSE273132
**Experimental Models: Cell Lines**		
RLE-6TN	ATCC	Cat# CRL-2300
NR8383 [AgC11x3A, NR8383.1]	ATCC	Cat# CRL-2192
CB1 Cannabinoid Receptor Expressing HEK293 Cell Lines	kerafast	Cat# EIU005
MH-S	ATCC	Cat# CRL-2019
**Experimental Models: Organisms/Strains**		
C57BL/6J	The Jackson Laboratory	RRID# IMSR_JAX:000664
CB1R KO Mice	Dr. Andreas Zimmer, Laboratories of Genetics and Cellular and Molecular Regulation, National Institute of Mental Health	Zimmer et al., 1999. Proc Natl Acad Sci USA. 1999;96(10):5780-5. doi: 10.1073/pnas.96.10.5780.
LysMcre (B6.129P2-*Lyz2*$^{tm1(cre)Ifo}$/J)	The Jackson Laboratory	RRID# IMSR_JAX:004781
Sftpc-CreER^{T2}(B6.129S-*Sftpc*$^{tm1(cre/ERT2)Blh}$/J)	The Jackson Laboratory	RRID# IMSR_JAX:028054

REAGENT or RESOURCE	SOURCE	IDENTIFIER
CB1R flox/flox	Dr. Josephine Egan, Laboratory of Clinical Investigation, National Institute on Aging	González-Mariscal et al., 2018 Diabetologica. 2018;61(6):1470-1483. doi: 10.1007/s00125-018-4576-4
**Oligonucleotides**		
Mm_Tbp_1_SG QuantiTect Primer Assay	QIAGEN	GeneGlobe Id: QT00198443
Mm_Cnr1_2_SG QuantiTect Primer Assay	QIAGEN	GeneGlobe Id: QT01748831
Mm_Nos2_1_SG QuantiTect Primer Assay	QIAGEN	GeneGlobe Id: QT00100275
Mm_Arg1_1_SG QuantiTect Primer Assay	QIAGEN	GeneGlobe Id: QT00134288
Mm_Spp1_1_SG QuantiTect Primer Assay	QIAGEN	GeneGlobe Id: QT00157724
Mm_Pdgfa_1_SG QuantiTect Primer Assay	QIAGEN	GeneGlobe Id: QT00197610
Mm_Mafb_1_SG QuantiTect Primer Assay	QIAGEN	GeneGlobe Id: QT00314727
Mm_Mertk_1_SG QuantiTect Primer Assay	QIAGEN	GeneGlobe Id: QT00148561
Mm_Gpnmb_1_SG QuantiTect Primer Assay	QIAGEN	GeneGlobe Id: QT00164059
Mm_Lgmn_1_SG QuantiTect Primer Assay	QIAGEN	GeneGlobe Id: QT00108052
Mm_Chil3_2_SG QuantiTect Primer Assay	QIAGEN	GeneGlobe Id: QT02241722
Mm_Cd63_1_SG QuantiTect Primer Assay	QIAGEN	GeneGlobe Id: QT00325535
Mm_Fabp5_1_SG QuantiTect Primer Assay	QIAGEN	GeneGlobe Id: QT00240226
Mm_Trem2_1_SG QuantiTect Primer Assay	QIAGEN	GeneGlobe Id: QT00157969
Mm_Tgfb1_1_SG QuantiTect Primer Assay	QIAGEN	GeneGlobe Id: QT00145250
Mm_Retnla_1_SG QuantiTect Primer Assay	QIAGEN	GeneGlobe Id: QT00254359
Mm_Cd163_1_SG QuantiTect Primer Assay	QIAGEN	GeneGlobe Id: QT00123074
Mm_Fabp4_1_SG QuantiTect Primer Assay	QIAGEN	GeneGlobe Id: QT00091532
Mm_Cd9_2_SG QuantiTect Primer Assay	QIAGEN	GeneGlobe Id: QT01752513
Mm_S100a4_1_SG QuantiTect Primer Assay	QIAGEN	GeneGlobe Id: QT00107632
Mm_Il6_1_SG QuantiTect Primer Assay	QIAGEN	GeneGlobe Id: QT00098875
Hs_ACTB_2_SG QuantiTect Primer Assay	QIAGEN	GeneGlobe Id: QT01680476
Hs_ACTA2_1_SG QuantiTect Primer Assay	QIAGEN	GeneGlobe Id: QT00088102
Hs_FN1_1_SG QuantiTect Primer Assay	QIAGEN	GeneGlobe Id: QT00038024
Hs_COL1A1_1_SG QuantiTect Primer Assay	QIAGEN	GeneGlobe Id: QT00037793
Hs_IRF5_1_SG QuantiTect Primer Assay	QIAGEN	GeneGlobe Id: QT00092736
**Software and Algorithms**		
GraphPad Prism, version 9.0.0	GraphPad	https://www.graphpad.com/scientific-software/prism/
CytExpert	Beckman Coulter	https://www.beckman.pt/flow-cytometry/research-flow-cytometers/cytoflex/software
FlowJo v.10.8.1	BD Biosciences	https://www.flowjo.com/solutions/flowjo/downloads/previous-versions
xPONENT 4.2	Luminex	https://www.luminexcorp.com/xponent/#overview
Zen Blue 3.1	Zeiss	https://www.zeiss.com/microscopy/en/products/software/zeiss-zen.html

REAGENT or RESOURCE	SOURCE	IDENTIFIER
nSolver 4.0	nanoString	https://nanostring.com/products/analysis-solutions/ncounter-analysis-solutions/
Python 3.7	Python Package	RRID:SCR_019053
R	R Project for Statistical Computing	RRID:SCR_001905
Scikit-Learn	Python Package	RRID:SCR_019053
Rpy2	Python Package	https://rpy2.github.io/
SciPy	Python Package	RRID:SCR_008058
Statsmodel	Python Package	RRID:SCR_016074
iGraph	Python Package	RRID:SCR_019225
Statsmodel	Python Package	RRID:SCR_016074
DESeq2	R Package	RRID:SCR_015687

Supplemental Table 2: List of antibodies used in flow cytometry

Antibody	Fluorophore	Clone	Dilution
Anti-mouse CD45	PerCP	30-F11	1:50
Anti-mouse CD11b	R718	M1/70	1:50
Anti-mouse CD11c	BV510	HL3	1:25
Anti-mouse Ly-6G	PE/Fire™ 810	1A8	1:50
Anti-mouse CD64	APC	X54-5/7.1	1:25
Anti-mouse CD24	BV421	M1/69	1:50
Anti-Mouse I-A/I-E (MHC-II)	BV786	M5/114	1:50
Anti-mouse CD80	BV650	16-10A1	1:25
Anti-mouse CD206	PE	C068C2	1:25
Anti-CB_1R (extracellular)	FITC	polyclonal	1:50

Supplemental Table 3: List of antibodies used in flow cytometry

Antibody	Fluorophore	Clone	Dilution
Anti-mouse CD45	PerCP	30-F11	1:50
Anti-mouse CD11b	AF488	M1/70	1:50
Anti-mouse CD11c	BV605	HL3	1:25
Anti-mouse Ly-6G	PE/Fire™ 810	1A8	1:50
Anti-mouse CD64	APC	X54-5/7.1	1:25
Anti-mouse Siglec-F	BV650	E50-2440	1:20
Anti-Mouse I-A/I-E (MHC-II)	BV786	M5/114	1:50
Anti-mouse CD80	BV421	16-10A1	1:25
Anti-mouse CD206	PE	C068C2	1:25

Supplementary Figure 1

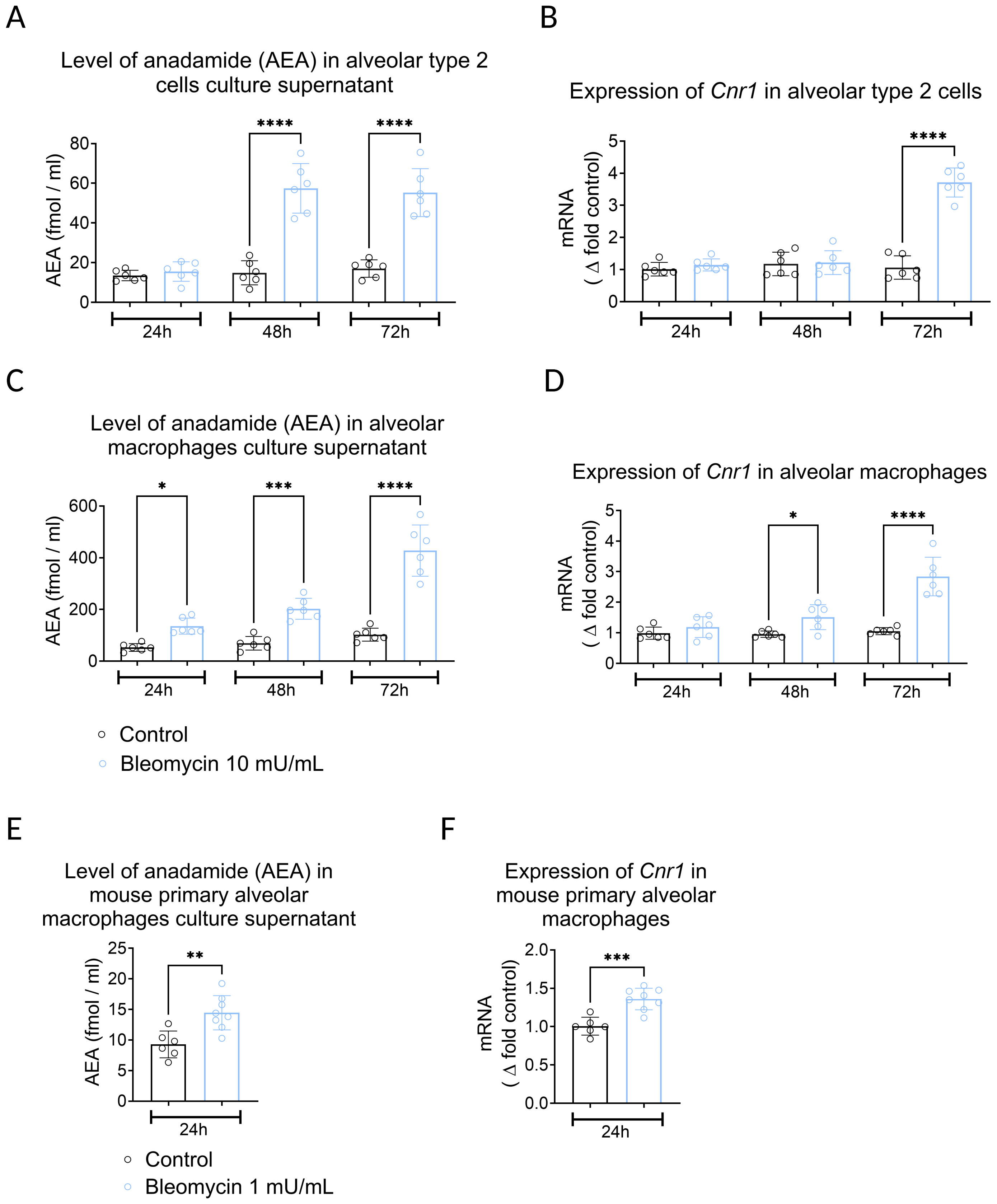

Supplementary Figure 1. Bleomycin induced activation of the endocannabinoid system in AT2 cells, Ams, and primary mouse AMs

(A) Level of anandamide (AEA) in AT2 cell line over 72 h exposure of bleomycin 10 mU/mL (one-way ANOVA, ****$p < 0.0001$, n = 6 per group)

(B) Expression of Cnr1 in AT2 cell line over 72 h exposure of bleomycin 10 mU/mL (one-way ANOVA, ****$p < 0.0001$, n = 6 per group)

(C) Level of anandamide (AEA) in AMs cell line over 72 h exposure of bleomycin 10 mU/mL one-way ANOVA, ****$p < 0.0001$, ***$p < 0.001$, *$p < 0.05$, n = 6 per group)

(D) Expression of Cnr1 in AMs cell line over 72 h exposure of bleomycin 10 mU/mL (one-way ANOVA, ****$p < 0.0001$, *$p < 0.05$, n = 6 per group)

(E) Level of anandamide (AEA) in primary mouse AMs over 24 h exposure of bleomycin 1 mU/mL (one-way ANOVA, **$p < 0.01$, n = 6-8 per group)

(F) Expression of Cnr1 in primary mouse AMs over 24 h exposure of bleomycin 1 mU/mL (one-way ANOVA, ***$p < 0.001$, n = 6-8 per group)

Supplementary Figure 2

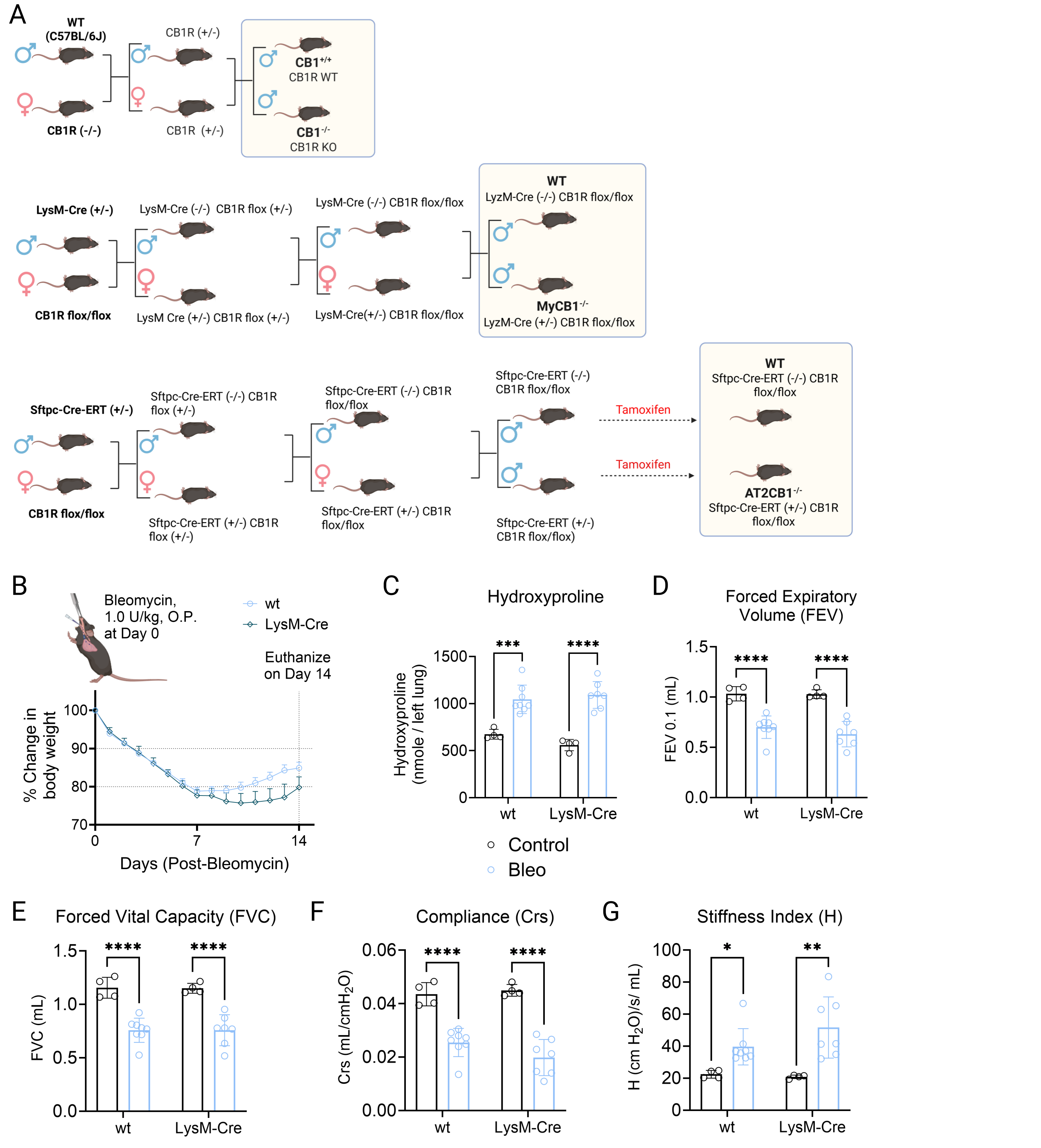

Supplementary Figure 2. Generation of conditional CB1R KO mice and fibrosis in LysM-Cre+ control mice
(A) Breeding strategy and generation of CB1R KO or CB1-/- (global CB1R KO), My-CB1R KO or MyCB1-/- (myeloid cell-specific CB1R KO), and AT2-CB1R KO or AT2CB1-/- (alveolar type 2 cell-specific CB1R KO).
(B) No change in the body weight loss was found between wt and LysM-Cre mice in 14 days bleomycin-induced pulmonary fibrosis model (n = 7-8 per group)
(C-G) No significant difference in the induction of fibrosis and pulmonary functions was found between wt and LysM-Cre mice in 14 days bleomycin-induced pulmonary fibrosis model (two-way ANOVA, ****$p < 0.0001$, ***$p < 0.001$, **$p < 0.01$, *$p < 0.05$, n = 4-8 per group)

Supplementary Figure 3

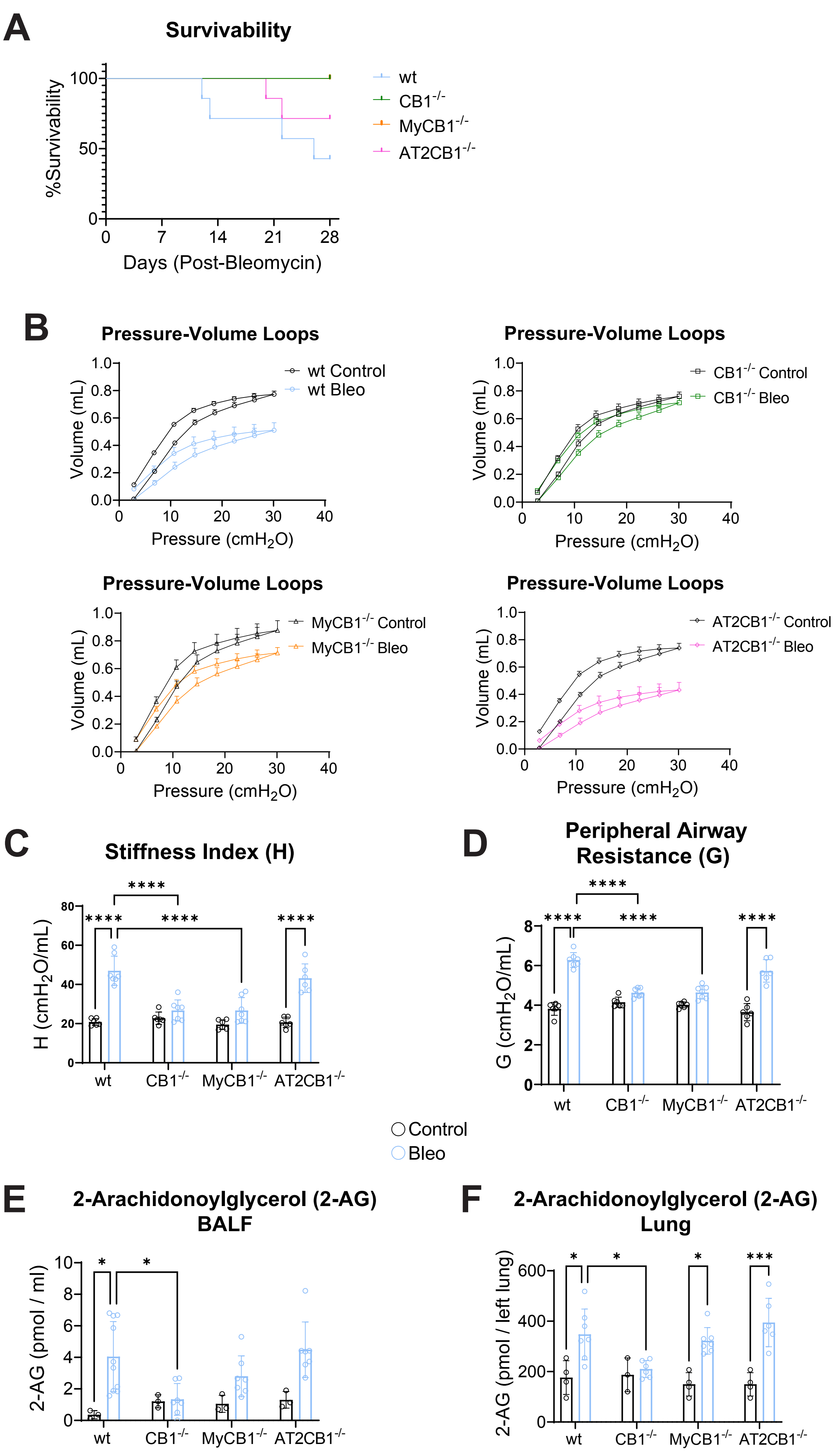

Supplementary Figure 3. Deletion of CB1R in myeloid cells, but not in AT2 cells, prevented mortality and improved pulmonary functions

(A) Survivability (Kaplan–Meier plot) among different groups showing no mortality in CB1R KO, and My-CB1R KO mice in 28 days bleomycin-induced PF model (n = 7-8 per group)

(B) Changes in the pressure-volume loops (PVP) among different groups compared to their respective control (n = 4-10 per group)

(C-D) Retention of pulmonary functions, stiffness index, and peripheral airway resistance at the normal levels was found in CB1R KO and myeloid-CB1R KO mice but not in AT2-CB1R KO mice after being challenged with bleomycin (two-way ANOVA, ****p < 0.0001, n = 6-7 per group)

(E-F) Levels of 2-arachidonyl glycerol (2AG) in BALF and lungs were inhibited in CB1R KO mice but not in myeloid-CB1R KO, and AT2-CB1R KO mice (two-way ANOVA, ***p < 0.001, *p < 0.05, n = 3-8 per group)

Supplementary Figure 4

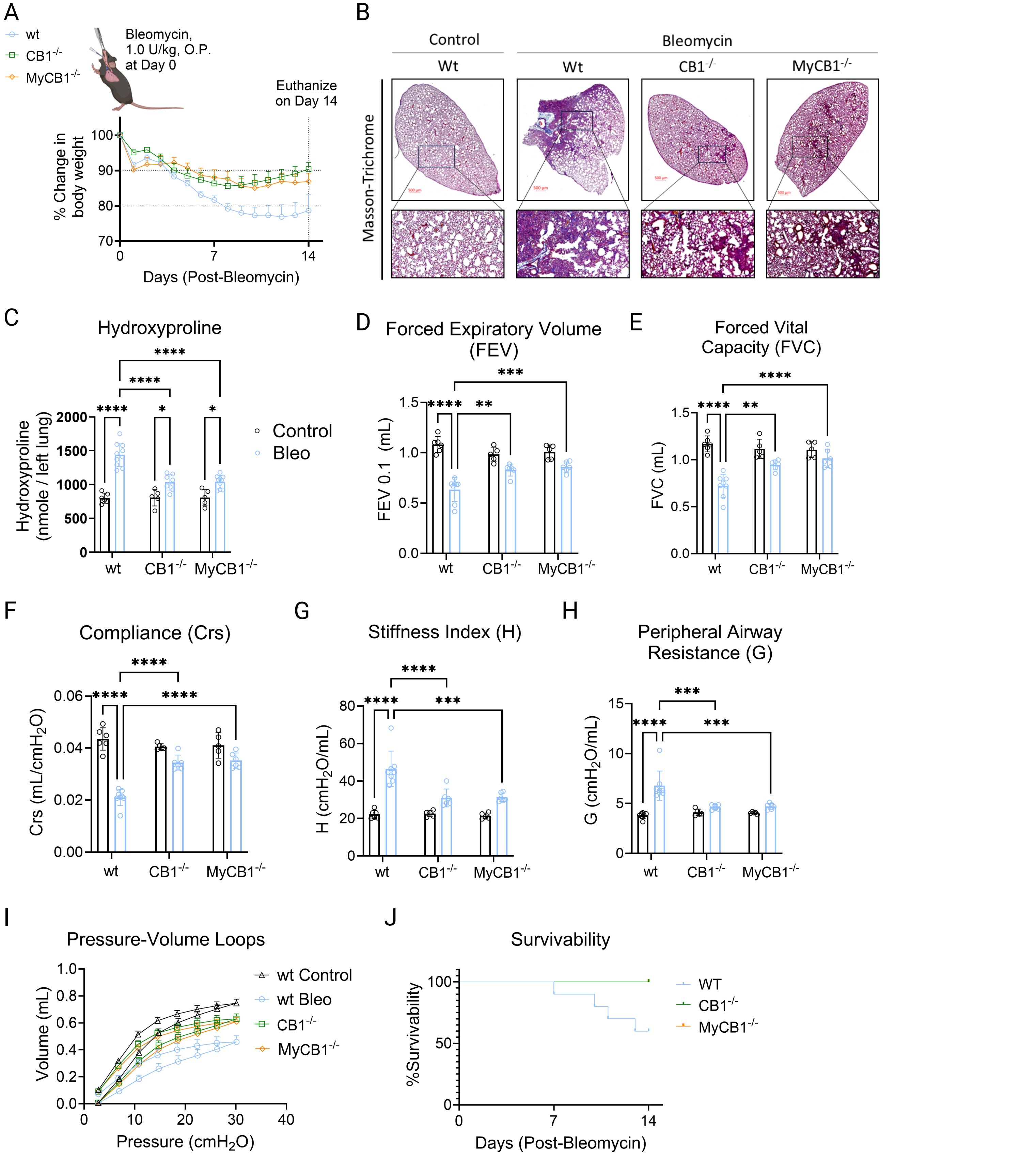

Supplementary Figure 4. Deletion of CB1R in myeloid cells is comparable to global deletion of CB1R in preventing pulmonary fibrosis

(A) Inhibition of loss of body weight in both CB1R KO and myeloid-CB1R KO mice in 14 days bleomycin-induced pulmonary fibrosis model (n = 8-10 per group)

(B) Representative images of lung sections showed reduced collagen deposition and improved histoarchitecture in CB1R KO and myeloid-CB1R KO mice upon Masson's trichrome staining

(C) Inhibition of the development of fibrosis was evident from significantly reduced hydroxyproline content in both CB1R KO and myeloid-CB1R KO mice at 14 days post-bleomycin (two-way ANOVA, ****p < 0.0001, *p < 0.05, n = 5-10 per group)

(D-H) Retention of pulmonary functions (FEV, FVC, compliance, stiffness index, and peripheral airway resistance) at the normal levels were found in both CB1R KO and myeloid-CB1R KO mice after being challenged with bleomycin (two-way ANOVA, ****p < 0.0001, ***p < 0.001, **p < 0.01, *p < 0.05, n = 5–8 per group).

(I) Changes in the pressure-volume loops (PVP) among different groups (n = 6–7 per group).

(J) Survivability (Kaplan–Meier plot) among different groups showing no mortality in CB1R KO, and My-CB1R KO mice in 14 days bleomycin-induced pulmonary fibrosis model (n = 8–10 per group).

Supplementary Figure 5

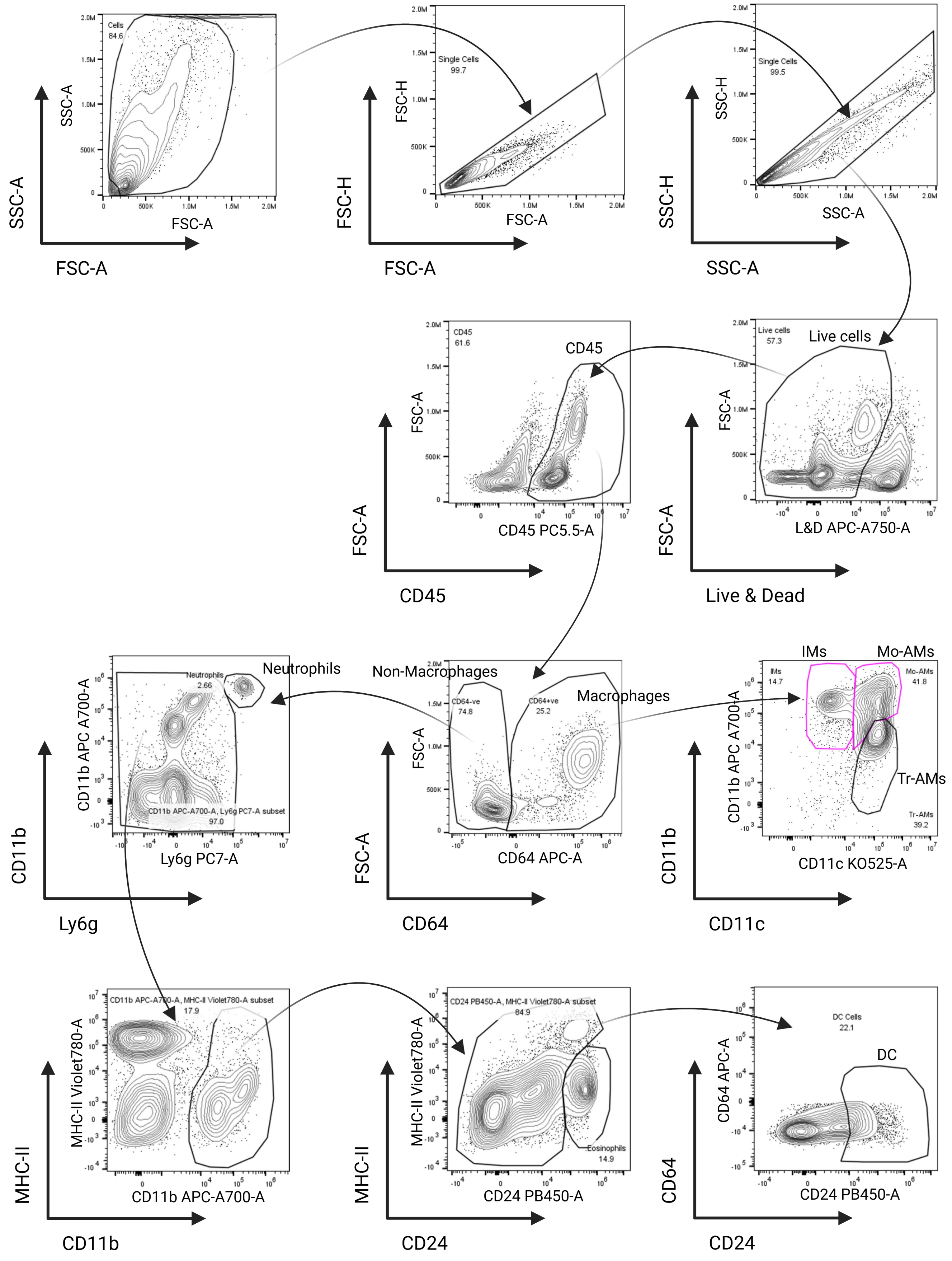

Supplementary Figure 5. Gating strategy to distinguish different subsets of myeloid cells in the mouse lung
Cells were isolated from enzymatically digested mouse lungs, and after the exclusion of doublets and debris, immune cells were identified by CD45 staining. First CD45+ve cells were subdivided into CD64+ve (macrophage) and CD64-ve (non-macrophage) populations. CD11b vs Ly6g was plotted from the CD64-ve population to identify neutrophils (CD11b+ve, Ly6g+ve/hi). After excluding neutrophils, MHC-II vs CD11b was plotted from the rest of the population, and the CD11b+ve population was selected for subsequent gating. On this population, MHC-II vs CD24 was plotted to identify eosinophils (CD24+ve, MHC-II-ve/lo). After excluding eosinophils, CD64 vs CD24 was plotted from the rest of the population, and dendritic cells were identified by CD24+ve population. Additionally, CD11b vs CD11c was plotted from CD64+ve (macrophage) population to distinguish between Tr-AMs (CD11c+ve, CD11b-ve), Mo-AMS (CD11C+ve, CD11b+ve), and IMs (CD11c-ve, CD11b+ve).

Supplementary Figure 6

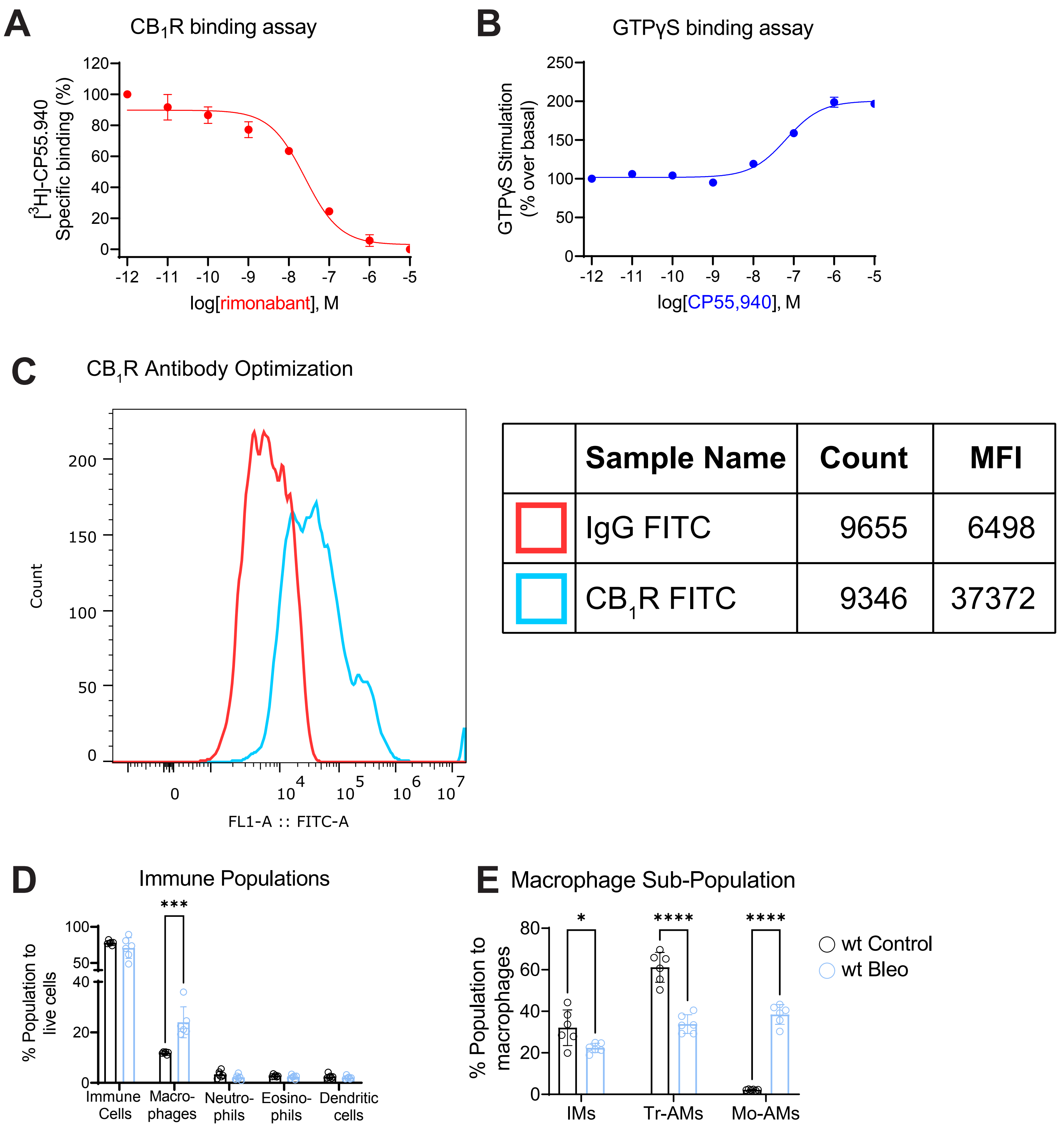

Supplementary Figure 6. CB1R antibody optimization and phenotypic alterations in the myeloid and macrophage populations induced by fibrosis

(A) CB1R binding affinity of the CB1R antagonist rimonabant in a [3H]-CP55,940 competition displacement binding assay using CB1R expressing HEK293 cell membrane homogenates (45 µg/mL). The plot represents the competitive displacement of the radioligand agonist binding from the cell surface CB1R in the presence of increasing concentrations (10−12–10−5 M) of the prototypic CB1R antagonist rimonabant. Data are expressed as a percentage of mean specific binding ± S.E.M. (n = 2)

(B) G protein activation effects of cannabinoid agonist CP55,940 ligand in a [35S]GTPγS binding assay using CB1R expressing HEK293 cell membrane homogenates (20 µg/mL). The plot represents the functional activation of CB1R in HEK293 cell membranes in the presence of increasing concentrations (10−11–10−5 M) of CB1R agonist CP55,940. Data are expressed as a percentage of mean specific binding ±S.E.M. (n ≥ 3)

(C) CB1R antibody optimization for flow cytometry was performed in the functionally CB1R expressing HEK293 cell line in comparison to the IgG isotype control

(D) Fibrosis (14 days post-bleomycin)-induced alterations in different myeloid populations, showing an increase only in the macrophage population in the lungs (one-way ANOVA, ***p < 0.001, n = 6 per group)

(E) Changes in different subsets of macrophage population, showing an increase only in the monocyte-derived alveolar macrophage population in the lung due to induction of fibrosis (one-way ANOVA, ****p < 0.0001, *p < 0.05, n = 6 per group)

Supplementary Figure 7

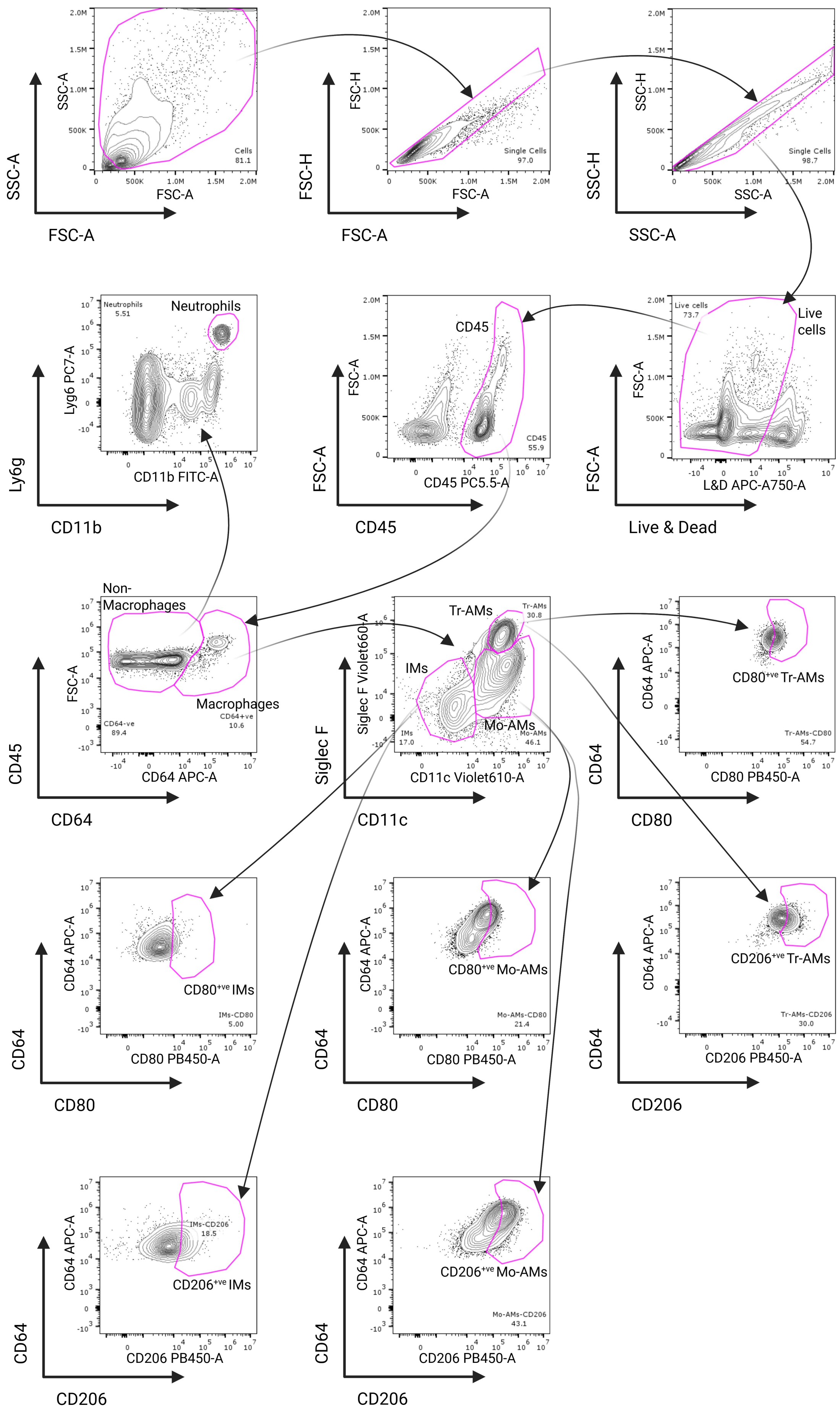

Supplementary Figure 7. Gating strategy to distinguish different subsets of myeloid cells in the mouse lung
Cells were isolated from enzymatically digested mouse lungs, and after the exclusion of doublets and debris, immune cells were identified by CD45 staining. First CD45+ve cells were subdivided into CD64+ve (macrophage) and CD64-ve (non-macrophage) populations. CD11b vs Ly6g was plotted from the CD64-ve population to identify neutrophils (CD11b+ve, Ly6g+ve/hi). CD11c vs Siglec F was plotted from the CD64+ve (macrophage) population to distinguish between Tr-AMs (CD11c+ve, Siglec Fhi), Mo-AMS (CD11C+ve, Siglec F+ve/lo), and IMs (CD11c-ve, Siglec F-ve). From these three subpopulations of macrophages, CD80+ve and CD206+ve cells are gated according to the fluorescence minus one (FMO) control.

Supplementary Figure 8

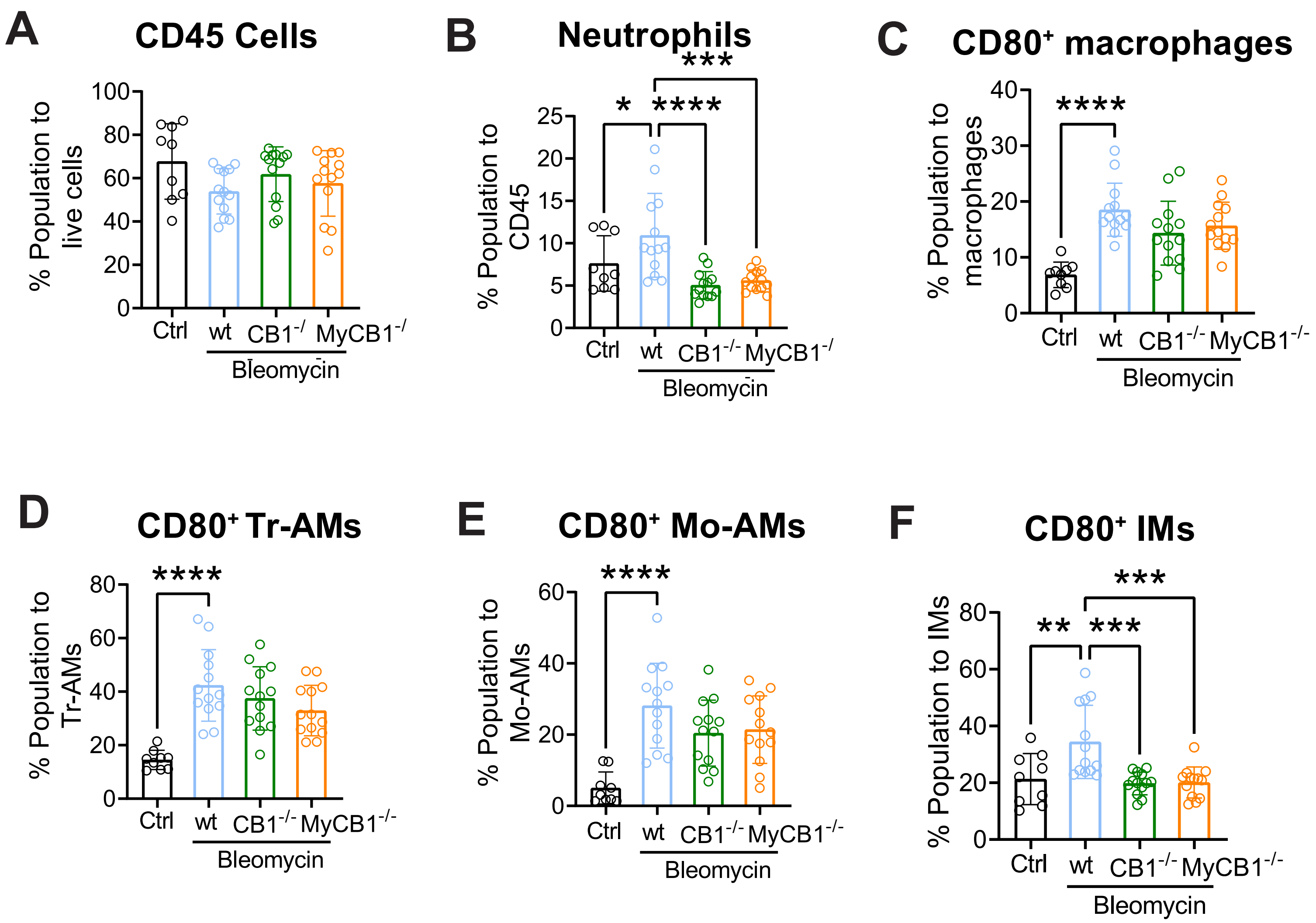

Supplementary Figure 8. Phenotypic alterations of immune cell populations by deletion of CB1R
(A) Total immune population in different groups (one-way ANOVA, n = 10–13 per group).
(B) Neutrophils in different groups (one-way ANOVA, ****p < 0.0001, *p < 0.05, n = 10–13 per group).
(C) CB1R deletion could not modulate CD80+ve macrophages that were increased by bleomycin (one-way ANOVA, ****p < 0.0001, n = 9–13 per group).
(D-F) The subpopulation of CD80+ve macrophages- Tr-AMs, Mo-AMs, and IMs in different groups. Deletion of CB1R did not have any effect on these populations except IMs (one-way ANOVA, ****p < 0.0001, **p < 0.01, n = 9–13 per group).

Supplementary Figure 9

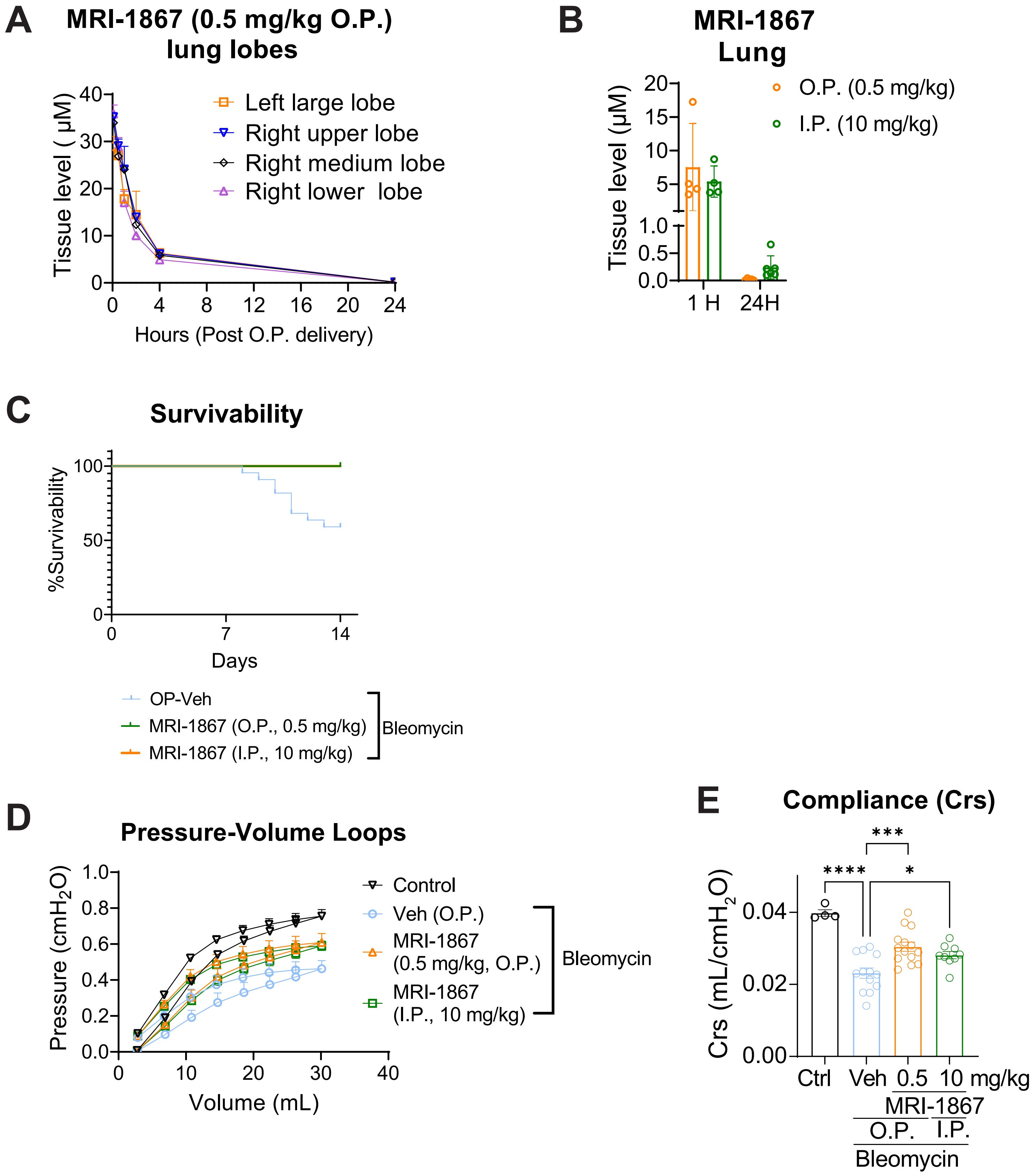

Supplementary Figure 9. Oropharyngeal delivery of MRI-1867 prevented mortality and improved pulmonary functions
(A) Absorption and distribution of MRI-1867 at 0.5 mg/kg b.w. O.P. dose in the different lobes of mice lungs over 24 h (n = 3 per group)
(B) Chronic bioavailability (7 days) study of MRI-1867 at 0.5 mg/kg b.w. O.P. dose and 10 mg/kg b.w. I.P. dose over the therapeutic window of the experimental model (Figure 3F). The concentration of MRI-1867 is almost similar (unpaired t-test, $p > 0.05$, n = 4-6 per group) after 1 h and 24 h of last treatment for both doses
(C) Survivability (Kaplan–Meier plot) among different groups showing no mortality in MRI-1867 treated mice in the bleomycin-induced PF model (n = 9-22 per group)
(D) Changes in the pressure-volume loops (PVP) among different groups (n = 6-7 per group)
(E) MRI-1867 via both O.P. and I.P. dosage showed comparable improvement in compliance (Crs) (one-way ANOVA, ****$p < 0.0001$, ***$p < 0.001$, *$p < 0.05$, n = 4-14 per group)

Supplementary Figure 10

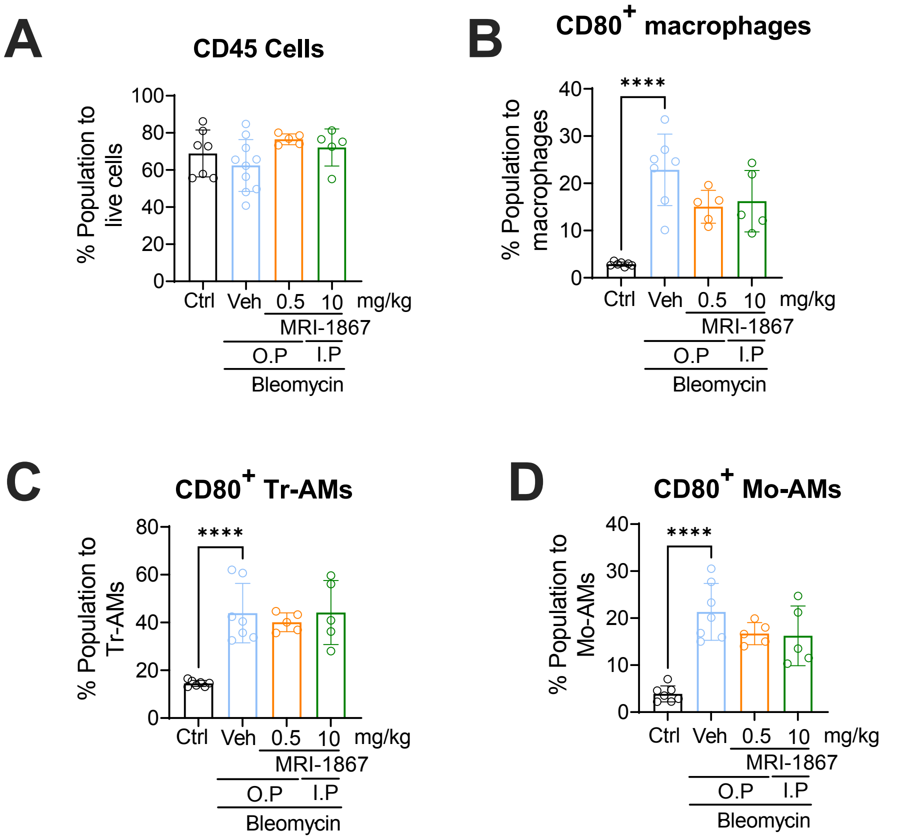

Supplementary Figure 10. Phenotypic characterization of CD80+ve macrophages in the lungs
(A) Total immune population in different groups (one-way ANOVA, n = 5–10 per group).
(B) MRI-1867 could not modulate CD80+ve macrophages that were increased by bleomycin (one-way ANOVA, ****$p < 0.0001$, n = 5–7 per group).
(C-E) The subpopulation of CD80+ve macrophages- Tr-AMs, Mo-AMs, and IMs in different groups. MRI-1867 did not have any effect on these populations (one-way ANOVA, ****$p < 0.0001$, **$p < 0.01$, n = 5–7 per group).

Supplementary Figure 11

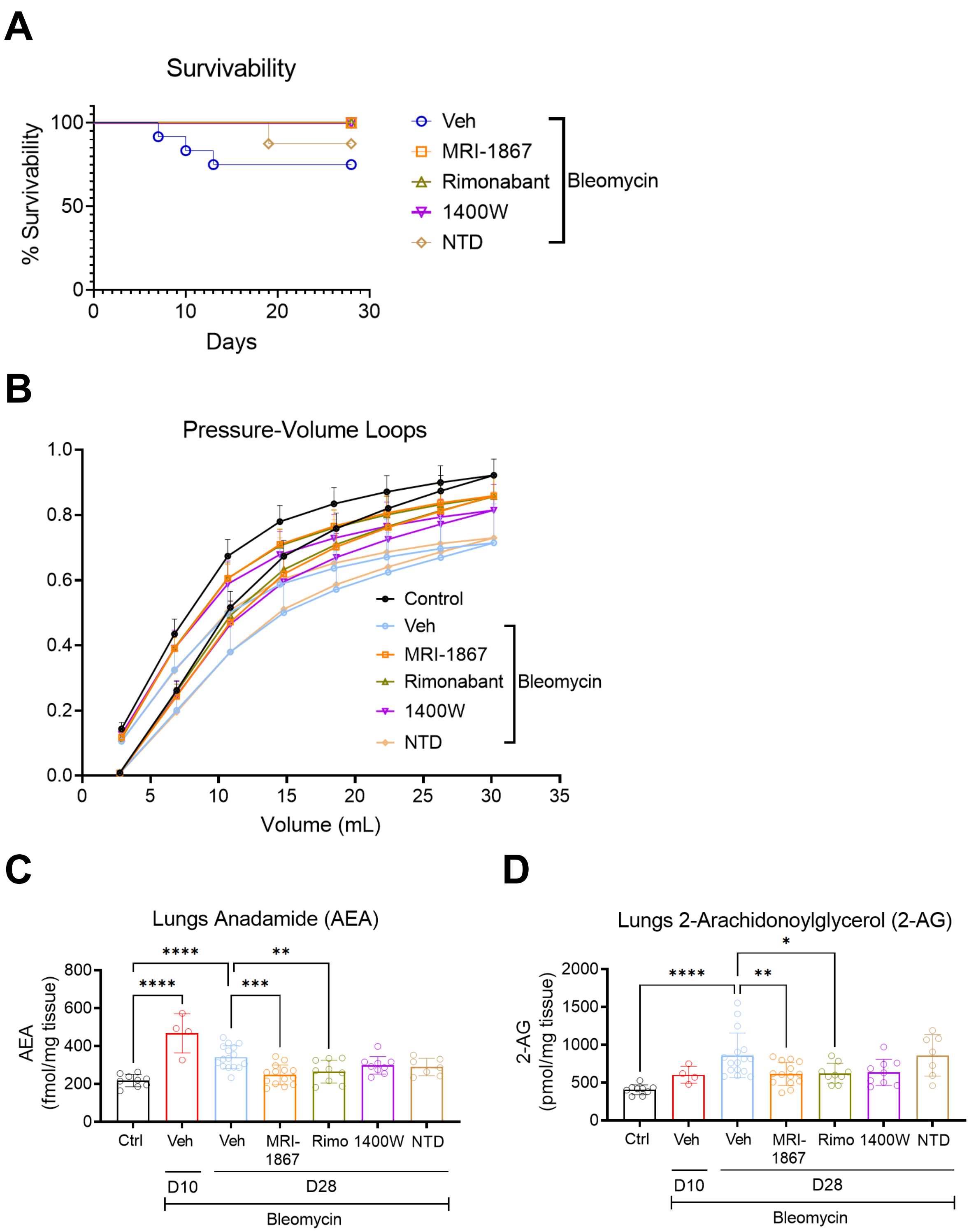

Supplementary Figure 11. Dual targeting of CB1R and iNOS by MRI-1867 prevented mortality and improved pulmonary function

(A) Survivability (Kaplan–Meier plot) among different groups showing mortality in different treatment groups (n = 8–12 per group).

(B) Changes in the pressure-volume loops (PVP) among different groups (n = 8–15 per group).

(C-D) Regulation of endocannabinoids AEA and 2-AG in the lungs by MRI-1867 (one-way ANOVA, ****$p < 0.0001$, **$p < 0.01$, *$p < 0.05$ n = 7–16 per group).

Supplementary Figure 12

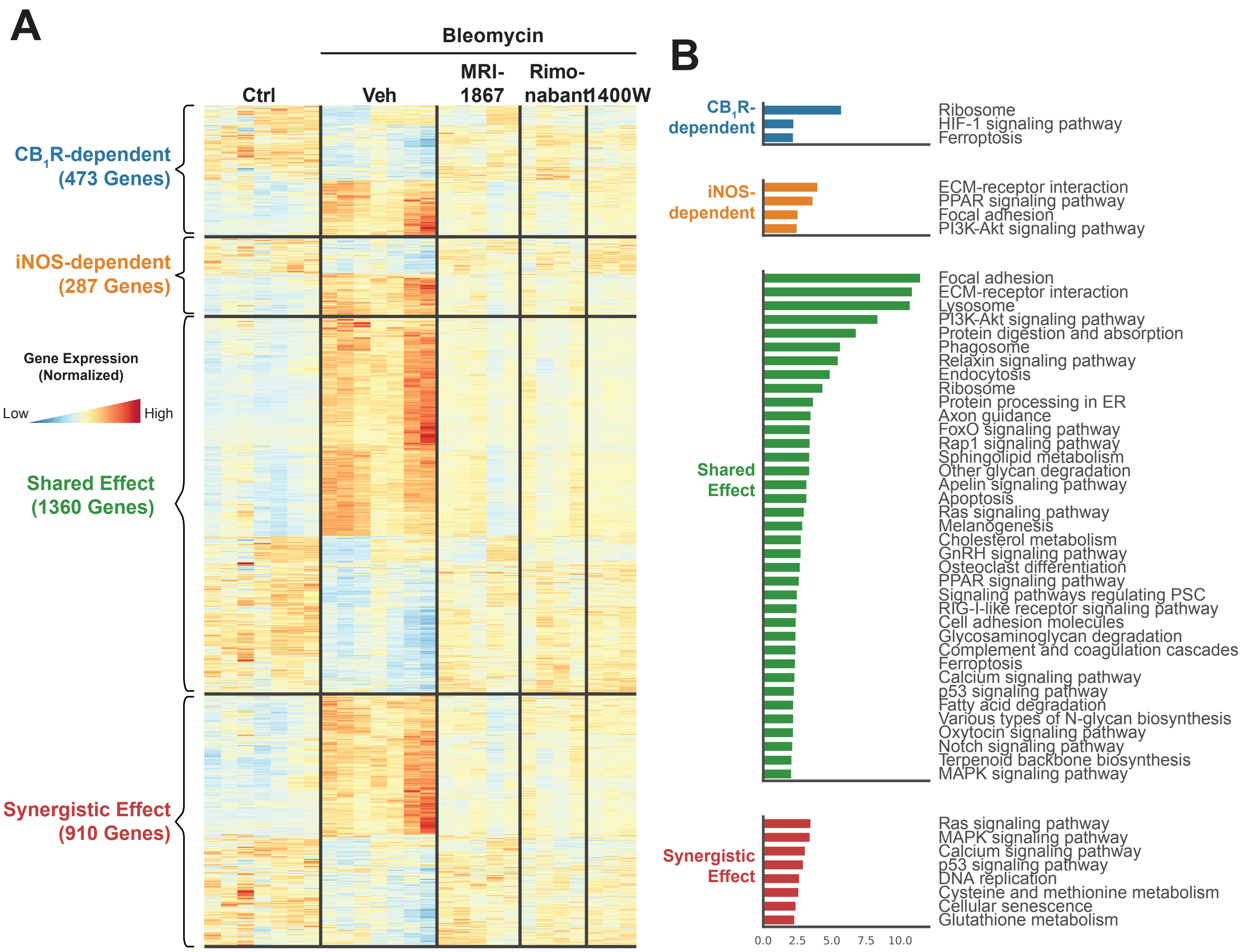

Supplementary Figure 12. Transcriptomics analysis reveals the target-specific and synergistic effects of MRI-1867 on attenuating multiple fibrosis-related genes and pathways

(A) Heatmap showing the differentially expressed genes that were significantly altered in the vehicle (bleomycin) group (n = 7) compared to the control group (n = 7) and reversed by MRI-1867 (n = 5). Rimonabant (n = 4) and 1400W (n = 3) treatments were included to identify the CB1R-dependent, iNOS-dependent, shared, and synergistic effects of MRI-1867 (DESeq2, FDR < 0.05).

(B) Significantly associated pathways (FDR < 0.05) with genes in CB1R-dependent, iNOS-dependent, shared, and synergistic effects of MRI-1867 showing dual targeting elicited much broader response.

Supplementary Figure 13

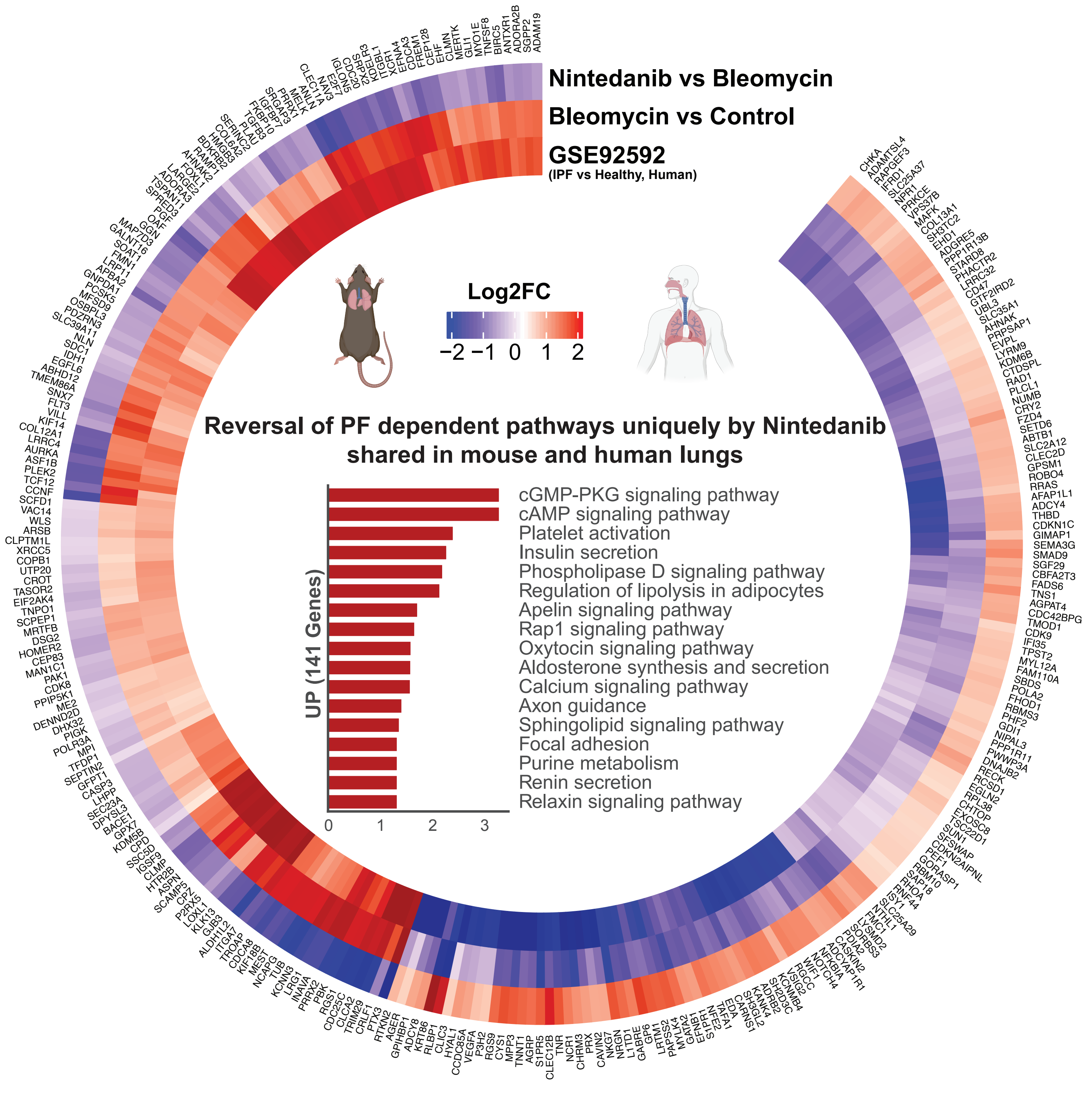

Supplementary Figure 13. Unique regulation of fibrosis-related genes by nintedanib treatment in lung transcriptome in bleomycin-induced pulmonary fibrosis

Circos plot exhibiting the uniquely reversed effects of bleomycin by nintedanib treatment but not by MRI-1867 compared to human late-stage IPF patients' lung transcriptomics data (GSE92592, 39 samples) and the reversed pulmonary fibrosis dependent biological pathways. (DESeq2, FDR < 0.05).

Supplementary Figure 14

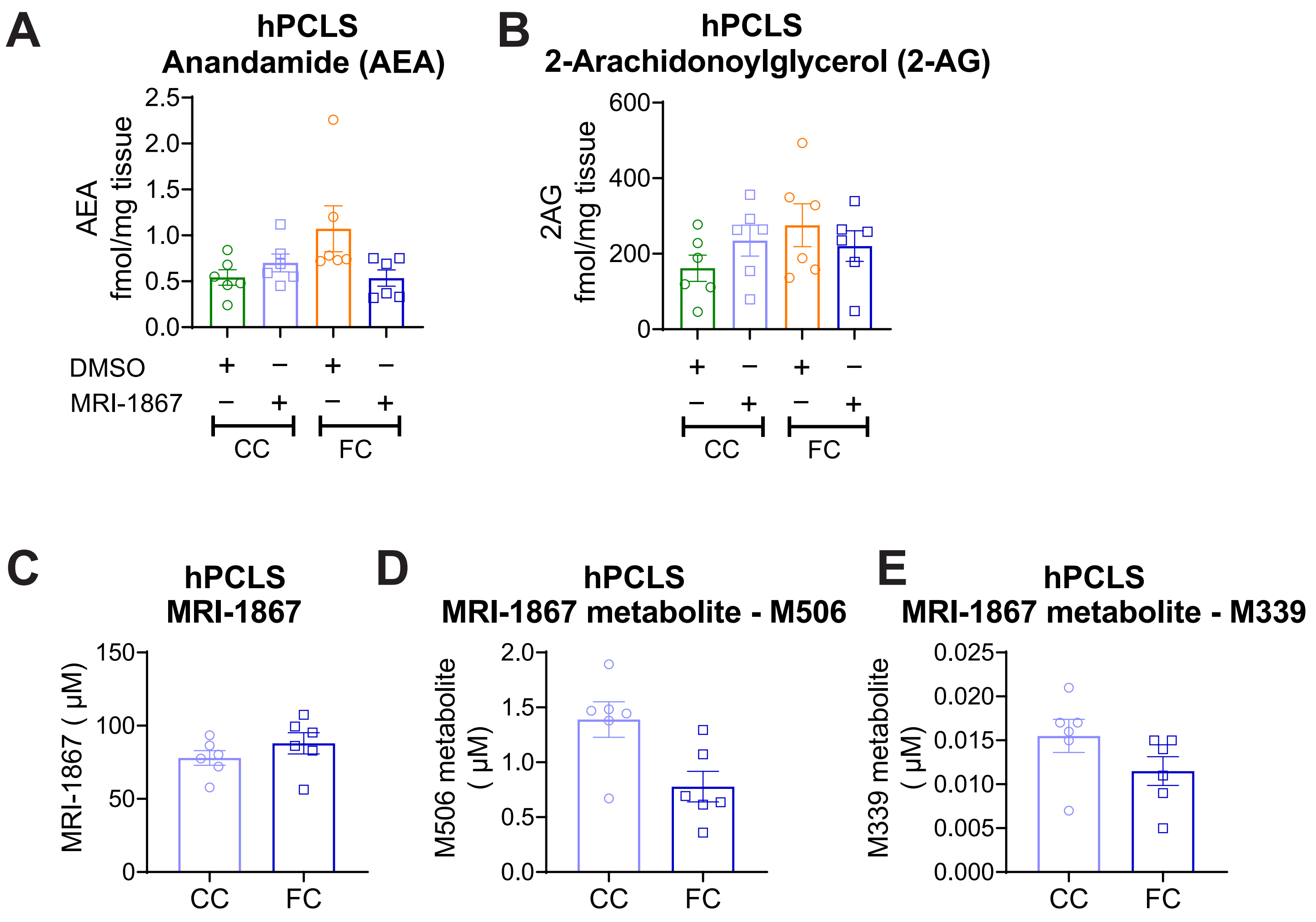

Supplementary Figure 14. Modulation of endocannabinoids by MRI-1867 in human precision-cut lung slices (hPCLS)
(A-B) Modulation of endocannabinoids AEA and 2-AG by MRI-1867 (10 μM), CC: control cocktail, FC: fibrotic cocktail (n = 6 per group)
(C-D) Level of MRI-1867 (10 μM) and its metabolites (M506 and M339) in hPCLS (n = 6 per group)
